# Extracellular vesicle-derived miR-144 as a novel mechanism for chronic intermittent hypoxia-induced endothelial dysfunction

**DOI:** 10.7150/thno.69035

**Published:** 2022-05-16

**Authors:** Huina Zhang, Lu Peng, Yifan Wang, Wen Zhao, Wayne Bond Lau, Yajing Wang, Yu Li, Yunhui Du, Linyi Li, Yu Huang, Shaoping Nie, Yanwen Qin, Xinliang Ma, Yongxiang Wei

**Affiliations:** 1 Beijing An Zhen Hospital, Capital Medical University, Beijing Institute of Heart Lung and Blood Vessel Disease, Beijing, 100029, China.; 2 Department of Biomedical Scienecs, City University of Hong Kong, Hong Kong, 508057, China.; 3 Department of Emergency Medicine, Thomas Jefferson University, Philadelphia, Pa, PA19107, USA.; 4 Department of Emergency, Beijing An Zhen Hospital, Capital Medical University, Beijing, 100029, China.; 5 Department of Otolaryngology, Head and Neck Surgery, Capital Institute of Pediatrics, Beijing, 100020, China.

**Keywords:** Chronic intermittent hypoxia, endothelial dysfunction, extracellular vesicle, erythrocyte, miR144-*Nrf2* Signaling

## Abstract

**Rationale:** Extracellular vesicles (EVs) play a significant role in cell-cell communication. However, whether and how extracellular vesicles are involved in chronic intermittent hypoxia-induced endothelial dysfunction is unknown.

**Methods:** Comparative transcriptomics analysis and miRNA screening were used to identify the possible pathways or target molecules mediating chronic intermittent hypoxia-induced endothelial function. Serum- or erythrocyte-derived EVs were isolated through ultracentrifugation plus filtration. After *in vitro* or* in vivo* treatment with EVs, aortic rings were treated with dihydroethidium staining for superoxidative anion measurement or mounted with wire myography to measure isometric forces. Immunoblotting and qPCR were used for evaluating the molecular mechanism mediating EV miR-144-induced endothelial function under intermittent hypoxia.

**Results:** We revealed a previously undefined importance of circulating extracellular vesicles in regulating endothelial function *via* delivery of miR-144 to endothelial cells, reducing nuclear factor erythroid 2-related factor 2 expression. Additionally, we identified that erythrocytes were the primary cellular source of miR-144-enriched serum-derived extracellular vesicles and that erythrocyte-derived extracellular vesicles were largely responsible for chronic intermittent hypoxia-impaired endothelial function. Furthermore, silencing of miR-144 by anti-miR-144 confirmed its essential role in endothelial dysfunction elicited by erythrocyte-derived extracellular vesicles from chronic intermittent hypoxia-exposed C57BL/6 mice.

**Conclusion:** The results expand the scope of blood-borne substances involved in vascular homeostasis and suggest that anti-miR-144-loaded extracellular vesicles may represent a promising therapeutic approach against obstructive sleep apnea or chronic intermittent hypoxia-associated endothelial dysfunction.

## Introduction

Obstructive sleep apnea (OSA), hallmarked with chronic intermittent hypoxia (CIH) due to recurrent partial or complete pharyngeal collapse during sleep, is a highly prevalent chronic sleep disorder. OSA is identified as an independent risk factor for the development of systemic hypertension. Approximately 50% of OSA patients are hypertensive and an estimated 70-85% patients with resistant hypertensive patients have OSA 1. However, the precise mechanisms underlying OSA-induced hypertension are only partially understood. Growing evidence has revealed that vascular endothelial dysfunction, characterized by impaired endothelium-dependent relaxations, is the earliest sign of vascular injury preceding the occurrence of clinically obvious cardiovascular complications in OSA 2. Therefore, a holistic analysis of the molecular mechanism of endothelial dysfunction under CIH status will help to fully understand the pathogenesis of OSA-or CIH-associated hypertension.

Oxidative stress leads to endothelial dysfunction by promoting NO uncoupling. Anti-oxidative transcription factor NRF2 preserves endothelial function and prevents Ang II-induced hypertension 3. It has been reported that CIH induced myocardial injury by inhibiting NRF2 protein expression 4, but whether NRF2 mediates CIH-related endothelial dysfunction and hypertension is still obscure.

The secretion of extracellular vesicles (EVs) into the blood or other body fluids is a universal cellular process that occurs in multicellular organisms. EVs, as a vital mediator of intercellular communication, perform multifaceted functions by delivering complex molecules to recipient cells, thus participating in the regulation of multiple physiological and pathological process 5. Endothelial cells are the primary cell type known to take up EVs. Circulating EVs, which are in direct and constant contact with endothelial cells, can be absorbed by endothelial cells, thereby affecting the regulation of angiogenesis 6, vascular permeability 7, and vascular tone 8. Previous literature documented that circulating exosomal miR-144-3p inhibited the mobilization of endothelial progenitor cells and then impaired neovascularization in diabetes-related myocardial infarction 9. However, few studies concerned the effect and detail the mechanism of circulating EVs on CIH-related endothelial dysfunction and hypertension.

In this study, we examined the effects of serum-derived EVs as well as erythrocyte-derived EVs from CIH-exposed C57BL/6 mice on endothelial-dependent relaxation and elucidated the underlying mechanisms. This research will help to further understand OSA- or CIH-related endothelial dysfunction and hypertension from a new perspective.

## Methods

The data that support the findings of this study are available from the corresponding author upon reasonable request. See Data Supplement for detailed methods.

### Animal studies

Experiments were approved by Capital Medical University Animal Experimentation Ethics Committee and in compliance with the National Institutes of Health Guidelines on the Use of Laboratory Animals. C57BL/6 mice were treated under normoxia or chronic intermittent hypoxia as previous report 10. Endothelial dependent dilation to acetylcholine was measured.

### Molecular biology

EV was isolated from serum and red blood cells as previous reports 8, 11.

### Statistics

Statistical analysis is summarized in the figure legends. In most cases, the results represent the mean ± standard error of the mean (SEM) of n separate experiments. Concentration-response curves were compared by two-way analysis of variance (two-way ANOVA) followed by Bonferroni post hoc test. Two-tailed Student's t-test was used when two groups were compared. One-way analysis of variance (ANOVA) was performed to determine whether there was a significant difference between more than two datasets, followed by Bonferroni's post hoc test. P < 0.05 indicates statistical difference between groups.

## Results

### Chronic intermittent hypoxia treatment induces endothelial dysfunction, promotes superoxide anion radical generation, and inhibits NRF2 expression

**Figure 1:**
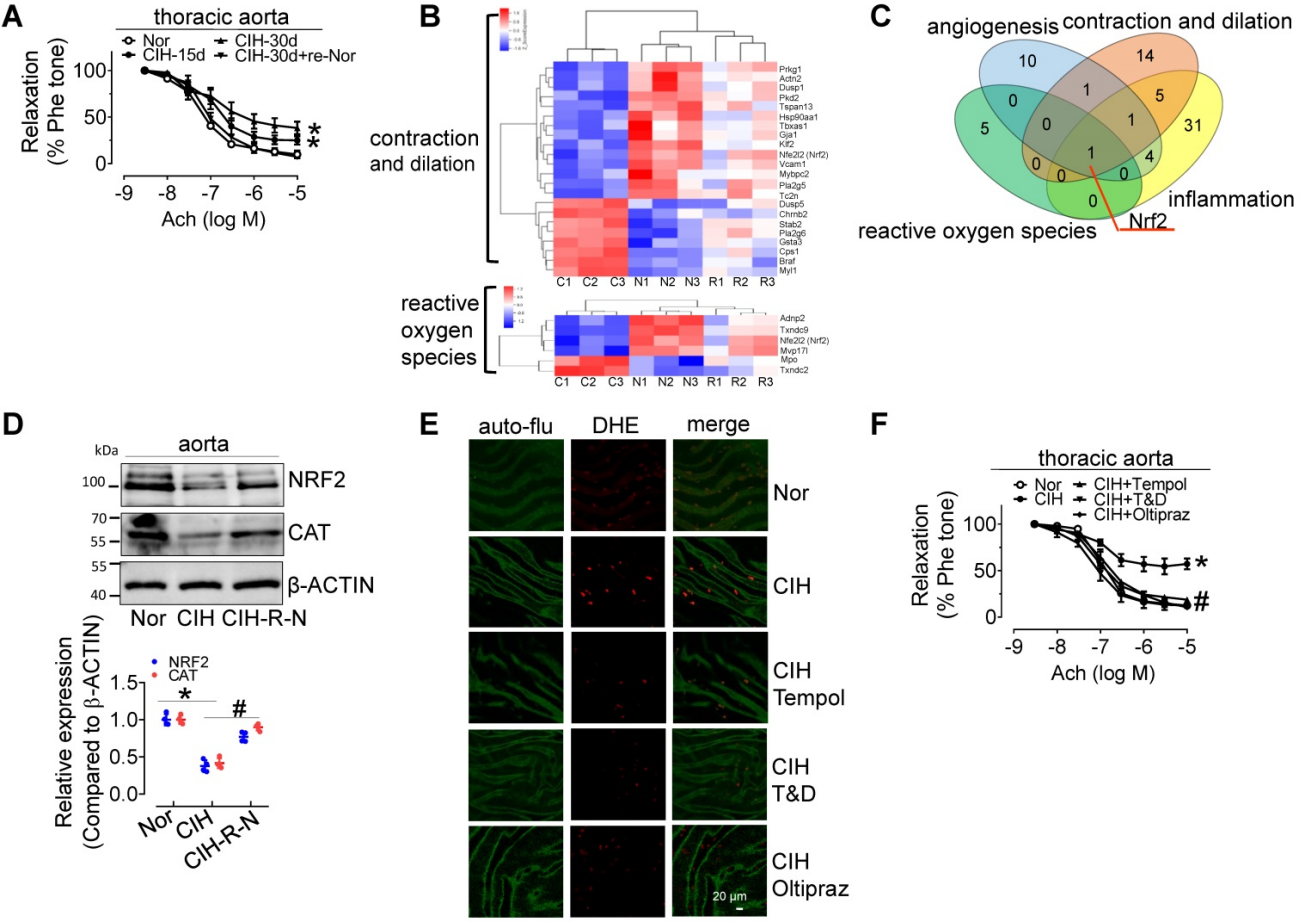
**CIH treatment impairs EDR, reduces NRF2 expression, and promotes superoxide anion production. (A)** CIH treatment impaired EDR in mouse thoracic aortas in a time-dependent manner. **(B)** Some differentially expressed genes in aortas from C57BL/6 mice treated with normoxia (N), CIH (C), or Re-Nor post-CIH (R) were predicted to be associated with vessel contraction and dilation, and reactive oxygen species according to GO and KEGG analysis. **(C)** A Venn diagram illustrated overlap of genes with predicted functions related to angiogenesis (blue), reactive oxygen species (green), contraction and dilation (orange), and inflammation (yellow). **(D)** CIH-30d treatment reduced the expression of NRF2, and its downstream target, CAT, in C57BL/6 mouse aorta, whereas re-administering normoxia for 15 days after 30-day-CIH treatment restored the decreased expression of NRF2 and CAT induced by CIH. **(E)** CIH-30d treatment increased superoxide anion production in endothelial cells, as detected by *en face* fluorescence with dihydroethidium (DHE) dye (red), which was blocked by 30-min pretreatment with ROS scavengers, Tempol (100 µM), or Tiron (1 mM) and DETCA (0.1 mM) or NRF2 agonist, Oltipraz (0.5 g/kg in saline, gavage once per day for 4 days). Bar, 20 µm. Green autofluorescence (auto-flu), elastic fibers; Red DHE staining, ROS in endothelial cells. **(F)** 30-min pretreatment with ROS scavengers, Tempol (100 µM), Tiron (1 mM) and DETCA (0.1 mM), or 4-day treatment with NRF2 agonist, Oltipraz (0.5 g/kg), reversed CIH-induced endothelial dysfunction in thoracic aortas. Results are the mean ± SEM (n = 4). ^*^P < 0.05 vs. Nor. ^#^*P* < 0.05 vs. CIH (D and F). Two-way ANOVA (A and F) and two-tailed t test (D).

30-day CIH treatment severely attenuated endothelium-dependent relaxation (EDR) in C57BL/6 mouse thoracic aortas (mean relaxation from 92.1% to 56.4%) and carotid arteries (mean relaxation from 87.3% to 61.8%), and significantly elevated blood pressure, especially systolic blood pressure, but the effects were reversed by re-administering normal oxygen for 15 days after 30-day-CIH treatment (Figure 1A and Figure S1A-C). CIH treatment increased blood triglyceride levels, but did not significantly change glucose tolerance (Figure S1D-F). To determine the mechanism underlying CIH-induced endothelial dysfunction, microarray analysis was employed to identify differentially expressed genes in aortas from C57BL/6 mice exposed to normoxia (N), CIH (C), and normoxic conditions for 15 days after 30-day-CIH treatment (Re-Nor post-CIH; R). We identified 743 significantly differentially expressed coding genes (Table S1, https://data.mendeley.com/drafts/zbrmjpdv93) between CIH and normoxia mice, with a concomitant reverse tendency in the Re-Nor post-CIH group. Gene ontology (GO) and Kyoto Encyclopedia of Genes and Genomes (KEGG) enrichment analysis demonstrated the cellular processing and functions of differentially expressed genes (Table S2-3). Based on the functional characterization, all differentially expressed genes to be associated with vessel constriction and dilatation, reactive oxygen species (ROS) generation, inflammation, and angiogenesis were manually categorized and presented with a heat map (Figure 1B and Figure S2A-B). Of note, a Venn diagram revealed only one gene, nuclear factor erythroid 2-related factor 2 (*Nrf2*), to be implicated in all four of the above-mentioned functions (Figure 1C). Consistently, Western-blotting results showed that the levels of NRF2 and its downstream target, CATALASE (CAT) were significantly reduced in the aorta from 30-day CIH-exposed C57BL/6 mice and restored in Re-Nor post-CIH group (Figure 1D). Meanwhile, 30-day CIH treatment increased the mRNA levels of NADPH oxidase (NOX) complex subunits, *Nox2* and *p47^phox^* (Figure S2C). Correspondingly, increased superoxide production was clearly demonstrated in aortic endothelial cells from CIH-treated mice detected by *en face* fluorescence with dihydroethidium (DHE) staining, which was reversed by acute 30-min exposure to the ROS scavengers, Tempol (100 μM) or Tiron (1 mM) and DETCA (0.1 mM), as well as by oral treatment with NRF2 agonist, Oltipraz (0.5 g/kg daily in saline *via* gavage for 4 days before execution) (Figure 1E and Figure S2D). Furthermore, administration of Tempol, Tiron and DETCA, and Oltipraz reversed CIH-induced impairment of EDR in C57BL/6 thoracic aorta and carotid arteries (Figure 1F and Figure S2E).

### Serum EVs from CIH-treated C57BL/6 mice (CIH S-EVs) impair endothelial function, augment superoxide anion production, and decrease NRF2 expression

**Figure 2:**
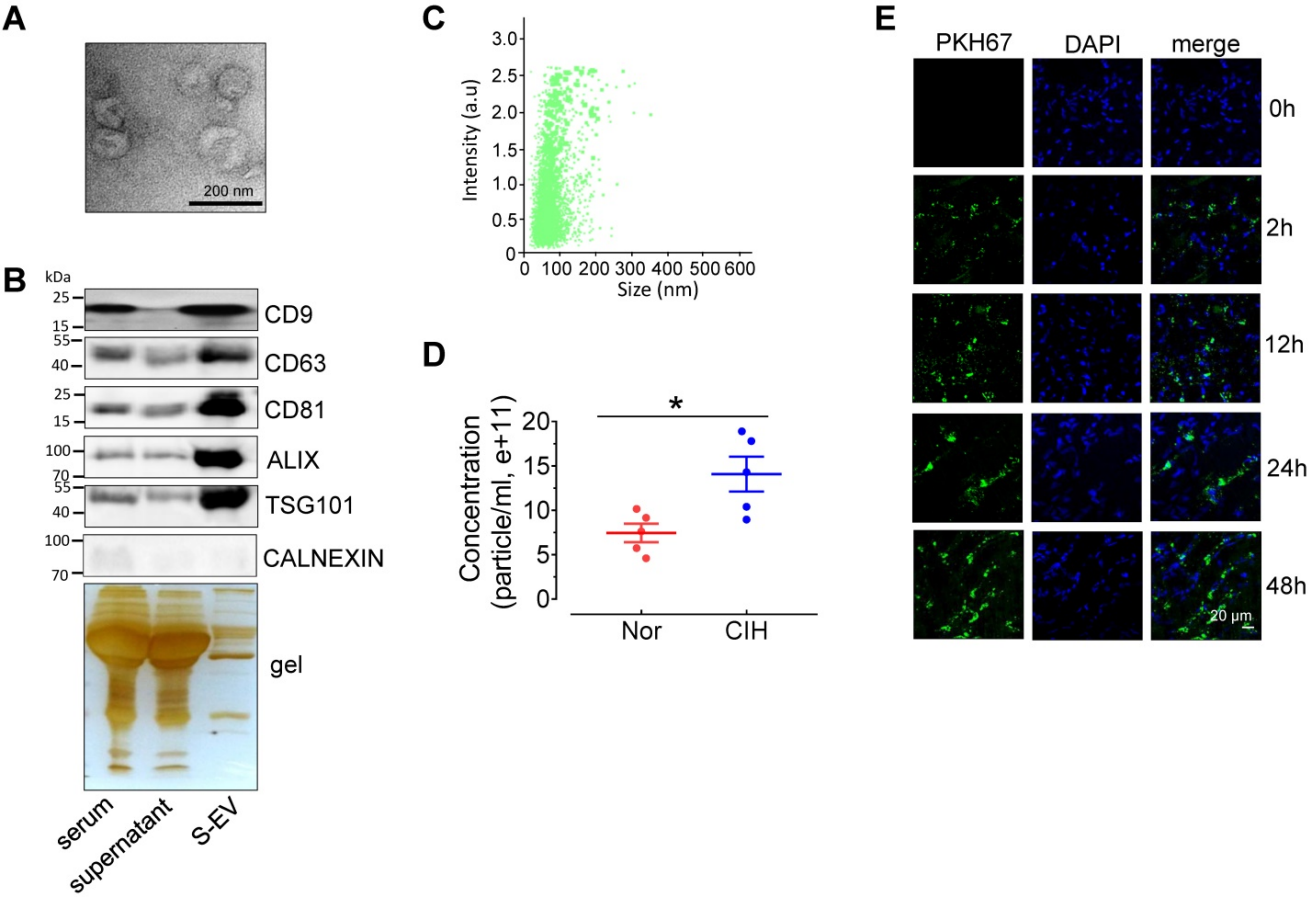
** Extracellular vesicles isolated from C57BL/6 mouse serum can be absorbed by aortic endothelial cells. (A)** Electron microscopy image of whole-mount EVs purified from serum extracellular vesicles (S-EVs). Bar, 200 nm. **(B)** Enrichment of extracellular vesicle markers, CD63, CD81, CD9, ALIX, TSG101, and Calnexin in different serum protein fractions shown by Western blotting. Silver staining demonstrates protein loading and the protein profile of each sample. **(C)** S-EV size (111.0 ± 57.2 nm) was analyzed by NanoSight NS300. **(D)** The concentration of S-EVs was measured by NanoSight NS300. **(E)** Time-dependent uptake of S-EVs by mouse aortic endothelial cells assessed by *en face* staining. Nuclei were stained blue with DAPI. S-EVs were stained green by PKH67. Bar, 20 µm. Results are the mean ± SEM (n = 4-5). ^*^P < 0.05 vs. Nor. Two-tailed t test (D).

To investigate the effect of CIH S-EVs on EDRs, we first evaluated the characterization of S-EVs. Transmission electron microscopy showed that most S-EVs isolated from normal C57BL/6 mice presented classic disc-shaped vesicles with a diameter around 100nm (Figure 2A). The EV markers, especially exosome associated proteins including CD9, CD63, CD81, ALIX, and TSG101, were enriched in the EV fraction. The endoplasmic reticulum protein, Calnexin, was barely detectable in the EV fraction (Figure 2B). Nanoparticle tracking analysis revealed S-EVs with an average size 111.0 ± 57.2 nm yield by 300 μL serum (Figure 2C), while the amount CIH S-EVs was approximately 2-fold that of the normoxic group (Nor S-EVs) (Figure 2D). *En face* staining demonstrated that PKH67-labeled C57BL/6 mouse S-EVs were taken up by endothelial cells in a time-dependent fashion (Figure 2E).

**Figure 3:**
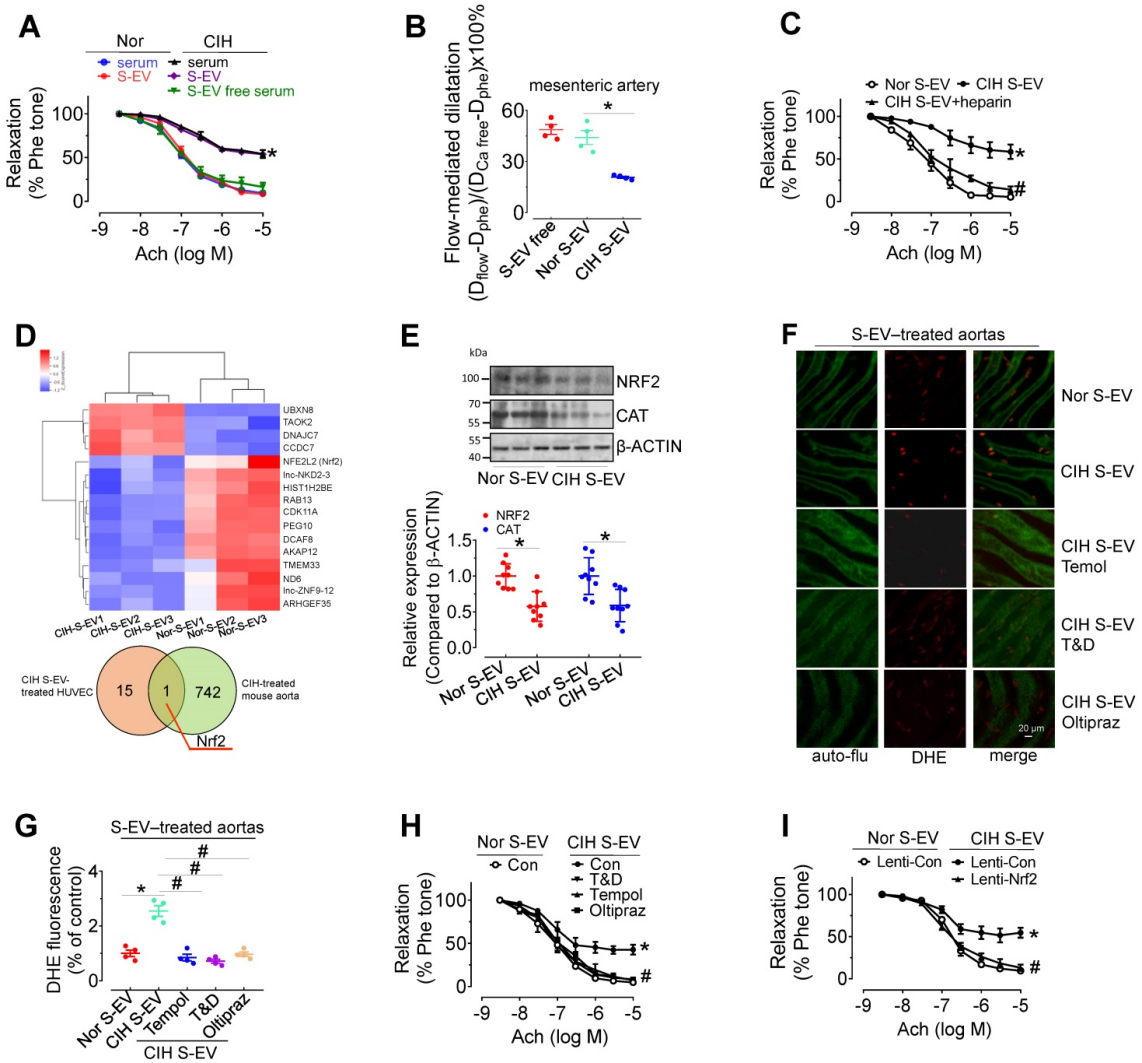
** CIH S-EVs impair endothelial function, augment superoxide anion production, and decrease NRF2 expression in endothelial cells. (A)** 48-h treatment with serum from CIH-treated mouse attenuated EDR in mouse aortas. This effect was absent after removal of EVs from the serum, while 48-h-treatment with CIH S-EVs isolated from 1 mL of blood significantly impaired EDR in mouse aortas. **(B)** Exposure (48 h) to CIH S-EVs reduced flow-mediated dilatation in C57BL/6 mouse mesenteric arteries. **(C)** Heparin (0.3 µg/mL, 48 h) ameliorated EDR induced by CIH S-EVs. **(D)** Transcriptome microarray analysis was used to identify differentially expressed genes in HUVECs treated with CIH S-EVs or Nor S-EVs (up panel). The Venn diagram showed the number of overlapping genes from CIH S-EV-treated HUVECs (orange) and CIH-treated mouse aortas (green) (below panel). **(E)** CIH S-EV reduced the expression of NRF2 and CAT in mouse aortas. **(F-G)** CIH S-EV-incubation increased superoxide anion production in aortic endothelial cells detected by DHE fluorescent dye, which was blocked by 30-min pretreatment with ROS scavengers, Tempol (100 µM), or Tiron (1 mM) and DETCA (0.1 mM), or NRF2 agonist, Oltipraz (100 µM, co-culture for 48 h). Bar, 50 µm. **(H)** 30-min pretreatment with ROS scavengers, Tempol (100 µM), or Tiron (1 mM) and DETCA (0.1 mM), or co-culture with NRF2 agonist, Oltipraz (100 µM) for 48 h reversed CIH S-EV-induced endothelial dysfunction in aortas. **(I)** CIH S-EV-attenuated EDR in mouse aortas was absent after *Nrf2* overexpression mediated by lentivirus. Results are the means ± SEM (n = 4-9). ^*^P < 0.05 vs. Nor S-EV. ^#^*P* < 0.05 vs. CIH S-EV. Two-way ANOVA (A, C, H, I) and two-tailed t test (B, E, G).

*Ex vivo* treatment for 48 h with CIH serum (serum from 1 mL of mouse blood made up to a final volume of 1 mL with serum-free DMEM) attenuated EDR in mouse aortas, whereas EV-free serum treatment did not produce such an inhibitory effect. Direct exposure to CIH S-EVs for 48 h attenuated EDR in C57BL/6 mouse thoracic aortas and decreased flow-mediated dilatation in C57BL/6 mouse mesenteric arteries (Figure 3A-B), but did not affect the endothelium-independent relaxation in mouse aortas (Figure S3A). This effect was more pronounced when aortas were treated with a higher S-EV concentration, or for a longer period (Figure S3B-C). Co-treatment with heparin (0.3 μg/mL, blocker of EV absorption) reversed CIH S-EV-impaired EDR in mouse aortas (Figure 3C). To further ascertain how CIH S-EVs exerts the adverse effect on EDR, transcriptome microarray analysis was used to identify differentially expressed genes in HUVECs treated with CIH S-EVs or Nor S-EVs. 19 significantly differentially expressed coding genes were identified between the two groups (Figure 3D, upper panel, and Table S4, https://data.mendeley.com/drafts/xk3krh5ffp). Among them, *Nrf2* was the only differentially expressed gene common to both CIH S-EV-treated HUVECs and CIH-treated mouse aortas (Figure 3D, below panel). Further verification showed that CIH S-EV *ex vivo* treatment indeed decreased the protein expression of NRF2 and its target, CAT, in both murine endothelial cell line, H5V, (Figure S3D) and mouse aortas (Figure 3E). Furthermore, 48 h of CIH S-EV treatment increased superoxide anion production in H5V cells and in aortic endothelial cells, an effect blocked by 30-min pretreatment with ROS scavengers, Tempol or Tiron and DETCA, or the 48-h cotreatment of NRF2 agonist, Oltipraz (Figure 3F-G and Figure S3E-F). Whereas, the mRNA levels of NOX subunits or Xanthine dehydrogenase (*Xdh*) were not altered in CIH S-EV-treated H5V cells (Figure S3G), indicating that decreased NRF2 was the possible primary molecular responsible for CIH S-EV-induced superoxide anion overproduction in endothelial cells. Consistently, aorta EDR impaired by 48-h CIH S-EVs treatment was reversed by Tempol, Tiron and DETCA as well as NRF2 agonist Oltipraz or lentivirus-mediated NRF2 overexpression (Figure 3H-I and Figure S3H).

### miR-144 is elevated in CIH S-EVs

**Figure 4:**
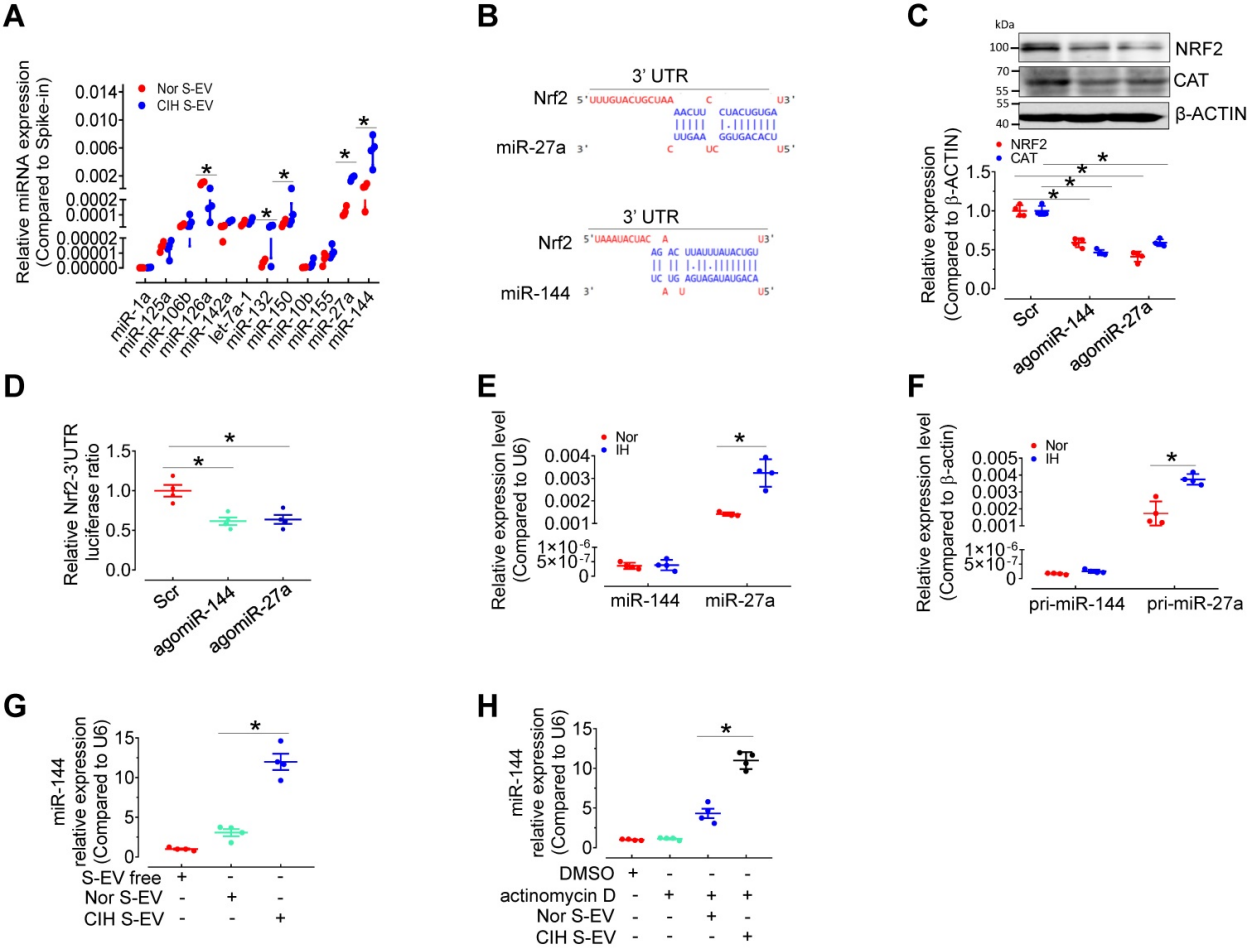
** miR-144 is increased in CIH S-EVs, and is delivered to endothelial cells by S-EVs. (A)** Relative expression level of indicated miRNAs in CIH S-EV or Nor S-EV. **(B)** Diagram depicted the predicted binding sites of miR-27a and miR-144 on the 3'-UTR of *Nrf2*. **(C)** AgomiR-144 and agomiR-27a decreased the expression of NRF2 and CAT in H5V cells. The below panel showed the relative expression level of NRF2 and CAT. **(D)** The treatment of agomiR-144 plus co-transfection of *Nrf2* 3'UTR reporter plasmid led to a significant decrease of the luciferase activity. **(E)** The expression of miR-144 and miR-27a in S-EV-free medium-cultured H5V cells with 24-h normoxia or intermittent hypoxia (IH) treatment. **(F)** Pri-miR-27a and pri-miR-144 were measured by qPCR in EV-free medium-cultured H5V endothelial cells after normoxia or intermittent hypoxia-treatment for 24 h. **(G-H)** The expression of miR-144 in S-EV-free medium-cultured H5V cells with 24-h Nor S-EV or CIH S-EV treatment with (H) or without (G) actinomycin D (10 µg/mL in DMSO) cotreatment. Results are the mean ± SEM (n = 4). ^*^P < 0.05 vs. Nor S-EV (A, G) or Nor S-EV plus actinomycin D or Scr (C, D) or Nor (E, F). One-way ANOVA (D, G, H) and two-tailed t test (A, C, E, F).

We have compared the S-EV protein profiles, but did not identify the possible differentially expressed S-EV protein participating in CIH-induced endothelial dysfunction 12. To determine the mechanism by which CIH S-EVs decreased NRF2 and impaired endothelial function, we measured several EV-associated miRNAs related to oxidative stress or hypoxia signaling 13-19 in S-EVs from normoxia-and CIH-treated mice by qPCR. Levels of miR-126 were decreased while those of miR-132, miR-150, miR-27a, and miR-144 were increased in CIH S-EVs (Figure 4A). Previous reports 20 as well as miRNA target prediction by TargetScan indicated conserved binding sequences for miR-27a and miR-144a in the 3′-UTR of *Nrf2* (Figure 4B). Therefore, we detected the expression regulation of NRF2 by miR-144 and miR-27a in H5V. As expected, overexpression of miR-144 and miR-27a using angomiR-144 and angomiR-27a reduced the protein levels of NRF2 and its target, CAT, in H5V (Figure 4C) and inhibited *Nrf2*-3'UTR-drived luciferase activity in 293A cells (Figure 4D). Furthermore, qPCR results demonstrated that mature miR-27a and its primary transcript pri-miR-27a (but not miR-144 or pri-miR-144), were induced in H5V after intermittent hypoxia (IH) treatment for 24 h (Figure 4E-F), suggesting that miR-27a, other than miR-144 was endogenously expressed in endothelial cells and responded to IH stimulus. miR-144 expression was almost undetectable in H5V cells, but CIH S-EV treatment significantly increased miR-144 abundance in H5V cells (Figure 4G). This effect persisted regardless of whether we inhibited the production of endogenous miRNAs with actinomycin D (10 µg/mL in DMSO) (Figure 4H), suggesting that the endothelial miR-144 signal was mainly from S-EVs. Hence, EV miR-144 was selected as our target molecule for further investigation.

### miR-144 is elevated in erythrocyte-derived extracellular vesicles from CIH-exposed mice (CIH E-EVs)

**Figure 5:**
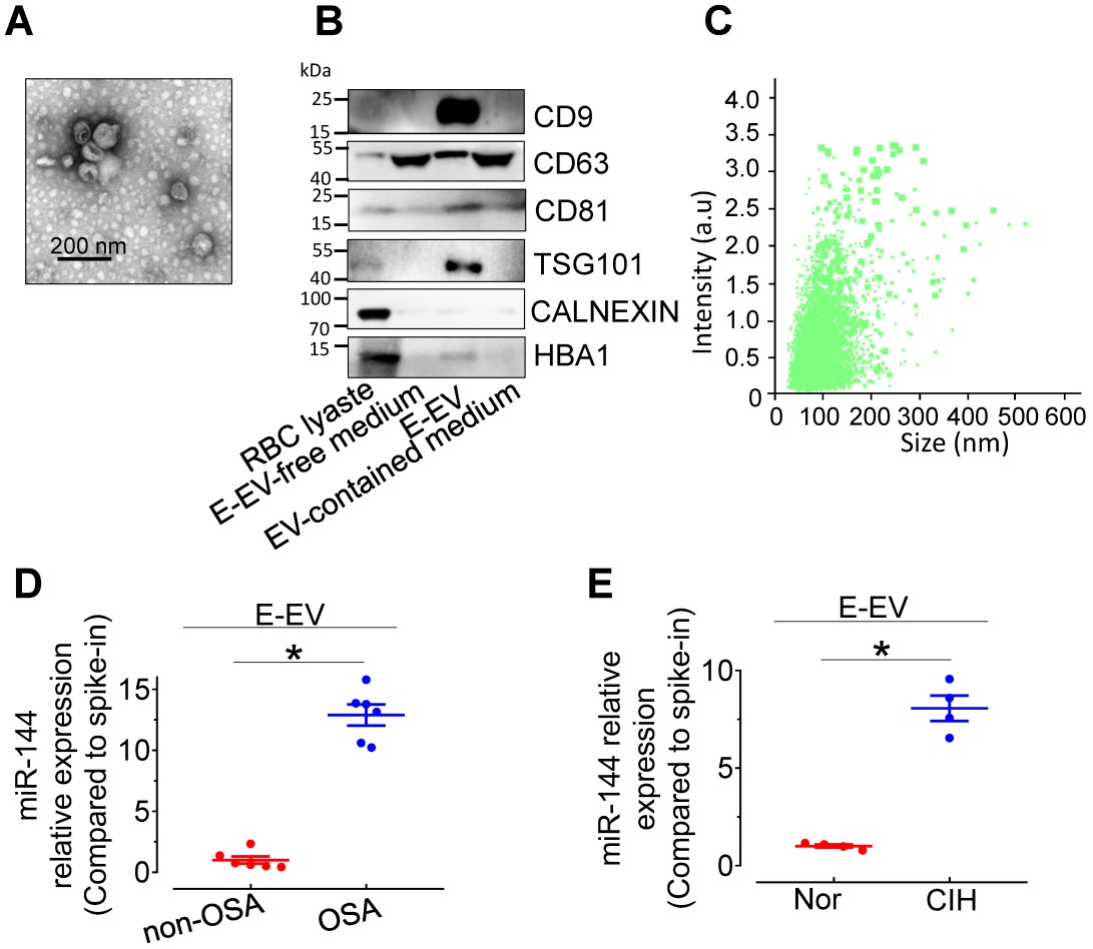
** Characterization of isolated E-EVs and miR-144 expression in E-EVs. (A)** E-EVs isolated from culture medium of mouse red blood cells were imaged by transmission electron microscopy. Bar, 200 nm. **(B)** E-EV size (111.0 ± 57.2 nm) was analyzed by NanoSight NS300. **(C)** Different fractions were processed for Western blotting with EV markers, CD9, CD63, CD81, and TSG101, endoplasmic reticulum marker, Calnexin, and erythrocyte marker, HBA. Silver staining revealed the protein loading amount and the protein profile of each sample. **(D-E)** qPCR assays determined the expression levels of miR-144 in E-EVs from OSA patients (D), CIH-exposed mice (E), and their control groups. Results are the means ± SEM (n = 4-6). ^*^P < 0.05 vs. Nor E-EV or OSA E-EV. Two-tailed t test (D, E).

miR-144 is primarily expressed in erythrocytes and involved in erythroid differentiation 21. To clarify the cellular origin of miR-144 in CIH S-EVs, erythrocyte-derived extracellular vesicles (E-EVs) were isolated from C57BL/6 mouse red blood cells by ultracentrifugation and visualized by transmission electron microscopy. E-EVs presented as classic disc shape (Figure 5A). Nanoparticle tracking analysis showed the average diameter of E-EVs was 115.0±50.9 nm (Figure 5B). The purity of the isolated E-EVs was determined by the relative abundance of E-EV-resident proteins, including CD9, CD81, and TSG101. The erythrocyte protein, HBA, was also observed in the E-EV fraction (Figure 5C). qPCR assay showed that miR-144 was markedly upregulated in E-EVs from both CIH-treated C57BL/6 mouse erythrocytes and OSA patient erythrocytes (Figure 5D-E and Figure S4A-B).

### HIF-1α and GATA1 mediate upregulation of miR-144 in CIH E-EVs

**Figure 6:**
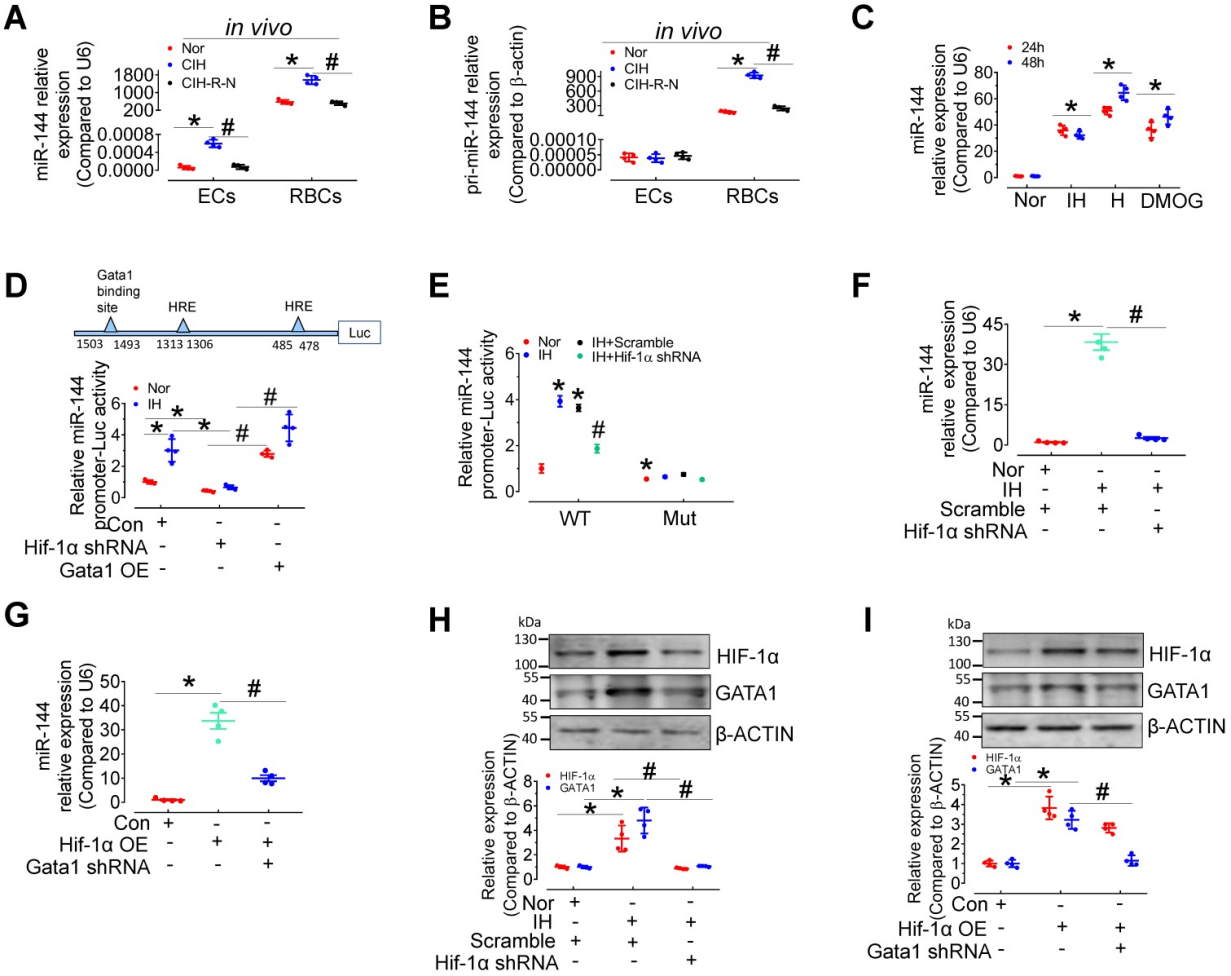
**Expression and regulation of miR-144 in erythrocytes during intermittent hypoxia. (A-B)** qPCR assay measured the expression level of pri-miRNA (A) and miR-144 (B) in aortic endothelial cells and red blood cells in C57BL/6 mice under normoxia, CIH, or readministering normoxia for 15 days after 30-day-CIH treatment (CIH-R-N). **(C)** The expression levels of miR-144 in MEL cells after continuous or intermittent hypoxia or DMOG (1mM in PBS, *Hif-1α* stabilizer) treatment for the indicated period. **(D)** Luciferase reporter gene assays demonstrated the activity of the miR-144 promoter in IH- or normoxia-exposed 293A cells after *Hif-1α* knockdown or *Gata1* overexpression by lentivirus infection. **(E)** Luciferase reporter gene assays demonstrated the activity of the wild-type miR-144 promoter (WT) or GATA1 binding site mutant miR-144 promoter (Mut) in normoxia or IH-exposed 293A cells after lentivirus-mediated knockdown of *Hif-1α*. **(F)** The expression level of miR-144 in IH- or normoxia-treated MEL cells after silencing *Hif-1α*. **(G)** The level of miR-144 in MEL cells after *Gata1* knockdown or *Hif-1α* overexpression. **(H)** The levels of HIF-1α and GATA1 in IH- or normoxia-treated MEL cells after lentiviral silencing of *Hif-1α*. **(I)** The levels of HIF-1α, and GATA1 in MEL cells after *Gata1* knockdown or *Hif-1α* overexpression by lentivirus. Results are the mean ± SEM (n = 4). ^*^P < 0.05 vs. Nor (A-C, E, F, H) or Con (D, G, I). ^#^*P* < 0.05 vs. CIH (A, B) or *Hif*-1α shRNA (D) or IH Scramble (E, F, H) or *Hif*-1α OE (G, I). One-way ANOVA (C-I) and two-tailed t test (A, B).

To provide further evidence of EV-mediated cell-cell communication between erythrocytes and endothelial cells, we measured the levels of pri- and mature miR-144 in red blood cells and endothelial cells from C57BL/6 mice after normoxia, 30-day CIH, or normoxic conditions for 15 days after 30-day-CIH (CIH-R-N) treatment. qPCR results presented that the expression of miR-144 and pri-miR-144 was dominant in erythrocytes rather than in endothelial cells. Under CIH status, the expression of pri-miR-144 and miR-144 was significantly increased in erythrocytes and the tendency was reversed after returning to normoxia for 15 days; whereas, in aortic endothelial cells, only miR-144 was upregulated (Figure 6A-B), indicating that miR-144 in endothelial cells was most likely transferred exogenously from erythrocytes *in vivo*. We further studied the molecular regulation of miR-144 in erythrocytes under hypoxia status. miR-144 was upregulated in murine erythroleukemia (MEL) cells after continuous hypoxia, intermittent hypoxia, or DMOG (1 mM, HIF-1α stabilizer) treatment (Figure 6C). Transcriptional factor hypoxia inducible factor 1α (HIF-1α) plays a central role in hypoxia status. We predicted the localization of two hypoxia response elements (HREs) in the miR-144 promoter sequence and demonstrated that IH treatment increased miR-144 promoter activity, which was blocked by lentivirus-mediated knockdown of *Hif-1α*. Enhanced miR-144 promoter activity by *Gata1* overexpression served as a positive control (Figure 6D). Furthermore, to evaluate the importance of GATA1 and HIF-1α in miR-144 promoter activity, we mutated GATA1 binding site on miR-144 promoter and investigated the effect of intermittent hypoxia and HIF-1α knockdown on the activity of the mutated miR-144 promoter. Results indicated that although intermittent hypoxia significantly induced miR-144 wild type promoter activity, the mutated miR-144 promoter was insensitive to IH stimulation as well as knockdown of *Hif-1α* (Figure 6E), suggesting that GATA1 plays a more critical role than HIF-1α in initiating miR-144 promoter activity. Accordingly, we observed that upregulation of miR-144 by intermittent hypoxia could be reversed by lentivirus-mediated silencing of *Hif-1α* (Figure 6F) and upregulation of miR-144 by HIF-1α overexpression was blocked by knockdown of *Gata1* (Figure 6G). Meanwhile, intermittent hypoxia treatment and overexpression of *Hif-1α* increased GATA1 expression in MEL cells, which was blocked by *Hif-1α* knockdown (Figure 6H-I). These evidences indicated that miR-144 can be directly regulated by HIF-1*α* or indirectly regulated by HIF-1α/GATA1 pathway during hypoxia.

### Anti-miR-144 blocked CIH E-EVs-induced NRF2 expression, superoxide anion production, and endothelial dysfunction

**Figure 7:**
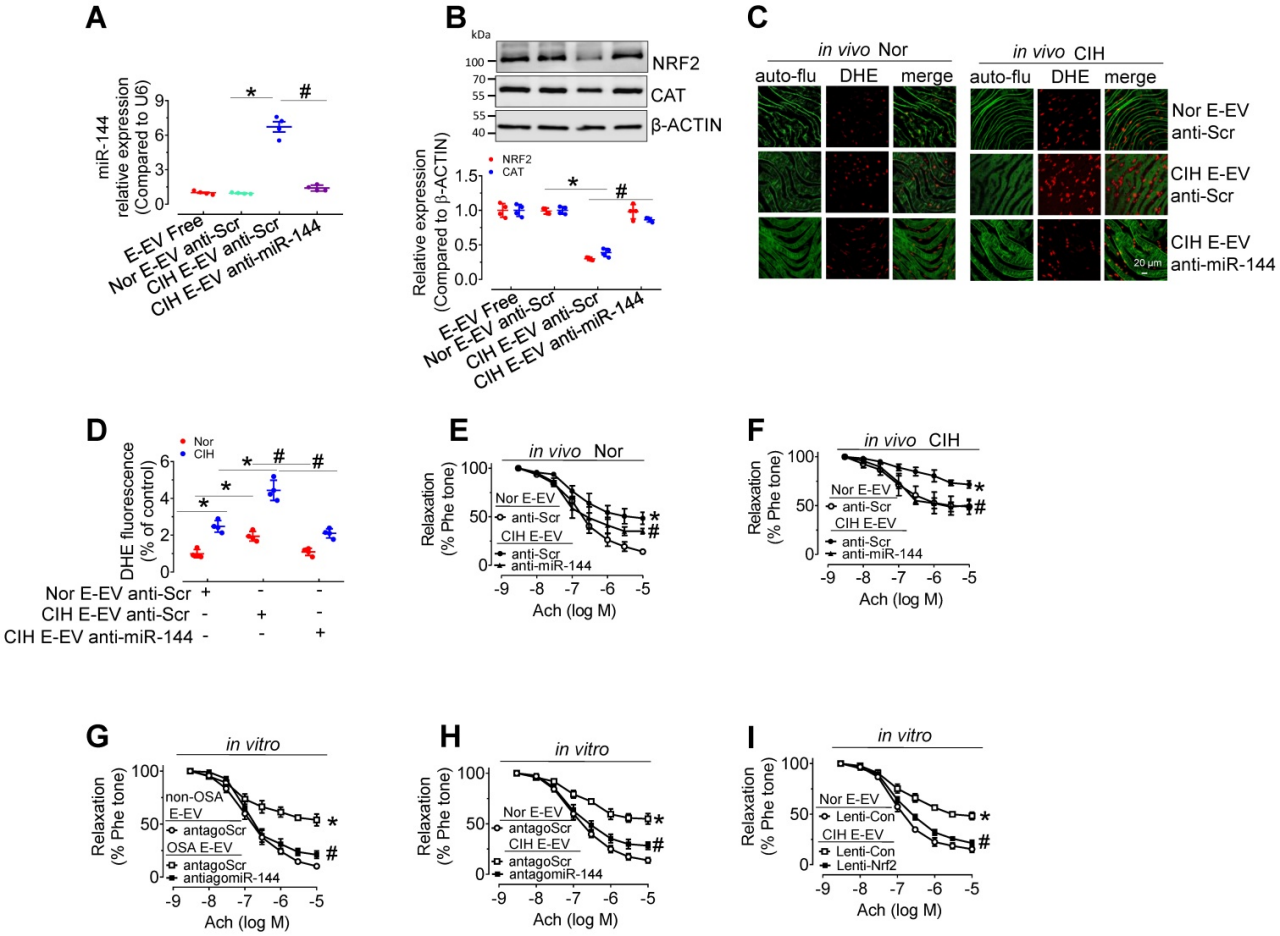
** Anti-miR-144 reverses CIH E-EV-induced superoxide anion overproduction, NRF2 decrease, and endothelial dysfunction. (A)** qPCR analysis verified the expression of miR-144 in H5V cells after the indicated E-EV treatment. **(B)** Protein levels of NRF2 and CAT in H5V cells treated with or without anti-miR-144-loaded CIH E-EVs. Below panel depicted the relative levels of NRF2 and CAT. **(C)** *En face* fluorescence images with DHE staining revealed the superoxide level in aortic endothelial cells after *in vivo* treatment with anti-Scr- or anti-miR-144-loaded CIH E-EVs. Bar, 20 µm. **(D)** Summary of the DHE fluorescence signal intensity. **(E-F)** Anti-miR-144-loaded CIH E-EVs restored CIH E-EV-induced endothelial dysfunction in aortas under normoxia (E) or CIH (F). **(G-H)** antagomiR-144* in vitro* 48-h treatment improved OSA E-EV- (G) or CIH E-EV-(H) induced endothelial dysfunction. **(I)** Lentivirus-mediated overexpression of Nrf2 in mouse aortas reversed CIH E-EV-induced endothelial dysfunction. Results are the mean ± SEM (n = 4). ^*^P < 0.05 vs. Nor E-EV anti-Scr (A, B, D-F) or non-OSA E-EV (G) or Nor E-EV antagoScr (H) or Nor E-EV Lenti-Con (I). ^#^*P* < 0.05 vs. CIH E-EV anti-Scr (A, B, D-F) or OSA E-EV antagoScr (G) or CIH E-EV antagoScr (H) or CIH E-EV Lenti-Con (I). One-way ANOVA (A, B, D) and two-way ANOVA (E-I).

To further verify the endothelial effect of miR-144 delivery by CIH E-EVs, anti-miR-144 as well as anti-miR-144-loaded CIH E-EVs were utilized to treat aortas *in vitro* or treat CIH mice *in vivo*. As expected, anti-miR-144-loaded CIH E-EVs did not increase miR-144 levels in H5V cells after incubation for 48 h, while anti-scramble-loaded groups did (Figure 7A). Protein levels of NRF2 and CAT in H5V cells after 48-h treatment of anti-miR-144-loaded CIH E-EVs were maintained at the level observed in the control groups (Figure 7B). Consistently, *in vivo* CIH E-EV treatment by tail injection (one injection/3 day, for 12 days) potentiated superoxide anion production in endothelial cells. However, this effect was largely inhibited in anti-miR-144-loaded CIH E-EV treated groups, as shown by *en face* DHE staining (Figure 7C-D). Meanwhile, *in vivo* treatment of CIH E-EVs impaired EDR and increased systolic blood pressure in normoxia- and CIH-treated mice. These effects were reduced in groups treated with anti-miR-144-loaded CIH E-EVs (Figure 7E-F and Figure S4C-E). Similarly, antagomiR-144 significantly reversed endothelial dysfunction caused by E-EV from CIH mice or OSA patients (Figure 7G-H). Of note, lentivirus-mediated *Nrf2* overexpression also effectively blocked CIH E-EV-impaired endothelial function (Figure 7I).

## Discussion

This study highlights the role of S-EVs and E-EVs as blood-borne regulators of vascular function, and pinpoints erythrocyte-enriched miR-144 as a critical EV-derived miRNA that participates in CIH-induced endothelial dysfunction via inhibiting NRF2 expression.

OSA is a prevalent disease most frequently associated with secondary hypertension 1. Endothelial dysfunction is the earliest vascular consequence of OSA or CIH and precedes hypertension onset 22, 23. However, the underlying mechanism of OSA or CIH-induced endothelial dysfunction is not well characterized. Some reports propose that changes in vasoactive molecules, such as reduced NO bioavailability 23, excessive oxidative stress 24, increased levels of angiotensin II and endothelin-1 25, 26, regulate endothelial function during the development of hypertension following CIH. Consistently, we confirmed that CIH treatment dramatically impaired EDR in the C57BL/6 mouse aorta and increased systolic blood pressure. To explore the mechanism more comprehensively and systematically, we performed global transcriptome analysis of aortas from CIH-treated mice. Among the 743 differentially expressed genes, *Nrf2* was the only one commonly implicated in the four vascular-associated responses, namely, contraction and dilation, angiogenesis, ROS production, and inflammation. ROS regulates vascular homeostasis, and ROS overproduction-induced oxidative stress is a primary cause of vascular dysfunction by reducing NO availability 27. Increased oxidative stress has been proposed to contribute to OSA or CIH-related endothelial dysfunction and hypertension. NRF2 is a key antioxidant transcriptional factor with a protective role in many free radical detoxification pathways related to aging, atherosclerosis, hypertension, ischemia, and other cardiovascular diseases 28, 29. NRF2 binds to the antioxidant response element to transcriptionally activate downstream genes encoding Glutathione S-transferase, Aldehyde dehydrogenase, CAT, Heme oxygenase 1, and Thioredoxin 30. However, most reports emphasize the role of increased NOX levels and activity in CIH-induced adverse vascular outcomes 24, 31, and few studies have linked NRF2 regulation to CIH-induced endothelial dysfunction, although some studies similarly revealed that CIH inhibited NRF2 expression 4, 32. In the present study, we demonstrated that NRF2 downregulation in CIH-treated mouse aorta or in CIH-treated endothelial cells results in excess superoxide production and impaired endothelial function, an effect rescued by the NRF2 agonist, Oltipraz, or by the ROS scavengers, Tempol or Tiron and DETCA. This result confirms the importance of NRF2 in CIH-associated endothelial dysfunction induced by oxidative stress.

Circulating EVs are constantly in direct contact with and regulate the functions of endothelial cells under pathophysiological conditions 33. EVs from OSA patient plasma impair endothelial adhesiveness and permeability 34. Little attention has been given to the impact of EVs upon CIH-impaired EDR. Flow-mediated dilation was attenuated in mice after treatment with plasma EVs from OSA pediatric patients. This compromised vascular function was proposed to be caused by reduced eNOS expression and altered EV miR-630 35. Our group recently reported that EV derived from intermittent hypoxia-treated red blood cells impaired endothelial function through regulating eNOS phosphorylation and ET-1 expression 11. Here, we demonstrate that EVs-derived from CIH S-EVs and CIH E-EVs profoundly impaired EDR in normal mouse aorta. Interestingly, NRF2 was differentially expressed in CIH S-EV-treated HUVECs and CIH-treated mouse aortas compared with their corresponding controls. CIH S-EVs attenuated NRF2 expression, which increased ROS production in endothelial cells without altering NOX signaling. This was validated by improved endothelial function in response to the NRF2 agonist, Oltipraz.

We previously reported that S-EVs mediate endothelial dysfunction in diabetes through delivery of arginase 1 8. We also compared the S-EV protein profiles between normoxic and CIH animal models, but did not identify the possible differentially expressed S-EV protein participating in CIH-induced endothelial dysfunction 12. Therefore, we focused upon S-EV miRNAs through screening miRNAs related to oxidative stress or hypoxia signaling in S-EVs and found miR-144 and miR-27a was upregulated in CIH S-EVs. miR-144 and miR-27a are known to inhibit NRF2 expression by directly binding to the 3′-UTR of *Nrf2* 20, and we confirmed this regulatory link in endothelial cells. Interestingly, unlike miR-27a, miR-144 is expressed at a very low endogenous level in endothelial cells. The expression of miR-144 or pri-miR-144 in endothelial cells did not change after intermittent hypoxia treatment, suggesting that the increased level of miR-144 in CIH S-EVs was likely to be derived from a non- endothelial origin. miR-144 is highly expressed in erythrocytes and is closely related to erythroid differentiation 21. We confirmed high levels of miR-144 expression in erythrocytes and E-EVs following CIH and in OSA. miR-144 was reported to play versatile roles in maintaining homeostasis in the cardiovascular system. For instance, miR-144 significantly reduced infarct size in an acute ischemia reperfusion injury model and reduces left ventricular remodeling after myocardial infarction through promoting autophagy 36, 37, and protected against LPS-induced lung endothelial hyperpermeability 38; but it also promoted atherosclerosis plaque formation 39, and mediated the 7-Ketocholesterol-induced endothelial dysfunction 40. One research even found that circulating exosomal miR-144-3p impaired the mobilization ability of EPCs and then impaired ischemia-induced neovascularization 9. To clarify the involvement of EV miR-144 in CIH E-EV-induced endothelial dysfunction, we demonstrated that CIH E-EV treatment increased miR-144 expression and reduced NRF2 expression, increased superoxide production in endothelial cells, attenuated aorta EDR, and increased systolic blood pressure. Furthermore, anti-miR-144-loaded CIH E-EVs effectively blocked miR-144 expression and increased the levels of NRF2 and CAT in endothelial cells. More importantly, functional studies revealed that anti-miR-144-loaded CIH E-EVs as well as antagomir-144 partially rescued EDR, reduced ROS, and decreased hypertension caused by CIH E-EVs, indicating a pivotal role of circulating EV miR-144 in these processes. Furthermore, we confirmed the protective role of NRF2 in CIH S-EV- or CIH E-EV-caused endothelial dysfunction through overexpression of Nrf2 by lentivirus.

The regulation of miR-144 in erythrocytes during hypoxia is not well documented in the literature. Sequencing analysis showed that miR-144 was upregulated after 16 h of hypoxic treatment in HUVECs 41. Other papers have shown that miR-144 expression is significantly higher in cancer tissues that are often under hypoxia conditions compared to normal tissues 42, 43. However, none of these articles addressed the mechanism of hypoxia-induced miR-144 upregulation. GATA1 is an important transcriptional factor that increases miR-144 expression during erythropoiesis 44. Here, we predicated a hypoxia response element and a GATA1 binding site in the miR-144 promoter region and confirmed that intermittent hypoxia treatment promoted miR-144 expression in MEL cells *via* HIF-1α direct transcriptional regulation. Interestingly, we also demonstrated that both *Hif-1α* overexpression and CIH treatment upregulated GATA1 expression in MEL cells, indicating GATA1 to be a mediator participating in HIF-1α indirect regulation of miR-144.

The present study has several limitations. In this study, considering that OSA patients may have complex comorbidities and complex pathophysiological states, which will increase the heterogeneity of EVs and even lead to inconsistent results, we selected serum EVs or erythrocyte-derived EVs obtained under more consistent CIH conditions to treat aortic and endothelial cells to obtain more reproducible results. This result reflects the mechanism of EV-induced vascular impairment in CIH setting, however, this mode of treatment is less clinically relevant than EV of OSA patient origin. Meanwhile, given that many of the miRNAs in mouse EVs are not homologous to human ones, our treatment model may therefore miss other possible functional molecules. We provide clear evidence that miR-144 in erythrocyte-derived EVs is responsible for endothelial dysfunction induced by CIH, the major pathological alteration in OSA. However, it is not known whether the same or different EV miRNAs are involved in the endothelial dysfunction induced by other pathological features of OSA, such as fragmented sleep, sympathetic nerve activation, and increased negative chest pressure. Moreover, we cannot exclude the possible involvement of non-erythroid-derived miR-144, albeit to a lesser degree. Further investigation is warranted to address these problems. Meanwhile, considering that mature erythrocytes would not respond to any stimulus to induce the expression of miRNAs, in the current work, we used MEL to reveal the mechanism by which intermittent hypoxia promoted miR-144 expression, which maybe partially helped to understand the increased levels of miR-144 in mature erythrocytes under CIH status, however, the *in vivo* mechanism closer to the physiological state should be in future investigations. Due to technical constraints, the CIH paradigm used in the current study does not closely match that observed in clinical OSA status, new specific interfering tools and more rigorous studies are required to further explore the clinical relevance of S-EV or E-EV in the development of OSA-related vasculopathy. In addition, the present study cannot discount other possible signals, such as miR-27a, that are independent of E-EV miR-144/NRF2 might play a role in CIH-induced impairment of endothelial function, which requires future examination.

In summary, we systematically demonstrate that CIH S-EVs and CIH E-EVs can deliver functional miR-144 to endothelial cells, promoting superoxide anion production by reducing NRF2 expression, a critical pathogenic component of endothelial dysfunction and hypertension during CIH. Our study provides experimental evidence for the therapeutic potential of EV-loaded anti-miR-144 or antagomir-144 in treating OSA or CIH-associated vascular complications.

## Supplementary Material

Supplementary materials and methods, figures, and tables.

## Abbreviations

EVs: extracellular vesicles
CIH: chronic intermittent hypoxia
DHE: dihydroethidium
OSA: obstructive sleep apnea
SEM: standard error of the mean
EDR: endothelium-dependent relaxation
FMD: For flow-mediated dilatation
GO: Gene ontology
KEGG: Kyoto Encyclopedia of Genes and Genomes
ROS: reactive oxygen species
NRF2: nuclear factor erythroid 2-related factor 2
NOX: NADPH oxidase
HIF-1α: hypoxia inducible factor 1α
