# Supplementary material for: Extracellular vesicle-derived miR-144 as a novel mechanism for chronic intermittent hypoxia-induced endothelial dysfunction
Source: Theranostics. 2022 May 16;12(9):4237–49. doi: 10.7150/thno.69035 (PMC9169375; doi:10.7150/thno.69035)
Supplement: Supplementary file 1 — Supplementary materials and methods, figures, and tables. [file thnov12p4237s1.pdf]

Extracellular vesicle-derived miR-144 as a novel mechanism for chronic intermittent hypoxia-induced endothelial dysfunction

Huina Zhang[1,*], Lu Peng[1], Yifan Wang[2], Wen Zhao[1], Wayne Bond Lau[3], Yajing Wang[3], Yu Li[1], Yunhui Du[1], Linyi Li[1], Yu Huang[2], Shaoping Nie[4], Yanwen Qin[1], Xinliang Ma[3], Yongxiang Wei[5]

[1]Beijing An Zhen Hospital, Capital Medical University, Beijing Institute of Heart Lung and Blood Vessel Disease, Beijing, 100029, China;
[2]Department of Biomedical Scienecs, City University of Hong Kong, Hong Kong, 508057, China;
[3]Department of Emergency Medicine, Thomas Jefferson University, Philadelphia, Pa, PA19107, USA;
[4]Department of Emergency, Beijing An Zhen Hospital, Capital Medical University, Beijing, 100029, China;
[5]Department of Otolaryngology, Head and Neck Surgery, Capital Institute of Pediatrics, Beijing, 100020, China;

Short title: Extracellular vesicle and vascular function

Huina Zhang, Beijing An Zhen Hospital, Capital Medical University, Beijing Institute of Heart Lung and Blood Vessel Disease, No. 2 Anzhen Road, Beijing, 100029, China, Tel.: +86-10-64456509, E-mail: whinnerzhn@mail.ccmu.edu.cn

Supplementary Materials and Methods

Mice

All animal experiments were approved by Capital Medical University Animal Experimentation Ethics Committee, and in compliance with the National Institutes of Health Guidelines on the Use of Laboratory Animals. C57BL/6 mice were purchased from Beijing Vital River Laboratory Animal Technology Co. Twelve-week-old male mice were used. Unrestrained, freely moving mice were individually housed in chambers and received CIH treatment with fractional oxygen concentration cycled between 5% and 21% every 3 min, 8-10 h during the daytime for 15 or 30 days using a BioSpherix OxyCycler A84 system (BioSpherix, Redfield, NY, USA). Control mice were maintained in normoxic conditions for the same duration. Re-Nor post-CIH-treated mice were kept under normoxic conditions for 15 days after 30-day CIH treatment. For euthanasia, C57BL/6 mice were anesthetized with 5% isoflurane followed by terminal cardiac bleed before EV isolation, artery dissection and biochemical parameter measurement. Diastolic (DBP) and systolic (SBP) blood pressures were measured by tail-cuff plethysmography (BP-2000; Visitech Systems, Apex, North Carolina, USA).

Clinical Samples

The study design was approved by the Medical Ethics Committee of Beijing Anzhen Hospital (2017005) and conformed to the principles outlined in the Declaration of Helsinki. The research protocols were performed in accordance with the Board of Ethics at Beijing Anzhen Hospital. Written informed consent was obtained from all participants. All the subjects enrolled in this study were attending the department of Otolaryngology-Head and Neck Surgery, Beijing Anzhen Hospital, Capital Medical University. All clinical samples and clinical information were collected at Beijing Anzhen Hospital. All patients (aged 42–67 years) were male and underwent baseline anthropometric and blood pressure assessments, as well as overnight polysomnography detection, which was interpreted using standard approaches. All fasting blood samples for erythrocyte-derived EV (E-EV) purification were collected with standard EDTA anticoagulation. The inclusion criteria for the OSA group were: participants polysomnographically diagnosed with moderate or severe OSA (apnea-hypopnea index (AHI)≥15/h) (n=6). The exclusion criteria for the OSA group were: patients with hyperglycemia, cancer and infection, chronic obstructive pulmonary disease, chronic bronchitis, restrictive lung diseases, respiratory failure, aortic coarctation, Cushing syndrome, pheochromocytoma, primary aldosteronism, renal parenchymal disease, renovascular hypertension, thyroid disorders, stroke, coronary heart disease, and those who could not provide a complete medical history. The inclusion criterion for the non-OSA group was: AHI < 5/h (n=6), All participants were male. The exclusion criteria for the non-OSA group were: subjects with hyperglycemia, cancer and infection, chronic obstructive pulmonary disease, chronic bronchitis, restrictive lung diseases, respiratory failure, aortic coarctation, Cushing syndrome, pheochromocytoma, primary aldosteronism, renal parenchymal disease, renovascular hypertension, thyroid disorders, stroke, coronary heart disease, and those who could not provide a complete medical history. Detailed patient characteristics are presented in Table S5.

Cells and Tissues

Cell culture was carried out as previously described[1]. H5V endothelial cells (a kind gift from Prof. Yu Huang, Chinese University of Hong Kong, China), 293A cells, and murine erythroleukemia (MEL) cells were cultured in DMEM supplemented with 10% EV-free FBS (Exo-FBS™ EVs-depleted FBS, System Biosciences, USA), 100 IU/mL penicillin, and 100 μg/mL streptomycin. HUVECs were cultured in Endothelial Cell Medium (ScienCell Research Labs, USA) and 10% EV-free FBS. Mouse aortic rings (2 mm in length) were dissected in sterile PBS and incubated in DMEM (Gibco, USA) with or without EVs, Tempol (100 μM, Sigma, USA), Tiron (1 mM, Sigma, USA), DETCA (0.1 mM, Sigma, USA), or Oltipraz (100 μM, Sigma, USA). For intermittent hypoxia treatment, H5V cells, MEL cells, and aortic rings were exposed to an intermittent hypoxia generating system (Oxycycler model C42; Biospherix, Redfield, NY, USA) with the fractional ambient oxygen concentration cycling between 5%–21% per hour for the indicated time. For continuous hypoxia treatment, MEL cells were under the treatment of constant 5% oxygen concentration for the indicated time (Oxycycler model C42; Biospherix, Redfield, NY, USA). Prolyl hydroxylases (PHD) inhibitor dimethyloxalylglycine (DMOG, MCE, China) was used as HIF-1α stabilizer to mimic the hypoxia status. The transcriptional inhibitor actinomycin D (MCE, China) was used to inhibit the expression of endogenous miRNA in recipient endothelial cells.

Microarray Analysis

RNA from aortas or from HUVECs was isolated using Trizol (Thermo Fisher Scientific, USA). Total RNA was quantified using a NanoDrop ND-2000 (Thermo Scientifics, USA). RNA integrity was assessed using a Bioanalyzer 2100 (Agilent Technologies, Chandler, Arizona, USA). Sample labeling, microarray hybridization, and washing were performed according to the manufacturer's standard protocols. Genespring (version 13.1, Agilent Technologies, USA) was used to complete a basic analysis of the raw data. Raw data were normalized by the quantile algorithm. Probes possessing "P" flags in all conditions were selected for further analysis. Differentially expressed genes were then identified according to

fold change and P values calculated by the t-test. The threshold for up- and down-regulated genes was a fold change equal to or exceeding two, and a P value ≤ 0.05. Afterwards, GO analysis and KEGG analysis were applied to determine the roles of differentially expressed mRNAs. Hierarchical Clustering displayed distinguishable gene expression patterns among samples. Genes with overlapping differential expression under various conditions were identified using Venn diagrams.

EV Isolation from Serum and Red Blood Cells

S-EV isolation was carried out as previously described [1]. Blood from mice was clotted for 1 h at room temperature without anticoagulation, and centrifuged at 1,200×g for 10 min to obtain serum. Thereafter, the serum was centrifuged at 3,000×g for 10 min at 4°C. The supernatant was diluted in sterile PBS in a 1:1 ratio and centrifuged again at 14,000×g for 30 min followed by 2-h ultracentrifugation at 110,000×g (Beckman Coulter, Optima MAX-XP, USA) at 4°C. The EV pellet was washed in PBS, resuspended and passed through a 0.2-μm filter, and centrifuged at 110,000×g for 1 h at 4°C. The pellet fraction was then collected and resuspended in PBS or culture medium and used for subsequent functional wire myography studies (Danish Myo Technology 6200, Aarhus, Denmark) or purity evaluation by transmission electron microscopy using a NanoSight NS300 (Malvern Instruments, UK) or Western blotting.

E-EV isolation was carried out as previously described[2]. Red blood cells from normoxia- or CIH-treated mice were separated from EDTA-anticoagulated plasma by centrifugation at 3,000 rpm for 10 min. The upper plasma and leukocyte layer were removed by carefully pipetting and the fraction of red blood cells was then passed through a WBC Syringe Filter (Acrodisc PSF WBC Syringe Filter with Leukos, Pall, USA) to remove residual white blood cells. Approximately 350 μL of red blood cells were collected from the bottom of the microfuge tube. After washing in PBS, ~1×10^{10} red blood cells from CIH-treated mice were resuspended in EV-free 1640 culture medium and lightly shaken under normoxia or intermittent hypoxia conditions for 20 min. The collected RBC-derived conditioned media were centrifuged progressively at 300 × g for 10 min, 2,000 × g for 10 min, 12,000 × g for 45 min to remove RBCs, dead cells and cell debris. The supernatant was then passed through a 0.2µm microporous membrane filter (Millipore, MA, USA) and ultracentrifuged at 110,000 × g for 70 min (Beckman Coulter, CA, USA). The EV pellets were washed with PBS to exclude contaminating proteins followed by a second ultracentrifugation at 110,000 x g for 70 min. All the centrifugation steps were performed at 4°C. The EV pellet fraction was collected for qPCR, or resuspended in PBS or culture medium for subsequent cell culture and *in vivo* mouse treatments.

Transmission Electron Microscopy

Electron microscopy analysis of whole-mounted EVs was performed as previously described[1]. Briefly, S-EV pellets were fixed with 2% PFA and mounted onto grids. The grids were further fixed with 1% glutaraldehyde for 10 min and then negatively stained with methyl cellulose-UA for 10 min. After air drying, the grids were viewed using an FEI Tecnai electron microscope (FEI Tecnai, USA).

Western Blotting and Silver Staining

Protein samples prepared from C57BL/6 mouse aortas under the different conditions or H5V cells treated with agomiRs (Ribobio, Guangzhou, China) or S-EVs were electrophoresed through 10% SDS-poly-acrylamide gels, and transferred onto Immobilon-P polyvinylidene difluoride membranes (Millipore Corp., Bedford, MA, USA). Blots were incubated with antibodies and signals were detected using the ECL system (Pierce ECL Western Blotting Substrate, Thermo Fisher, Pittsburgh, USA). The primary antibodies used included anti-NRF2 (1:1000, Cell Signaling Technology, USA), anti-CAT (1:1000, Abcam), anti-β-ACTIN (1:5000, Abcam), anti-HIF-1α (1:1000, Abcam), and anti-GATA1 (1:1000, Abcam), anti-TSG101 (1:1000, System Bioscience), anti-CD9 (1:1000, System Bioscience), anti-CD81 (1:1000, System Bioscience), anti-CD63 (1:1000, System Bioscience), anti-HBA1 (1:1000, ABclonal), anti-Calnexin (1:1000, Cell Signaling Technology), anti-Alix (1:1000, System Bioscience). Silver stain was used as a loading control. Briefly, gels were fixed in 50% methanol plus 50% acetic acid for 20 min followed by sensitizing in 0.02% sodium thiosulfate for 20 min. Thereafter, gels were immersed in silver reaction buffer (0.1% silver nitrate in 0.08% formalin) for 20 min and developed with 2% sodium carbonate in 0.04% formalin.

Fluorescent Labeling of S-EVs and Confocal Microscopy

S-EVs were labeled with the fluorescent dye, PKH67 (Sigma, USA) as previously described[1]. The PKH67-labeled S-EV pellet was re-suspended in serum free-culture medium and incubated with mouse aortas. After incubation, aortas were cut open, washed, fixed, and visualized under confocal microscopy. Endothelial cell nuclei were stained blue with DAPI (excitation wavelength: 405 nm). EVs were stained green with PKH67 (excitation wavelength: 488 nm). Confocal microscopy images were captured with a Fluoview 1000 (FV1000, Olympus, Tokyo, Japan) and analyzed with Fluoview Version 1.5 (FV3000, Olympus).

Measurement of Superoxide Anion Production

Fluorimetric measurements were performed using the Fluoview FV3000 laser scanning confocal system (Olympus, Tokyo, Japan). H5V cells or aortas were incubated with DHE (5 μM in PBS containing $CaCl_2$, $MgCl_2$, and glucose) at 37°C for 10 min in the dark. After incubation, cells or tissues were washed three times in PBS. Images were taken of DHE fluorescence with an excitation wavelength of 510 nm and an emission wavelength of 590 nm. Auto-fluorescence of elastin was observed at an excitation wavelength of 488 nm and an emission wavelength of 520 nm.

Organ Culture of Aortic Rings with S-EVs or E-EVs

Mouse aortic rings (2 mm in length) were dissected in sterile PBS and incubated in DMEM (Gibco, USA) with or without S-EVs or E-EVs. The medium was prepared with S-EVs (from 1 mL mouse blood, ~100 μg) or with E-EVs (from 1×10^{10} red blood cells, ~50 μg) suspended in 1 mL DMEM supplemented with 10% EV-free FBS (Exo-FBS™ EVs-depleted FBS, System Biosciences), 100 IU/mL penicillin, and 100 μg/mL streptomycin. EV-free serum was used as a negative control for all experiments. After the incubation period, aortic rings were treated with DHE staining for superoxidative anion measurement or mounted for wire myography to measure isometric forces.

Vascular functional study

Vascular relaxation including endothelial-dependent relaxation, flow-mediated dilatation and endothelial-independent relaxation was performed as a previous report[1]. After mice were euthanized, thoracic aortae were removed and placed in oxygenated ice-cold PSS solution containing 130 mM NaCl, 4.7 mM KCl, 1.6 mM $CaCl_2$, 1.17 mM $MgSO_4$-H_2O, 14.9 mM $NaHCO_3$, 1.2 mM KH_2PO_4, 5.5 mM D-glucose, and 0.026 mM EDTA. Changes in the isometric tone of the aortic rings were recorded by wire myography (Danish Myo Technology 6200, Aarhus, Denmark). The rings were adjusted to an optimal baseline tension of 3 mN and allowed to equilibrate for one hour. Rings were rinsed in PSS solution before contraction with 60 mM KCl. EDR was measured in phenylephrine (3 μM) precontracted rings by recording concentration responses to cumulative addition of acetylcholine. One group of rings was incubated with ROS scavengers, Tiron (1 mM) and DETCA (0.1 mM) for 30 min before the assay. Endothelium-independent relaxation to sodium nitroprusside was performed in aortic rings denuded of endothelium using fine forceps. Acetylcholine, phenylephrine, and sodium nitroprusside were purchased from Sigma-Aldrich (Sigma Aldrich, USA).

For flow-mediated dilatation (FMD) assay, segments of second-order resistance mesenteric arteries were dissected in sterilized PBS and cultured in DMEM for 48 h. Thereafter, each vessel was cannulated between two glass cannulas onto pressure myograph. The vessel diameter was recorded using Zeiss Axiovert 40 microscope (model 110P) aided with video camera (Danish Myo Technology, Aarhus, Denmark). Phe (10 μM) was used to produce steady constriction in the artery stabilized at 80 mmHg intraluminal pressure; and FMD was triggered by pressure change that equals ~15 dynes/cm^2 shear stress. By the end of each experiment, perfusion solution was switched to a Ca^{2+}-free, EGTA (2 mM)-containing Krebs solution to induce maximum passive dilatation. FMD was presented as % of diameter changes: (flow-mediated dilatation- Phe tone)/(passive dilatation - Phe tone).

Electroporation of EVs

Electroporation of EVs was performed using a Gene Pulser Xcell electroporator (BioRad, USA). S-EVs or E-EVs were diluted in OptiMEM (Thermo Fisher Scientific) to a total volume of 100 μL and were electroporated with 400 pM scrambled negative control (NC) or indicated anti-miRs (Ribobio, Guangzhou, China) at 250 V. Aggregates of EVs formed during electroporation were dissociated by vigorous pipetting and immediately used to treat H5V cells or injected into mice *via* the tail vein.

RNA Extraction

RNA for pri-miRNA or mRNA detection from mouse aortas, mouse aortic endothelial cells, red blood cells, or H5V cells was isolated using Trizol (Thermo Fisher Scientific, USA). For isolation of microRNA from aortic endothelial cells, C57BL/6 mouse aortas were cleared of adventitial adipose tissue. The lumen was quickly flushed with 150 μL cell or tissue lysis buffer (mirVana miRNA Isolation Kit, Thermo Fisher Scientific, USA) three times using a 23 g syringe connected to a microfuge tube. The intimal miRNA was isolated from the eluate using an mirVana miRNA Isolation Kit (Thermo Fisher Scientific, USA) according to the manufacturer's protocol. The same kit was used to extract miRNA from mouse red blood cells. For isolation of miRNAs from EVs, 5 pg of the synthetic *Caenohabditis elegans* miRNA, cel-miR-39, was added to each sample as a spike-in control for purification efficiency.

miRNA and pri-miRNA Analysis

Isolated total RNAs were reverse-transcribed into complementary DNA by the M-MLV RT system (Thermo Fisher Scientific, USA). Real-time PCR for miRNAs was performed using Taqman Fast Universal PCR Master Mix (Applied Biosystems, USA). Reactions were carried out in Applied Biosystems 7900 instrument (Applied Biosystems, USA). Primer catalog numbers were: mmu482914 for mmu-miR-1a-3p; mmu480907 for mmu-miR-125b-5p; mmu478412 for mmu-miR-106b-5p; mmu482681 for mmu-miR-126a-3p; mmu481324 for mmu-miR-142a-5p; mmu483162 for mmu-let-7a-1-3p; mmu480919 for mmu-miR-132-3p mmu480947 for mmu-miR-150-5p; mmu478494 for mmu-miR-10b-5p; mmu480953 for mmu-miR-155-5p; mmu478384 for mmu-miR-27a-3p; and mmu482959 for mmu-miR-144-3p (TheromFisher, USA). Quantification of mRNAs and pri-miRNAs was performed using SYBR Green Supermix (Bio-Rad, USA). The primers used to amplify pri-miRNAs and mRNAs are shown in Table S6. Expression levels of mRNAs, pri-miRNAs, were normalized to control β-actin. Spike-in (cel-miR-39, miRNeasy Serum/Plasma Spike-In Control kit, QIAGEN, USA) as well as U6 was utilized as the reference to evaluate the levels of EV miRNAs. In EV-treated endothelial cells, U6 was used as the internal control for miR-144 expression. Real-time PCR was performed using an Applied Biosystems 7900 instrument (Applied Biosystems, USA).

Plasmid Construction and Luciferase Reporter Gene Assays

The 3'-UTR of mouse Nrf2 fragments was cloned into pMir luciferase reporter vector (Promega) at the *Xho*I and *Not*I sites. A 2,000-bp mouse miR-144 promoter fragment was cloned into MluI/XhoI sites of the reporter vector, pGL3-basic (miR-144-Luc), as wild type plasmid. For the mutant plasmid, GATA1 binding site GAAGATAAACA was mutated to CTTCTATTTGT. After confirmation by DNA sequencing, the purified plasmid was transiently transfected into 293A cells using Lipofectamine 2000 reagent at a ratio of 1 µL Lipofectamine 2000/1 µg of DNA according to the manufacturer's protocol (Thermo Fisher Scientific). The indicated reporter constructs (0.2 µg) were co-transfected with the indicated expression vectors and the internal control pRL-TK reporter (30 ng, Promega, USA). Luciferase activity was assessed using the Dual-Luciferase Reporter Assay System (Promega).

Lentiviral Treatment

C57BL/6 mouse aortas was transfected with lentiviruses (titer 10^9/mL) overexpressing *Nrf2* (from Vigene Biosciences, China) 293A cells or MEL cells were transfected with lentiviruses (titer 109/mL) overexpressing Hif-1α (Hif-1α OE) (a kind gift from Prof. Rui Chen, Capital Medical University, China) or Gata1 (from Vigene Biosciences, China), or Hif-1α shRNA or Gata1 shRNA to silence *Hif-1α* and *Gata1*, respectively (Santa Crus, USA).

Statistical Analysis

Statistical analysis is summarized in the figure legends. In most cases, the results represent the mean ± standard error of the mean (SEM) of n separate experiments. Concentration-response curves were compared by two-way analysis of variance (two-way ANOVA) followed by Bonferroni post-hoc test. Two-tailed Student's t-test was used when two groups were compared. One-way analysis of variance (ANOVA) was performed to determine whether there was a significant difference between more than two datasets, followed by Bonferroni's post hoc test. $P < 0.05$ indicates statistical difference between groups.

Supplementary Figures

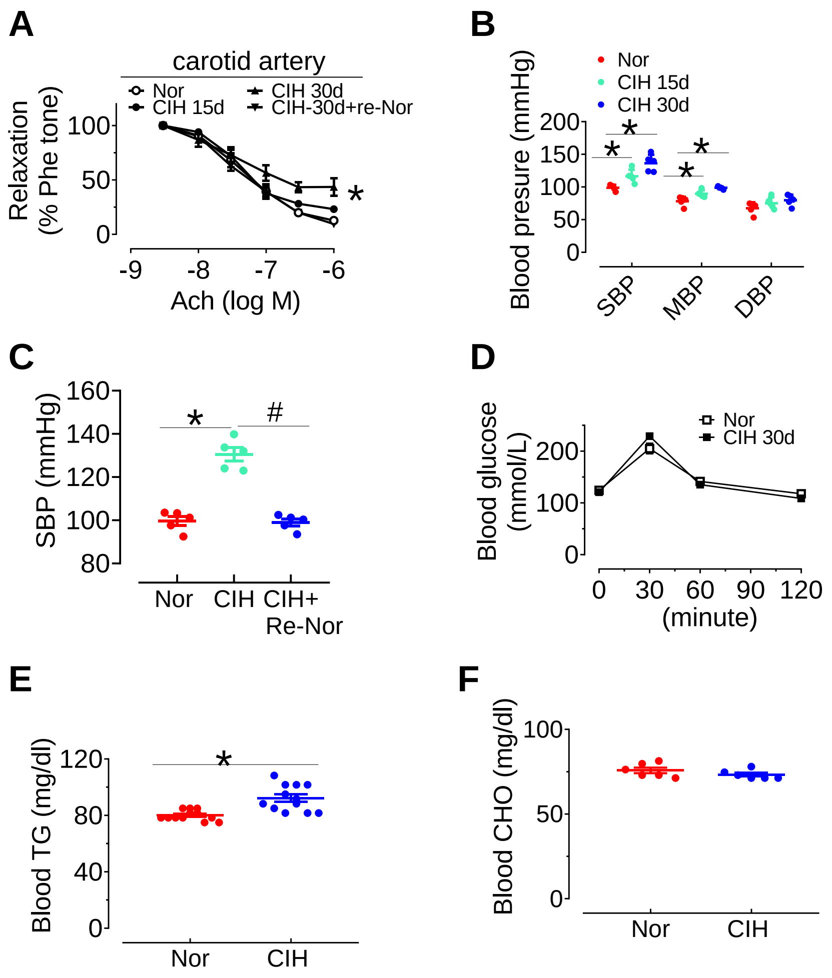

Figure S1. Endothelial function and physiological indices of C57BL/6 mice under normoxia and chronic intermittent hypoxia (CIH) conditions.

A, CIH treatment impaired endothelium-dependent relaxation in carotid arteries in a time-dependent manner. **B,** Levels of blood pressure in mice exposed to normoxia, CIH-15d and CIH-30d conditions. **C,** Levels of systolic blood pressure in mice exposed to normoxia, CIH-30d and CIH-30d plus15-day normal oxygen conditions. **D-F,** Levels of blood glucose (D), blood triglyceride (E) and blood cholesterol (F) in mice exposed to normoxia and CIH-30d conditions. TG, triglyceride; CHO, cholesterol. Results are the mean ± SEM (n = 4-12). $^{*}P < 0.05$ vs. Nor. $^{\#}P < 0.05$ vs. CIH. One-way ANOVA (B, C), two-way ANOVA (A) and two-tailed t test (E).

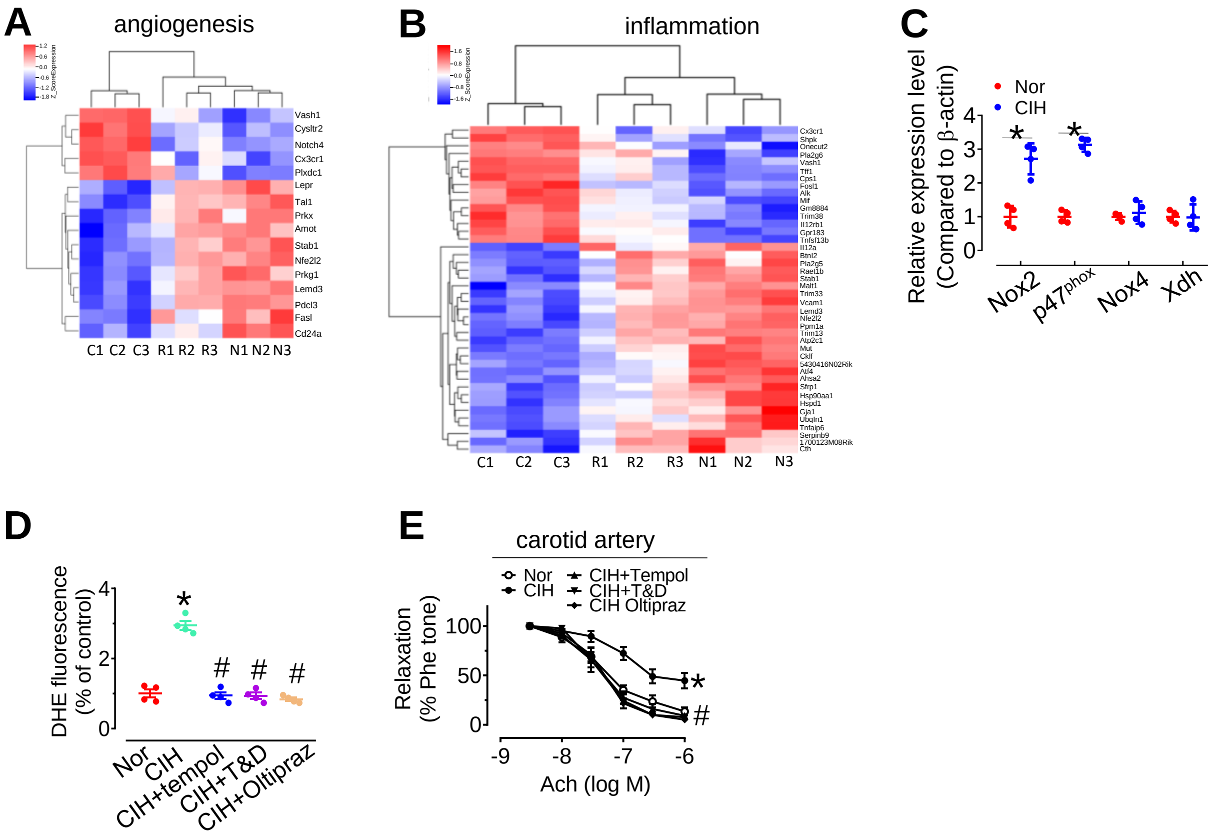

Figure S2. Endothelial function, endothelial superoxide anion production and profiles of differentially expressed genes in aortas from mice exposed to normoxia or chronic intermittent hypoxia (CIH) conditions.

A-B, Transcriptome microarray assays identified differentially expressed genes in aortas from mice treated with normoxia (N), CIH (C) or Re-Nor post-CIH (R). Kyoto Encyclopedia of Genes and Genomes (KEGG) and Gene ontology (GO) analysis predicted the functions of some of these genes to be associated with angiogenesis (A) or inflammation (B). **C,** mRNA expression of NOX subunits, *Nox2* and *p47^{phox}*, in mouse aortas was promoted by CIH treatment for 30 days. **D-E,** Thirty-minute pretreatment with ROS scavengers, Tempol (100 μM), or Tiron (1 mM) and DETCA (0.1 mM), or NRF2 agonist, Oltipraz (0.5 g/kg in saline, gavage once per day for 4 days before functional evaluation), blocked increased superoxide anion production in aortic endothelial cells (D) and reversed CIH-induced endothelial dysfunction in carotid aortas after CIH incubation for 30 days (E). Results are the mean ± SEM (n = 4). $^{*}P < 0.05$ vs. Nor. $^{\#}P < 0.05$ vs. CIH. One-way ANOVA (D), two-way ANOVA (E) and two-tailed t test (C).

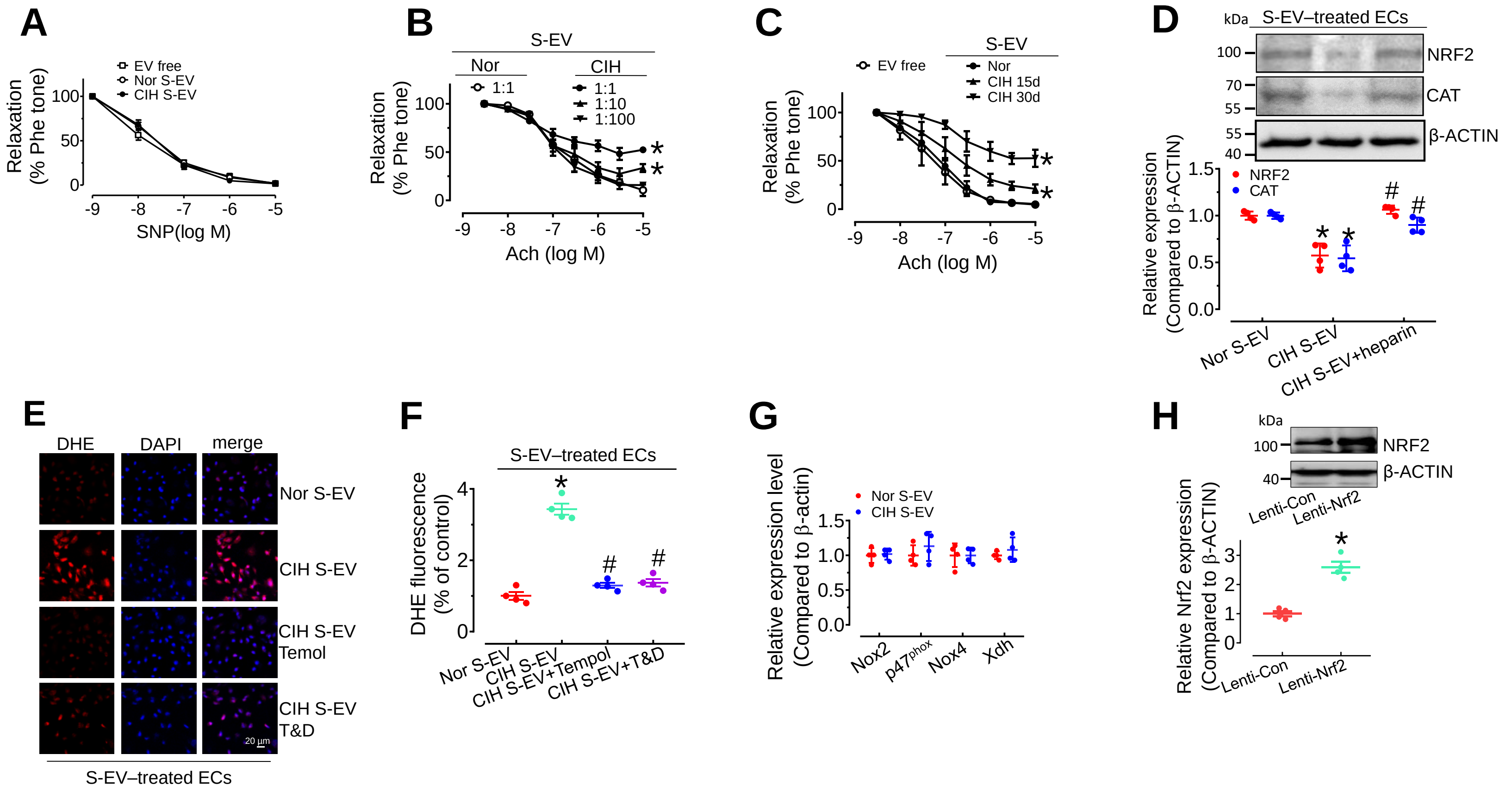

Figure S3. CIH S-EVs impair endothelial function, augment superoxide anion radical generation and decrease NRF2 expression in endothelial cells.

A, sodium nitroprusside-induced endothelium-independent relaxation of mouse aortas following serum extracellular vesicle (S-EV) treatment. **B,** Forty-eight-h treatment with CIH S-EVs impaired acetylcholine-induced endothelium-dependent relaxation (EDR) in mouse aortas in a dose-dependent manner. 1: n dilution means S-EVs from 1 mL blood were suspended in n mL S-EV-free medium. **C,** 30-day CIH S-EV treatment had a more severe adverse effect on endothelial function compared with 15-day CIH S-EV treatment. **D,** CIH S-EV treatment reduced the protein level of the antioxidant transcription factor, NRF2, and its downstream target, CAT, in H5V cells. The lower panels show the relative levels of NRF2 and CAT. **E-F,** CIH S-EV-incubation increased superoxide anion production in H5V cells, which was blocked by 30 min-pretreatment with ROS scavengers, Tempol (100 µM), or Tiron (1 mM) and DETCA (0.1 mM) (E) Summary of the dihydroethidium (DHE) fluorescence intensity (F). **G,** CIH S-EV did not change the mRNA levels of NOX subunits or *Xdh* in H5V cells. **H,** Lentivirus-mediated overexpression of *Nrf2* in mouse aortas after 48-h infection. Results are the mean ± SEM (n = 4). $^{*}P < 0.05$ vs. Nor S-EV. $^{\#}P < 0.05$ vs. CIH S-EV. One-way ANOVA (D, F, G) and two-way ANOVA (B, C).

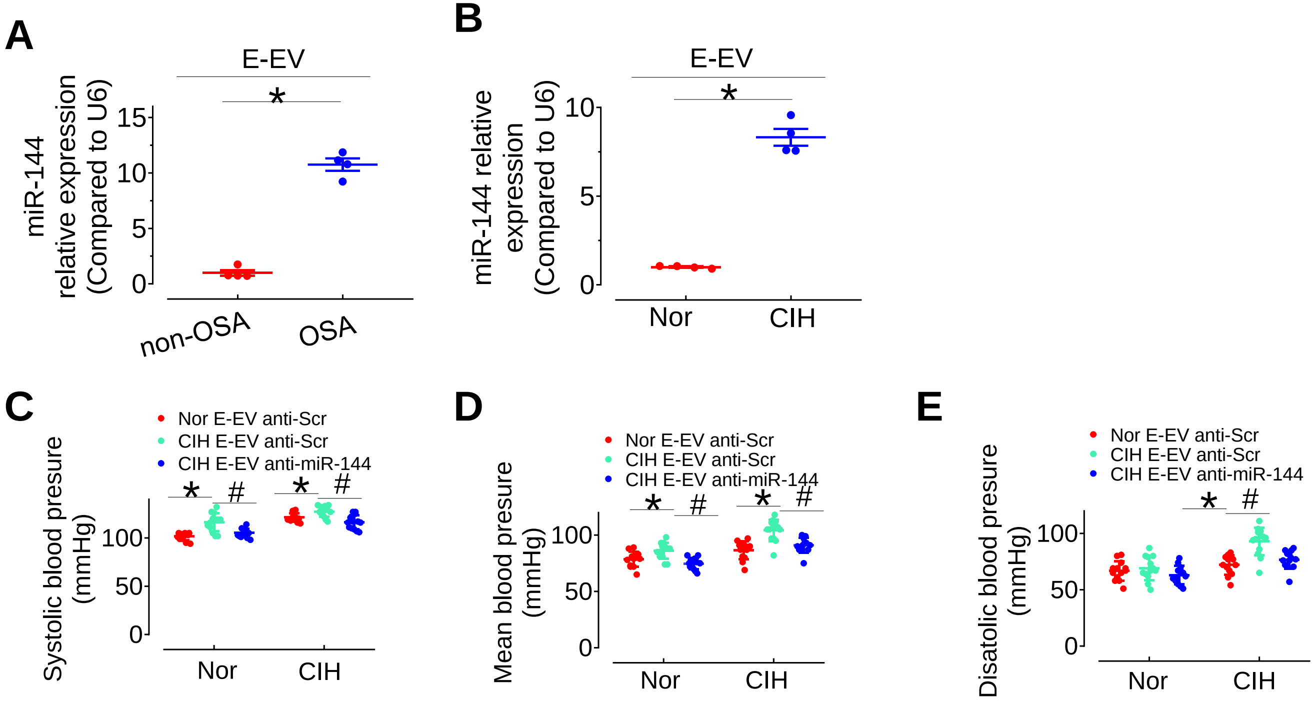

Figure S4. Blood pressure influenced by E-EVs and anti-miR-144-loaded CIH E-EVs.

A-B, qPCR determined the expression levels of miR-144 in E-EVs from OSA patients (A), CIH-exposed mice (B), and their control groups (U6 as the reference). **C-E,** The influence of Nor E-EVs, CIH E-EVs and anti-miR-144-loaded CIH S-EVs on systolic blood pressure (A), mean blood pressure (B) and diastolic blood pressure (C) under normoxia or chronic intermittent hypoxia. Results are the mean ± SEM (n = 4-13). $^{*}P < 0.05$ vs. OSA E-EV (A), or Nor E-EV (B), or Nor E-EV anti-Scr (C-E). $^{\#}P < 0.05$ vs. CIH E-EV anti-Scr. Two-tailed t test (A, B); One-way ANOVA (C-E).

Table S1. Differentially expressed proteins in aortas from CIH-treated mice.

No.	Target ID	Gene ID	Gene Symbol	Fold (IH vs.N)	Reg. (IH vs.N)	p Value (IH vs.N)	Fold (RNpIH vs.IH)	Reg. (RNpIH vs.IH)	p Value (RNpIH vs.IH)
1	AK084136	319388	Irx3os	-5.45	down	0.00	1.95	up	0.05
2	NM_010480	15519	Hsp90aa1	-4.23	down	0.00	1.85	up	0.04
3	NM_009786	12301	Cacybp	-4.16	down	0.01	1.94	up	0.04
4	NM_172391	268390	Ahsa2	-4.06	down	0.01	1.79	up	0.04
5	AK039015			-3.87	down	0.00	1.80	up	0.02
6	NM_013863	29810	Bag3	-3.62	down	0.00	1.83	up	0.02
7	AK018597	26359	Anxa10	-3.21	down	0.01	1.89	up	0.00
8	ENSMUST00000135943	66234	Msmo1	-3.18	down	0.00	1.55	up	0.03
9	NM_175692	319317	Snhg11	-3.17	down	0.01	1.93	up	0.05
10	NM_025819	66875	Swt1	-3.16	down	0.00	1.59	up	0.04
11	AK199574	70788	Klhl30	-3.13	down	0.00	1.28	up	0.02
12	BG080251	97177	Gm12670	-3.11	down	0.00	1.49	up	0.02
13	AK171865	105203	Fam208b	-3.06	down	0.00	1.77	up	0.03
14	AK087131			-3.04	down	0.00	1.89	up	0.02
15	NM_025844	66917	Chordc1	-3.01	down	0.01	1.72	up	0.05
16	NM_010177	14103	Fasl	-3.01	down	0.00	1.97	up	0.02
17	NM_028094	72094	Ugt2a3	-2.98	down	0.00	1.90	up	0.00
18	ENSMUST00000192142	108664	Atp6v1h	-2.93	down	0.00	1.85	up	0.02
19	ENSMUST00000172726			-2.90	down	0.00	1.78	up	0.04
20	AK039495	30056	Timm9	-2.89	down	0.00	1.85	up	0.00
21	NM_177225	320679	Samd12	-2.86	down	0.00	1.99	up	0.03
22	NM_177583	208117	Aph1b	-2.85	down	0.00	1.92	up	0.01
23	ENSMUST00000132911	269536	Tex10	-2.83	down	0.00	1.92	up	0.04
24	NM_009471	22247	Umps	-2.83	down	0.01	1.95	up	0.03
25	NM_001039710	67876	Coq10b	-2.82	down	0.00	1.82	up	0.04

26	ENSMUST00000051437			-2.81	down	0.02	1.97	up	0.03
27	NM_001122954	18784	Pla2g5	-2.77	down	0.01	1.93	up	0.04
28	XM_006527126	271564	Vps13a	-2.76	down	0.00	1.98	up	0.01
29	AV310571			-2.75	down	0.01	1.69	up	0.02
30	NM_001164286	408191	Gm5415	-2.75	down	0.00	1.56	up	0.00
31	ENSMUST00000069778	224224	Impg2	-2.73	down	0.00	1.75	up	0.00
32	XM_006535986	1E+08	LOC100862442	-2.72	down	0.00	1.67	up	0.03
33	NM_145521	227721	Ppapdc3	-2.70	down	0.01	1.90	up	0.00
34	NM_009551	22682	Zfand5	-2.69	down	0.00	1.74	up	0.01
35	NM_001256112	76824	Mtfr1l	-2.67	down	0.00	1.39	up	0.01
36	ENSMUST00000190232			-2.66	down	0.01	1.66	up	0.00
37	AK080258			-2.65	down	0.00	1.54	up	0.00
38	NM_009837	12464	Cct4	-2.64	down	0.00	1.74	up	0.01
39	NM_026674	68318	Aph1c	-2.63	down	0.00	1.75	up	0.02
40	XM_006534815	18764	Pkd2	-2.63	down	0.00	1.91	up	0.01
41	NM_177788	277978	Exoc3l	-2.59	down	0.00	1.35	up	0.01
42	NM_153786	215031	Vgll2	-2.58	down	0.02	1.80	up	0.02
43	ENSMUST00000145024	16847	Lepr	-2.57	down	0.00	1.87	up	0.04
44	NM_146118	227731	Slc25a25	-2.56	down	0.00	1.88	up	0.01
45	NM_027805	71474	Ppp6r2	-2.56	down	0.01	1.50	up	0.05
46	NM_181040	230649	Atpaf1	-2.56	down	0.00	1.78	up	0.00
47	ENSMUST00000099029			-2.56	down	0.00	1.65	up	0.00
48	ENSMUST00000152019	22083	Ctr9	-2.54	down	0.00	1.78	up	0.01
49	AK171523	407830	BC010981	-2.54	down	0.00	1.54	up	0.02
50	NAP111319-1			-2.54	down	0.00	1.52	up	0.01
51	AK047768	11472	Actn2	-2.52	down	0.01	1.70	up	0.00
52	NM_029295	75458	Cklf	-2.52	down	0.00	1.42	up	0.02
53	ENSMUST00000178218	216877	Dhx33	-2.52	down	0.00	1.71	up	0.02
54	NAP061415-1			-2.51	down	0.00	1.47	up	0.03
55	TC1636899			-2.51	down	0.00	1.84	up	0.03
56	NM_008351	16159	Il12a	-2.51	down	0.00	1.99	up	0.02

57	NM_001082485	77519	Zfp266	-2.50	down	0.00	1.97	up	0.02
58	NM_199062	331188	Zfp781	-2.50	down	0.00	1.80	up	0.00
59	ENSMUST00000160058			-2.50	down	0.01	1.63	up	0.03
60	ENSMUST00000162574			-2.49	down	0.00	1.54	up	0.02
61	NM_010477	15510	Hspd1	-2.48	down	0.01	1.67	up	0.03
62	NAP099731-001			-2.48	down	0.00	1.96	up	0.00
63	AK157456	329892	Gm830	-2.48	down	0.02	1.82	up	0.04
64	AK134128	14852	Gspt1	-2.48	down	0.00	1.54	up	0.02
65	NM_009543	22644	Rnf103	-2.47	down	0.00	1.59	up	0.03
66	AK172643	73130	Tmed5	-2.47	down	0.01	1.89	up	0.01
67	AI606402	73880	4930415C11Rik	-2.47	down	0.00	1.90	up	0.01
68	ENSMUST00000121742			-2.47	down	0.00	1.91	up	0.00
69	NM_001081152	244879	Npat	-2.46	down	0.01	1.92	up	0.03
70	ENSMUST00000030015	74753	5830415F09Rik	-2.46	down	0.00	1.55	up	0.01
71	NM_175250	76971	Sult2a8	-2.46	down	0.00	2.00	up	0.01
72	NM_177431	223838	Adamts20	-2.45	down	0.00	1.99	up	0.00
73	ENSMUST00000185704			-2.45	down	0.00	1.96	up	0.00
74	AK016918	71169	Nbas	-2.44	down	0.02	1.62	up	0.00
75	ENSMUST00000117199			-2.44	down	0.00	1.87	up	0.01
76	NM_025676	66634	Mcm8	-2.44	down	0.00	1.92	up	0.01
77	NM_010902	18024	Nfe2l2	-2.44	down	0.00	1.86	up	0.04
78	NM_009908	12764	Cmas	-2.44	down	0.00	1.46	up	0.01
79	NM_025875	60365	Rbm8a	-2.43	down	0.00	1.74	up	0.02
80	NM_145953	107869	Cth	-2.43	down	0.04	1.95	up	0.02
81	AK137479	52348	Vps37a	-2.43	down	0.00	1.46	up	0.04
82	NM_009549	22673	Zfp185	-2.42	down	0.01	1.51	up	0.01
83	NM_146189	233199	Mybpc2	-2.42	down	0.05	1.56	up	0.03
84	NM_133940	101358	Fbxl14	-2.41	down	0.00	1.77	up	0.01
85	NM_023773	75339	Mphosph8	-2.41	down	0.00	1.75	up	0.02
86	NM_001029977	214403	Gm4788	-2.41	down	0.00	1.90	up	0.00
87	NM_177356	239739	Lamp3	-2.40	down	0.00	1.68	up	0.01

88	ENSMUST00000119147			-2.40	down	0.00	1.66	up	0.00
89	NM_201369	381695	N4bp2l2	-2.40	down	0.00	1.64	up	0.00
90	NAP069232-1			-2.39	down	0.00	1.66	up	0.01
91	NM_008650	17850	Mut	-2.39	down	0.00	1.56	up	0.01
92	AK162903	72465	Zfp131	-2.39	down	0.00	1.61	up	0.04
93	NM_030072	665270	Plb1	-2.39	down	0.00	1.40	up	0.01
94	NM_028059	72020	Zfp654	-2.39	down	0.01	1.98	up	0.05
95	BC019993	21340	Taf1b	-2.38	down	0.00	1.33	up	0.01
96	NM_177304	320981	Enpp6	-2.38	down	0.00	1.92	up	0.04
97	NM_029851	110350	Dync2h1	-2.38	down	0.00	1.77	up	0.02
98	NM_009398	21930	Tnfaip6	-2.38	down	0.01	1.65	up	0.01
99	NM_007729	12814	Col11a1	-2.38	down	0.00	1.54	up	0.03
100	AK078446	1E+08	Gm14410	-2.38	down	0.00	1.85	up	0.01
101	NM_175308	100465	Mob3c	-2.38	down	0.00	1.59	up	0.04
102	NAP111303-1			-2.37	down	0.00	1.88	up	0.00
103	NM_178722	240186	Zfp438	-2.37	down	0.00	1.83	up	0.02
104	NM_010330	13723	Emb	-2.35	down	0.00	1.72	up	0.01
105	NM_029790	76894	Mettl15	-2.35	down	0.00	1.48	up	0.05
106	NM_029555	76263	Gstk1	-2.35	down	0.00	1.47	up	0.03
107	NM_001144855	68507	Ppfia4	-2.35	down	0.00	1.57	up	0.00
108	NM_011082	18703	Pigr	-2.35	down	0.01	1.57	up	0.02
109	NM_145368	209186	Acnat2	-2.35	down	0.00	1.66	up	0.02
110	NM_026578	68147	Gar1	-2.35	down	0.00	1.82	up	0.01
111	ENSMUST00000066241			-2.34	down	0.00	1.47	up	0.04
112	NAP005004-001			-2.33	down	0.00	1.81	up	0.01
113	NM_001287388	21349	Tal1	-2.33	down	0.00	1.88	up	0.00
114	NM_026637	110175	Ggct	-2.33	down	0.00	1.99	up	0.05
115	NM_028787	74150	Slc35f5	-2.33	down	0.01	1.99	up	0.01
116	NM_025421	66204	Acyp1	-2.33	down	0.00	1.80	up	0.01
117	ENSMUST00000188463	103268	Cep57l1	-2.33	down	0.01	1.94	up	0.01
118	NM_009826	12421	Rb1cc1	-2.33	down	0.00	1.62	up	0.02

119	AF425084	97848	Serpinb6c	-2.32	down	0.01	1.76	up	0.04
120	XM_006497255	329324	Syt14	-2.32	down	0.00	1.69	up	0.01
121	NM_053090	84652	Fam126a	-2.31	down	0.01	1.66	up	0.04
122	NM_178610	52705	Krr1	-2.31	down	0.00	1.56	up	0.01
123	NM_001081254	545136	Fam186b	-2.31	down	0.00	1.51	up	0.03
124	NM_021421	52477	Angel2	-2.31	down	0.00	1.65	up	0.03
125	NM_001114332	72472	Slc16a10	-2.31	down	0.01	1.96	up	0.01
126	ENSMUST00000142299			-2.31	down	0.00	1.69	up	0.01
127	NM_009017	19369	Raet1b	-2.31	down	0.00	1.70	up	0.03
128	NM_134072	105387	Akr1c14	-2.31	down	0.00	1.72	up	0.02
129	NM_001164493	208439	Klhl29	-2.31	down	0.01	1.81	up	0.02
130	ENSMUST00000122407			-2.30	down	0.00	1.83	up	0.00
131	DW711327			-2.30	down	0.00	1.38	up	0.01
132	AK016293	114670	4930573O21Rik	-2.30	down	0.01	1.64	up	0.01
133	NM_025513	66362	Exosc3	-2.29	down	0.00	1.85	up	0.00
134	XM_006514760	544763	Hbq1b	-2.29	down	0.00	1.79	up	0.00
135	NM_013642	19252	Dusp1	-2.29	down	0.01	1.57	up	0.03
136	NM_011721	22427	Wrn	-2.29	down	0.00	1.72	up	0.00
137	NM_025903	15983	Ifrd2	-2.29	down	0.00	1.74	up	0.01
138	NM_197943	97761	Sgsm2	-2.29	down	0.00	1.71	up	0.01
139	NM_025483	66315	Senp7	-2.29	down	0.03	1.77	up	0.04
140	NM_001128151	330409	Cecr2	-2.28	down	0.00	1.64	up	0.00
141	NM_009152	20346	Sema3a	-2.28	down	0.01	1.78	up	0.00
142	NM_146532	258525	Olfr1170	-2.28	down	0.00	1.45	up	0.03
143	NM_053137	93883	Pcdhb12	-2.27	down	0.00	1.69	up	0.00
144	ENSMUST00000139897	22608	Ybx1	-2.27	down	0.00	1.99	up	0.00
145	NM_001013833	19091	Prkg1	-2.27	down	0.00	1.80	up	0.01
146	NM_028817	74205	Acsl3	-2.26	down	0.00	1.91	up	0.01
147	NM_008892	18968	Pola1	-2.26	down	0.00	1.85	up	0.01
148	NAP094867-001			-2.26	down	0.00	1.66	up	0.02
149	ENSMUST00000199146			-2.26	down	0.00	1.66	up	0.00

150	NM_028011	71943	Tom1l1	-2.26	down	0.00	1.99	up	0.00
151	NM_026904	68999	Anapc10	-2.25	down	0.00	1.65	up	0.02
152	BC027541	320586	A630089N07Rik	-2.25	down	0.00	1.69	up	0.01
153	NM_001161765	14263	Fmo5	-2.25	down	0.01	1.64	up	0.03
154	NM_138672	192187	Stab1	-2.25	down	0.00	1.61	up	0.04
155	NM_146009	216274	Cep290	-2.25	down	0.02	1.61	up	0.02
156	NM_009838	12466	Cct6a	-2.25	down	0.00	1.63	up	0.01
157	NM_133702	68979	Nol11	-2.24	down	0.00	1.57	up	0.00
158	NM_001290350	22696	Zfp37	-2.24	down	0.00	1.55	up	0.04
159	NM_028108	72117	Naa50	-2.24	down	0.00	1.58	up	0.02
160	NM_001102458	54375	Azin1	-2.24	down	0.01	1.54	up	0.03
161	NM_009846	12484	Cd24a	-2.24	down	0.00	1.38	up	0.04
162	NM_025722	66714	4921524J17Rik	-2.24	down	0.00	1.84	up	0.03
163	NM_001081021	338354	Zfp780b	-2.24	down	0.00	1.74	up	0.04
164	ENSMUST00000125228	22718	Zfp60	-2.24	down	0.01	1.43	up	0.03
165	NM_008467	16649	Kpna4	-2.23	down	0.00	1.92	up	0.01
166	NM_173734	211499	Tmem87a	-2.23	down	0.01	1.83	up	0.00
167	ENSMUST00000151080	18667	Pgr	-2.23	down	0.02	1.96	up	0.04
168	NM_008690	18037	Nfkbie	-2.23	down	0.00	1.81	up	0.01
169	NM_175357	58799	Crbn	-2.23	down	0.00	1.79	up	0.02
170	NM_029068	74718	Snx16	-2.23	down	0.00	1.38	up	0.03
171	NM_201529	380928	Lmo7	-2.23	down	0.00	1.45	up	0.04
172	ENSMUST00000151163	22343	Lin7c	-2.23	down	0.00	1.54	up	0.02
173	XM_006530046	1E+08	Gm14409	-2.23	down	0.00	1.58	up	0.01
174	NM_016746	51813	Ccnc	-2.22	down	0.00	1.65	up	0.00
175	NM_008379	16211	Kpnb1	-2.22	down	0.00	1.86	up	0.01
176	NM_001033851	66871	Cpne8	-2.22	down	0.00	1.58	up	0.00
177	CO430613			-2.22	down	0.00	1.99	up	0.01
178	NM_053075	19744	Rheb	-2.22	down	0.00	1.96	up	0.00
179	NM_028116	72135	Pygo1	-2.22	down	0.01	1.90	up	0.03
180	NM_028924	74413	Tc2n	-2.22	down	0.00	1.84	up	0.01

181	NM_029564	76281	Tax1bp3	-2.22	down	0.00	1.60	up	0.01
182	NM_001301859	22200	Uba3	-2.22	down	0.00	1.77	up	0.00
183	ENSMUST00000041638	98488	Gtf3c3	-2.22	down	0.00	1.89	up	0.00
184	NM_025820	66877	Crnkl1	-2.22	down	0.00	1.87	up	0.02
185	NM_029749	76800	Usp42	-2.21	down	0.00	1.70	up	0.02
186	NM_001205101	433801	Gm13212	-2.21	down	0.00	1.89	up	0.01
187	NM_026554	68092	Ncbp2	-2.21	down	0.00	1.87	up	0.01
188	NM_001005358	449000	Zfp960	-2.21	down	0.00	1.81	up	0.03
189	NM_011919	26356	Ing1	-2.21	down	0.00	1.80	up	0.01
190	NM_176987	319719	Simc1	-2.21	down	0.00	1.67	up	0.02
191	NM_001190356	225058	Gm4832	-2.21	down	0.00	1.40	up	0.02
192	NM_001142952	74645	Fam46c	-2.21	down	0.00	1.62	up	0.02
193	NM_181517	233726	Ipo7	-2.21	down	0.00	1.93	up	0.01
194	NM_001033156	70611	Fbxo33	-2.21	down	0.00	1.82	up	0.02
195	XM_006543774	625281	Gm6570	-2.21	down	0.00	1.52	up	0.03
196	XM_006531843		1700055N04Rik	-2.20	down	0.00	1.97	up	0.00
197	NM_139232	224014	Fgd4	-2.20	down	0.00	1.85	up	0.01
198	NM_026396	67832	Brix1	-2.20	down	0.01	1.87	up	0.04
199	NM_177588	208967	Thnsl1	-2.20	down	0.00	1.74	up	0.01
200	NM_173446	270028	Fam155a	-2.20	down	0.00	1.88	up	0.00
201	NM_026850	68833	Pdcl3	-2.19	down	0.00	1.75	up	0.01
202	NM_175394	60532	Wtap	-2.19	down	0.00	1.70	up	0.01
203	NM_001014397	433804	Gm13154	-2.19	down	0.00	1.80	up	0.03
204	NM_145492	225207	Zfp521	-2.19	down	0.01	1.75	up	0.02
205	NM_146016	237711	Eml6	-2.19	down	0.00	1.58	up	0.01
206	NM_145943	208768	Sde2	-2.19	down	0.00	1.83	up	0.02
207	NM_144811	52609	Cbx7	-2.18	down	0.01	1.89	up	0.05
208	NM_011496	20877	Aurkb	-2.18	down	0.00	1.89	up	0.04
209	XM_011244594	1E+08	Gm3325	-2.18	down	0.02	1.60	up	0.01
210	NM_011693	22329	Vcam1	-2.18	down	0.00	1.54	up	0.01
211	NM_009770	12228	Btg3	-2.17	down	0.00	1.79	up	0.01

212	NM_001012638	497652	Acd	-2.17	down	0.00	1.96	up	0.00
213	NM_026348	67733	Itgb3bp	-2.17	down	0.00	1.99	up	0.01
214	NM_001039184	74081	Cep350	-2.17	down	0.00	1.69	up	0.01
215	NM_021273	12709	Ckb	-2.17	down	0.00	1.40	up	0.02
216	NM_001142963	1E+08	Gm10778	-2.17	down	0.00	1.75	up	0.00
217	ENSMUST00000119319			-2.17	down	0.00	1.88	up	0.01
218	FJ422280	1E+08	Gm17501	-2.17	down	0.01	1.91	up	0.03
219	NM_010016	13136	Cd55	-2.17	down	0.00	1.57	up	0.01
220	NM_019727	56440	Snx1	-2.17	down	0.00	1.74	up	0.01
221	NM_001256055	1E+08	Eif3j2	-2.17	down	0.01	1.63	up	0.01
222	NM_007874	13476	Reep5	-2.17	down	0.00	1.41	up	0.04
223	NM_025992	67138	Herc6	-2.16	down	0.00	1.57	up	0.01
224	NM_007597	12330	Canx	-2.16	down	0.00	1.44	up	0.02
225	XM_006503340	67770	Caap1	-2.16	down	0.01	1.51	up	0.01
226	ENSMUST00000120745			-2.16	down	0.00	1.59	up	0.01
227	NM_019570	56210	Rev1	-2.16	down	0.00	1.94	up	0.03
228	NM_011129	18952	43712	-2.16	down	0.00	1.39	up	0.01
229	NM_025825	66884	Appbp2	-2.16	down	0.00	1.58	up	0.01
230	NM_023233	66597	Trim13	-2.16	down	0.00	1.69	up	0.01
231	NM_026654	68276	Toe1	-2.16	down	0.00	1.93	up	0.01
232	NM_008303	15528	Hspe1	-2.16	down	0.02	1.69	up	0.04
233	NM_020283	26877	B3galt1	-2.16	down	0.00	1.65	up	0.00
234	ENSMUST00000120090			-2.15	down	0.00	1.88	up	0.00
235	ENSMUST00000182000			-2.15	down	0.00	1.69	up	0.00
236	ENSMUST00000120408			-2.15	down	0.00	1.81	up	0.05
237	XM_011251171	732521	Gt(pU21)140Imeg	-2.15	down	0.00	1.79	up	0.01
238	NM_031165	15481	Hspa8	-2.15	down	0.01	1.75	up	0.05
239	NM_001015099	217558	G2e3	-2.15	down	0.01	1.56	up	0.03
240	NM_011976	26456	Sema4g	-2.14	down	0.01	1.86	up	0.03
241	NM_175226	75841	Rnf139	-2.14	down	0.00	1.94	up	0.01
242	ENSMUST00000117894			-2.14	down	0.00	1.85	up	0.00

243	NM_009261	20744	Strbp	-2.14	down	0.00	1.75	up	0.00
244	NM_001081688	432478	Tmprss9	-2.14	down	0.00	1.63	up	0.00
245	AK214641			-2.14	down	0.04	1.99	up	0.03
246	NM_010914	18045	Nfyb	-2.14	down	0.00	1.23	up	0.04
247	ENSMUST00000119165			-2.14	down	0.00	1.80	up	0.01
248	NAP095021-001			-2.14	down	0.00	1.71	up	0.02
249	NM_153780	72139	2610044O15Rik8	-2.14	down	0.01	1.87	up	0.02
250	NM_173746	208820	Triqk	-2.14	down	0.00	1.83	up	0.03
251	NM_133231	170767	Rfxap	-2.14	down	0.00	1.66	up	0.01
252	NM_001001798	320940	Atp11c	-2.13	down	0.01	1.88	up	0.02
253	NM_008505	16909	Lmo2	-2.13	down	0.01	1.82	up	0.04
254	NM_024438	68082	Dusp19	-2.13	down	0.00	1.85	up	0.02
255	NM_001081222	77805	Esco1	-2.13	down	0.00	1.70	up	0.01
256	NM_145934	106766	Stap2	-2.13	down	0.01	1.65	up	0.01
257	NM_027326	70122	Mllt3	-2.13	down	0.00	1.78	up	0.00
258	NM_025359	66109	Tspan13	-2.13	down	0.00	1.59	up	0.01
259	NM_027182	69716	Trip13	-2.13	down	0.00	1.75	up	0.01
260	NM_201244	54161	Copg1	-2.13	down	0.02	1.84	up	0.03
261	CA579447			-2.13	down	0.00	1.58	up	0.04
262	ENSMUST00000161974			-2.13	down	0.00	1.73	up	0.02
263	NM_030081	78287	Rbsn	-2.12	down	0.00	1.94	up	0.02
264	NM_080456	121022	Mrps6	-2.12	down	0.02	1.68	up	0.03
265	AK172004	494504	Apcdd1	-2.12	down	0.00	1.85	up	0.03
266	ENSMUST00000188847	213109	Phf3	-2.12	down	0.00	1.52	up	0.03
267	NM_147113	259117	Olfr560	-2.12	down	0.00	1.65	up	0.02
268	AK148702			-2.12	down	0.01	1.78	up	0.01
269	NM_028932	74427	Eaf1	-2.12	down	0.00	1.59	up	0.03
270	NM_008452	16598	Klf2	-2.12	down	0.00	1.40	up	0.01
271	NM_009952	12912	Creb1	-2.12	down	0.00	1.36	up	0.04
272	XM_006534635	1E+08	LOC102632383	-2.12	down	0.00	1.34	up	0.01
273	NM_007857	13363	Dhh	-2.12	down	0.00	1.34	up	0.01

274	NM_011277	19821	Rnf2	-2.12	down	0.01	1.68	up	0.04
275	NM_001025373	74670	Zfp943	-2.12	down	0.00	1.83	up	0.00
276	ENSMUST00000153501			-2.12	down	0.01	1.93	up	0.01
277	NM_001039521	106298	Rrn3	-2.12	down	0.00	1.70	up	0.00
278	NM_133167	170736	Parvb	-2.11	down	0.04	1.78	up	0.03
279	NM_019865	19982	Rpl36a	-2.11	down	0.00	1.46	up	0.02
280	AK155848	70449	2610209C05Rik	-2.11	down	0.00	1.48	up	0.01
281	ENSMUST00000087143	66235	Eif1ax	-2.11	down	0.00	1.54	up	0.01
282	NM_019721	56335	Mettl3	-2.11	down	0.00	1.70	up	0.03
283	NM_026054	67246	2810474O19Rik	-2.11	down	0.00	1.78	up	0.01
284	ENSMUST00000118547			-2.11	down	0.00	1.61	up	0.02
285	NM_028110	72121	Dennd2d	-2.11	down	0.01	1.73	up	0.03
286	NAP115053-1			-2.11	down	0.00	1.48	up	0.03
287	NM_007604	12343	Capza2	-2.11	down	0.00	1.68	up	0.00
288	NM_013834	20377	Sfrp1	-2.11	down	0.00	1.44	up	0.03
289	NM_173181	67306	Zc2hc1a	-2.11	down	0.00	1.95	up	0.02
290	NAP059691-1			-2.11	down	0.00	1.70	up	0.01
291	AF369981	19042	Ppm1a	-2.11	down	0.00	1.64	up	0.03
292	AK162394	21391	Tbxas1	-2.10	down	0.04	1.46	up	0.03
293	NM_001199956	408192	Gm9839	-2.10	down	0.00	1.40	up	0.02
294	XM_006540799	22215	Ube3a	-2.10	down	0.00	1.71	up	0.02
295	NM_001024846	22720	Zfp62	-2.10	down	0.00	1.51	up	0.00
296	NM_010849	17869	Myc	-2.10	down	0.01	1.79	up	0.00
297	XM_006543383	328825	Gm5093	-2.10	down	0.00	1.67	up	0.01
298	NM_001271016	66799	Ube2w	-2.10	down	0.00	1.60	up	0.01
299	NM_145969	211922	Dennd6a	-2.10	down	0.00	1.44	up	0.04
300	NM_146249	240120	Zfp119b	-2.10	down	0.00	1.52	up	0.01
301	NM_026479	67966	Zcchc10	-2.10	down	0.00	1.65	up	0.02
302	NM_029271	75398	Mrpl32	-2.10	down	0.00	1.87	up	0.03
303	NM_025901	67005	Polr3k	-2.10	down	0.00	1.85	up	0.00
304	NM_176962	319615	Zfp944	-2.10	down	0.00	1.74	up	0.01

305	ENSMUST00000134741	224902	Safb2	-2.09	down	0.00	1.65	up	0.01
306	NM_023210	66471	Anp32e	-2.09	down	0.00	1.98	up	0.00
307	NM_029434	75782	Lca5	-2.09	down	0.00	2.00	up	0.01
308	NM_001164593	239618	Pdzrn4	-2.09	down	0.03	1.75	up	0.03
309	ENSMUST00000080175			-2.09	down	0.00	1.79	up	0.02
310	NM_021326	57782	Rbak	-2.09	down	0.00	1.82	up	0.04
311	NM_001081420	230234	Fam206a	-2.09	down	0.00	1.82	up	0.00
312	NM_001290548	320267	Fubp3	-2.09	down	0.00	1.82	up	0.00
313	NM_016957	15331	Hmgn2	-2.09	down	0.01	1.87	up	0.02
314	NM_009716	11911	Atf4	-2.09	down	0.00	1.30	up	0.01
315	NM_028487	73274	Gpbp1	-2.09	down	0.00	1.68	up	0.00
316	NM_178379	70383	Cox10	-2.09	down	0.00	1.76	up	0.01
317	AK201505	104175	Sbk1	-2.09	down	0.00	1.82	up	0.01
318	AK132208	20042	Rps12	-2.09	down	0.00	1.55	up	0.02
319	XM_006529444	226442	Zfp281	-2.09	down	0.01	1.30	up	0.00
320	NM_001177525	1E+08	EU599041	-2.09	down	0.01	1.96	up	0.01
321	NM_001001932	216238	Eea1	-2.08	down	0.00	1.75	up	0.01
322	NM_009087	20018	Polr1d	-2.08	down	0.00	1.68	up	0.00
323	XM_006515438	11855	Arhgap5	-2.08	down	0.00	1.43	up	0.01
324	ENSMUST00000093546			-2.08	down	0.00	1.46	up	0.02
325	NM_178069	224092	Lsg1	-2.08	down	0.01	1.84	up	0.01
326	NM_172586	218100	Zfp322a	-2.08	down	0.00	1.49	up	0.03
327	NM_001164676	381067	Zfp229	-2.08	down	0.01	1.70	up	0.01
328	NM_010288	14609	Gja1	-2.08	down	0.02	1.55	up	0.01
329	NM_177715	239217	Kctd12	-2.08	down	0.01	1.85	up	0.05
330	NM_029570	76295	Atp11b	-2.08	down	0.00	1.39	up	0.02
331	NM_009946	12890	Cplx2	-2.08	down	0.01	1.80	up	0.01
332	ENSMUST00000119605			-2.08	down	0.00	1.83	up	0.00
333	AK034948	67712	Slc25a37	-2.08	down	0.02	1.84	up	0.04
334	XM_006522805	433003	Gm5481	-2.08	down	0.00	1.34	up	0.03
335	NM_133774	170459	Stard4	-2.08	down	0.01	1.65	up	0.04

336	ENSMUST00000118023			-2.07	down	0.01	1.75	up	0.00
337	NM_172833	240354	Malt1	-2.07	down	0.00	1.81	up	0.02
338	NM_009881	12593	Cdyl	-2.07	down	0.01	1.76	up	0.02
339	NM_001033348	241694	Ralgapa2	-2.07	down	0.00	1.42	up	0.00
340	NM_001079830	94093	Trim33	-2.07	down	0.00	1.53	up	0.01
341	AK040904	1E+08	Gm26510	-2.07	down	0.00	1.37	up	0.04
342	NM_025970	67106	Zbtb8os	-2.07	down	0.00	1.73	up	0.01
343	AK040596			-2.07	down	0.00	1.28	up	0.04
344	ENSMUST00000178086			-2.07	down	0.00	1.69	up	0.01
345	NM_025885	381379	Med19	-2.07	down	0.00	1.85	up	0.01
346	NM_080558	70599	Ssfa2	-2.07	down	0.00	1.81	up	0.02
347	NM_011643	22063	Trpc1	-2.07	down	0.01	1.56	up	0.02
348	XM_006495473	19888	Rp1	-2.07	down	0.03	1.65	up	0.05
349	AK048998	382421	Gm5176	-2.07	down	0.00	1.38	up	0.05
350	NM_028334	69736	Nup37	-2.07	down	0.00	1.84	up	0.01
351	NM_024242	71340	Riok1	-2.06	down	0.00	1.70	up	0.01
352	NM_001111320	15926	Idh1	-2.06	down	0.00	1.98	up	0.00
353	NM_001025572	106585	Ankrd12	-2.06	down	0.00	1.70	up	0.05
354	NM_144787	76804	Kdm4c	-2.06	down	0.00	1.64	up	0.01
355	NM_001289781	78697	Pus7	-2.06	down	0.04	1.51	up	0.04
356	NM_007638	12468	Cct7	-2.06	down	0.00	1.43	up	0.02
357	NM_194269	378462	Morn2	-2.06	down	0.01	1.91	up	0.00
358	NM_212457	406217	Bex4	-2.06	down	0.00	1.75	up	0.03
359	NM_133818	98404	AI597479	-2.06	down	0.00	1.88	up	0.01
360	NM_009831	12450	Ccng1	-2.06	down	0.00	1.69	up	0.02
361	NM_145455	218490	Btf3	-2.06	down	0.00	1.68	up	0.01
362	NM_153319	27494	Amot	-2.06	down	0.01	1.80	up	0.03
363	NM_001199350	619547	Rpl34-ps1	-2.06	down	0.00	1.56	up	0.03
364	NM_026536	68055	Atp5s	-2.05	down	0.00	1.80	up	0.00
365	NAP113774-1			-2.05	down	0.00	1.71	up	0.01
366	NM_177712	238692	Zfp874a	-2.05	down	0.01	1.73	up	0.02

367	NM_172865	242362	Manea	-2.05	down	0.00	1.82	up	0.00
368	NM_001170454	231151	Tada2b	-2.05	down	0.00	1.70	up	0.01
369	ENSMUST00000121467			-2.05	down	0.00	1.82	up	0.00
370	NM_001170849	192196	Luc7l2	-2.05	down	0.00	1.54	up	0.02
371	NM_027617	70951	Spata1	-2.05	down	0.00	1.78	up	0.00
372	NM_178766	319653	Slc25a40	-2.05	down	0.00	1.67	up	0.01
373	NM_001024910	103080	43718	-2.05	down	0.00	1.70	up	0.00
374	ENSMUST00000141874	20402	Zfp106	-2.05	down	0.01	1.84	up	0.03
375	NM_009256	20723	Serpinb9	-2.05	down	0.01	1.73	up	0.02
376	NM_029610	73919	Lyrm1	-2.05	down	0.00	1.79	up	0.00
377	NM_177039	319942	A530016L24Rik	-2.05	down	0.00	1.23	up	0.01
378	NAP111293-1			-2.05	down	0.00	1.89	up	0.00
379	NM_028677	66101	Ppih	-2.05	down	0.01	1.85	up	0.04
380	NM_001289658	76223	Agbl3	-2.05	down	0.00	1.93	up	0.02
381	NM_028990	101118	Tmem168	-2.05	down	0.00	1.75	up	0.01
382	NM_031391	83602	Gtf2a1	-2.05	down	0.00	1.57	up	0.02
383	NM_001081090	66580	Esf1	-2.05	down	0.00	1.61	up	0.00
384	NM_133740	71974	Prmt3	-2.05	down	0.00	1.91	up	0.03
385	NM_007552	12151	Bmi1	-2.04	down	0.00	1.80	up	0.02
386	NM_027203	69757	Leng1	-2.04	down	0.00	1.79	up	0.01
387	NM_133895	100561	Slc15a4	-2.04	down	0.00	1.30	up	0.03
388	NM_028319	72672	Zfp518a	-2.04	down	0.00	1.37	up	0.05
389	XM_006527909	19108	Prkx	-2.04	down	0.01	1.98	up	0.00
390	XM_006540002	26436	Psg16	-2.04	down	0.00	1.82	up	0.03
391	NM_001001176	407786	Taf9b	-2.04	down	0.00	1.78	up	0.03
392	NM_013843	24132	Zfp53	-2.04	down	0.00	1.93	up	0.00
393	TC1602431			-2.04	down	0.00	1.42	up	0.01
394	NM_020595	57329	Otor	-2.04	down	0.00	1.42	up	0.02
395	XM_006505459	12444	Ccnd2	-2.04	down	0.00	1.69	up	0.02
396	NM_007890	13548	Dyrk1a	-2.04	down	0.00	1.59	up	0.01
397	NM_008186	14884	Gtf2h1	-2.04	down	0.01	1.97	up	0.02

398	NM_152234	56085	Ubqln1	-2.04	down	0.00	1.34	up	0.01
399	XM_006515301	1E+08	Gm29738	-2.03	down	0.01	1.56	up	0.02
400	NM_027807	75717	Cul5	-2.03	down	0.00	1.64	up	0.01
401	NM_026046	67230	Zfp329	-2.03	down	0.00	1.19	up	0.03
402	NM_001080706	107182	Btaf1	-2.03	down	0.01	1.59	up	0.04
403	NM_173761	228994	Ythdf1	-2.03	down	0.00	1.54	up	0.01
404	ENSMUST00000147830	17763	Mtcp1	-2.03	down	0.02	1.42	up	0.02
405	NM_079835	547431	Btnl2	-2.03	down	0.01	1.97	up	0.01
406	NM_025776	66810	Rbm22	-2.03	down	0.00	1.90	up	0.03
407	NM_053155	94040	Clmn	-2.03	down	0.04	1.75	up	0.00
408	NM_001122675	227449	Zcchc2	-2.03	down	0.00	1.59	up	0.05
409	NM_013778	27384	Akr1c13	-2.03	down	0.02	1.86	up	0.01
410	NM_001162921	244871	Zc3h12c	-2.03	down	0.00	1.99	up	0.00
411	NM_172645	226551	Suco	-2.03	down	0.00	1.88	up	0.01
412	NM_011794	23827	Bpnt1	-2.03	down	0.00	1.61	up	0.03
413	NM_028626	73724	Mcee	-2.03	down	0.01	1.88	up	0.02
414	ENSMUST00000119137			-2.03	down	0.00	1.65	up	0.00
415	NM_029330	75540	Fpgt	-2.03	down	0.00	1.70	up	0.03
416	NM_054087	116914	Slc19a2	-2.03	down	0.00	1.54	up	0.03
417	NM_029505	64933	Ap3m2	-2.02	down	0.01	1.83	up	0.01
418	NM_025564	66441	Magohb	-2.02	down	0.00	1.89	up	0.02
419	XM_006523251	68842	Tulp4	-2.02	down	0.00	1.64	up	0.00
420	NM_001177750	1E+08	Gm10767	-2.02	down	0.00	1.58	up	0.02
421	NM_001290196	76803	2410141K09Rik	-2.02	down	0.00	1.46	up	0.03
422	NM_001081193	380664	Lemd3	-2.02	down	0.00	1.73	up	0.01
423	NM_001127189	1E+08	Gm13157	-2.02	down	0.00	1.58	up	0.01
424	NM_001008421	217431	Nol10	-2.02	down	0.00	1.75	up	0.04
425	NM_009466	22235	Ugdh	-2.02	down	0.00	1.56	up	0.02
426	NM_181322	13018	Ctcf	-2.02	down	0.00	1.76	up	0.01
427	ENSMUST00000181799			-2.02	down	0.00	1.59	up	0.00
428	AK042006	77766	Elp4	-2.02	down	0.01	1.43	up	0.01

429	NM_175028	240442	Adnp2	-2.02	down	0.00	1.33	up	0.04
430	NM_029606	76380	Cep112	-2.02	down	0.00	1.77	up	0.01
431	NM_020588	57439	Tmem183a	-2.02	down	0.01	1.99	up	0.01
432	NM_030261	75747	Sesn3	-2.02	down	0.00	1.99	up	0.00
433	NM_026246	18120	Mrpl49	-2.01	down	0.00	1.88	up	0.01
434	NM_025800	66849	Ppp1r2	-2.01	down	0.00	1.77	up	0.02
435	ENSMUST00000091097			-2.01	down	0.00	1.36	up	0.02
436	NM_172854	241327	Olfml2a	-2.01	down	0.01	1.72	up	0.04
437	NM_013703	22359	Vldlr	-2.01	down	0.00	1.56	up	0.04
438	ENSMUST00000029106	72147	Zbtb46	-2.01	down	0.00	1.51	up	0.03
439	ENSMUST00000119351			-2.01	down	0.00	1.38	up	0.02
440	NM_001038015	67980	Gnpda2	-2.01	down	0.00	1.74	up	0.01
441	ENSMUST00000121873			-2.01	down	0.00	1.62	up	0.00
442	NM_025682	66645	Pspc1	-2.01	down	0.00	1.28	up	0.02
443	ENSMUST00000071770			-2.01	down	0.00	1.58	up	0.03
444	NM_078477	118445	Klf16	-2.01	down	0.02	1.63	up	0.01
445	NM_013673	20684	Sp100	-2.01	down	0.01	1.54	up	0.04
446	NM_001167885	225888	Suv420h1	-2.01	down	0.00	1.52	up	0.03
447	NM_033564	93734	Mpv17l	-2.01	down	0.00	1.82	up	0.01
448	NM_001001185	408058	BC048507	-2.01	down	0.01	1.71	up	0.03
449	NAP095730-001			-2.01	down	0.00	1.69	up	0.01
450	NM_009879	12589	Ift81	-2.01	down	0.00	1.65	up	0.00
451	NM_011134	18979	Pon1	-2.01	down	0.05	2.00	up	0.04
452	NM_175515	380614	Intu	-2.01	down	0.01	1.63	up	0.02
453	NM_029512	76080	Ttpal	-2.01	down	0.00	1.62	up	0.01
454	NM_001160399	240261	Ccdc112	-2.01	down	0.01	1.73	up	0.03
455	ENSMUST00000121247			-2.01	down	0.00	1.81	up	0.01
456	NM_133832	98711	Rdh10	-2.00	down	0.00	1.66	up	0.01
457	NM_026055	67248	Rpl39	-2.00	down	0.00	1.45	up	0.03
458	NM_172054	98258	Txndc9	-2.00	down	0.00	1.47	up	0.00
459	NM_001044386	22764	Zfx	-2.00	down	0.00	1.50	up	0.02

460	NM_026503	68002	Sdhaf4	-2.00	down	0.00	1.66	up	0.03
461	NM_001081680	238722	Zfp72	-2.00	down	0.00	1.71	up	0.02
462	ENSMUST00000163337			-2.00	down	0.00	1.41	up	0.02
463	NM_011508	20918	Eif1	-2.00	down	0.00	1.65	up	0.01
464	AK139740			-2.00	down	0.00	1.61	up	0.00
465	NM_001033422	331401	Thoc2	-2.00	down	0.00	1.31	up	0.02
466	NM_175025	235574	Atp2c1	-2.00	down	0.00	1.63	up	0.00
467	NM_001083927	21887	Tle3	-2.00	down	0.02	1.65	up	0.02
468	NM_010085	13525	Adam26a	2.00	up	0.00	-1.40	down	0.01
469	NM_001080934	217316	Slc16a5	2.00	up	0.00	-1.56	down	0.02
470	ENSMUST00000127159	78656	Brd8	2.00	up	0.00	-1.96	down	0.00
471	NM_147003	259005	Olfr139	2.00	up	0.00	-1.48	down	0.03
472	NM_146453	258445	Olfr693	2.01	up	0.05	-1.89	down	0.00
473	NM_001002896	107993	Bfsp2	2.01	up	0.00	-1.56	down	0.00
474	NM_001159775	258236	Olfr391-ps	2.02	up	0.00	-1.86	down	0.00
475	NM_007439	11682	Alk	2.02	up	0.00	-1.46	down	0.02
476	NM_001113187	98582	Khdc1b	2.02	up	0.00	-1.45	down	0.01
477	NM_010929	18132	Notch4	2.02	up	0.00	-1.81	down	0.01
478	NM_027442	70503	Ddo	2.02	up	0.00	-1.65	down	0.00
479	AK047978	414118	Zmiz1os1	2.02	up	0.00	-1.55	down	0.00
480	NM_029689	76651	1700122O11Rik	2.02	up	0.00	-1.63	down	0.01
481	AK089900	78610	Uvrag	2.02	up	0.00	-1.71	down	0.01
482	AK087460			2.02	up	0.00	-1.80	down	0.00
483	ENSMUST00000029440	99543	Olfml3	2.02	up	0.00	-1.55	down	0.01
484	NM_008435	16538	Kcns1	2.03	up	0.00	-1.76	down	0.00
485	AK031474	382062	AB124611	2.03	up	0.00	-1.50	down	0.00
486	AK007903	69818	1810059C17Rik	2.03	up	0.00	-1.89	down	0.00
487	NM_017461	54204	43709	2.03	up	0.00	-1.52	down	0.01
488	NM_026685	68344	Tmem174	2.03	up	0.00	-1.76	down	0.01
489	ENSMUST00000130479	319845	Bbs9	2.03	up	0.00	-1.39	down	0.02
490	AK029733	666539	Gm8154	2.04	up	0.00	-1.55	down	0.01

491	NM_173070	229562	Sprr4	2.04	up	0.01	-1.73	down	0.00
492	ENSMUST00000145908	330222	Sdk1	2.04	up	0.00	-1.93	down	0.00
493	AK034480			2.04	up	0.02	-1.79	down	0.01
494	NM_009987	13051	Cx3cr1	2.04	up	0.00	-1.73	down	0.02
495	NM_013887	30044	Opn4	2.04	up	0.00	-1.69	down	0.00
496	NM_026338	67722	Actl11	2.04	up	0.01	-1.70	down	0.00
497	AK182364	11821	Aprt	2.05	up	0.00	-1.54	down	0.00
498	NM_133204	170734	Zscan5b	2.05	up	0.00	-1.82	down	0.00
499	NM_001101463	231885	Gm4871	2.05	up	0.00	-1.56	down	0.00
500	NM_183224	329731	Fam19a3	2.05	up	0.00	-1.65	down	0.01
501	NM_175280	78774	Cfap61	2.05	up	0.00	-1.78	down	0.01
502	NM_026785	68612	Ube2c	2.05	up	0.00	-1.32	down	0.04
503	AK082467	110333	Rmst	2.05	up	0.00	-1.72	down	0.01
504	XM_006506255	320127	Dgki	2.06	up	0.02	-1.83	down	0.00
505	NM_001289756	11814	Apoc3	2.06	up	0.02	-1.97	down	0.00
506	NM_146833	258830	Olfr103	2.06	up	0.00	-1.44	down	0.00
507	NM_021291	30962	Slc7a9	2.07	up	0.00	-1.86	down	0.00
508	NM_009470	22242	Umod	2.07	up	0.00	-1.77	down	0.02
509	NM_010057	13396	Dlx6	2.07	up	0.01	-1.45	down	0.02
510	AA822459			2.07	up	0.00	-1.61	down	0.01
511	NM_032610	80297	Sptbn4	2.07	up	0.00	-1.78	down	0.01
512	NM_144835	217995	Heatr1	2.08	up	0.00	-1.91	down	0.00
513	BI661026			2.08	up	0.00	-1.80	down	0.01
514	NM_146600	258593	Olfr700	2.08	up	0.00	-1.59	down	0.04
515	NM_011771	22780	Ikzf3	2.08	up	0.01	-1.94	down	0.00
516	NM_009036	19668	Rbpjl	2.08	up	0.02	-1.50	down	0.00
517	NM_133894	100559	Ugt2b38	2.09	up	0.03	-1.70	down	0.01
518	NM_001293795	107770	Tm6sf2	2.09	up	0.00	-1.40	down	0.00
519	AK131776	1E+08	Gm40421	2.09	up	0.00	-1.81	down	0.02
520	NM_001145537	668433	4930544D05Rik	2.09	up	0.00	-1.53	down	0.00
521	XM_006511606	1E+08	Gm10634	2.09	up	0.00	-1.48	down	0.01

522	NM_133254	246787	Slc5a2	2.09	up	0.00	-1.43	down	0.00
523	NM_172550	216119	Ybey	2.09	up	0.03	-1.89	down	0.00
524	NM_023670	140488	Igf2bp3	2.09	up	0.05	-1.59	down	0.00
525	ENSMUST00000119243			2.09	up	0.02	-1.73	down	0.02
526	NM_025520	66373	Lsm5	2.09	up	0.00	-1.94	down	0.00
527	BC107389	382913	Neil2	2.10	up	0.00	-1.33	down	0.01
528	NM_010235	14283	Fosl1	2.10	up	0.00	-1.81	down	0.00
529	ENSMUST00000105802	15565	Htr6	2.11	up	0.02	-1.62	down	0.01
530	ENSMUST00000103672			2.11	up	0.01	-1.57	down	0.01
531	NM_027024	69294	Cst13	2.11	up	0.00	-1.94	down	0.01
532	NM_194268	225631	Onecut2	2.11	up	0.02	-1.51	down	0.05
533	ENSMUST00000151328	17901	Myl1	2.12	up	0.00	-1.63	down	0.00
534	ENSMUST00000186455	81907	Tmem108	2.12	up	0.00	-1.97	down	0.00
535	NM_001029935	214158	Trim38	2.12	up	0.00	-1.53	down	0.01
536	NM_010287	14608	Gpr83	2.12	up	0.00	-1.73	down	0.05
537	ENSMUST00000120250			2.12	up	0.02	-1.91	down	0.00
538	NM_134193	171227	Vmn1r232	2.12	up	0.03	-1.80	down	0.04
539	AK051890	791272	Gm10091	2.13	up	0.00	-1.66	down	0.00
540	NM_007401	11499	Adam5	2.13	up	0.00	-1.57	down	0.00
541	NM_025487	66322	1700011A15Rik	2.13	up	0.00	-1.48	down	0.05
542	BF319608			2.13	up	0.00	-1.81	down	0.00
543	NM_027402	384061	Fndc5	2.13	up	0.00	-1.91	down	0.00
544	XM_006497797	19730	Ralgds	2.14	up	0.00	-1.99	down	0.00
545	AK040132			2.14	up	0.01	-1.84	down	0.02
546	ENSMUST00000127388	17756	Map2	2.14	up	0.00	-1.95	down	0.00
547	NM_008665	17932	Myt1	2.14	up	0.03	-1.72	down	0.04
548	ENSMUST00000107564	72324	Plxdc1	2.14	up	0.00	-1.48	down	0.04
549	AK007031	73588	St8sia3os	2.14	up	0.00	-1.58	down	0.01
550	NM_146904	57251	Olfr870	2.15	up	0.00	-1.64	down	0.00
551	NM_001123367	1E+08	Gm3448	2.15	up	0.00	-1.85	down	0.00
552	AK053272	1E+08	Gm9963	2.15	up	0.00	-1.76	down	0.00

553	AK171018	69305	Dcps	2.16	up	0.00	-1.86	down	0.00
554	AK133420	12983	Csf2rb	2.16	up	0.00	-1.24	down	0.02
555	NM_177354	238328	Vash1	2.16	up	0.00	-1.66	down	0.01
556	NM_153519	213272	Txndc2	2.16	up	0.00	-1.80	down	0.00
557	DV059070	109215	Lncbate1	2.16	up	0.00	-1.78	down	0.00
558	NM_053118	93746	Gprc5d	2.16	up	0.00	-1.90	down	0.00
559	NM_010824	17523	Mpo	2.16	up	0.01	-1.47	down	0.01
560	NM_001081397	244281	Myo16	2.16	up	0.00	-1.41	down	0.01
561	AK042193	320763	A430034D21Rik	2.16	up	0.00	-1.75	down	0.01
562	XM_006535747	50791	Magi2	2.16	up	0.00	-1.38	down	0.02
563	NM_013631	18770	Pklr	2.17	up	0.00	-1.37	down	0.00
564	BC114968	59005	Trappc2l	2.17	up	0.01	-1.58	down	0.03
565	AK154713	381680	Nxpe5	2.17	up	0.01	-1.78	down	0.00
566	NM_009659	11686	Alox12b	2.17	up	0.00	-1.97	down	0.00
567	NM_009602	11444	Chrnb2	2.17	up	0.01	-2.00	down	0.00
568	NM_199257	234129	Tpte	2.17	up	0.00	-1.53	down	0.01
569	NM_028852	74281	Spatc1	2.17	up	0.02	-1.43	down	0.04
570	AK217931	80987	Nckipsd	2.17	up	0.00	-1.85	down	0.00
571	NM_001011814	258055	Olfr524	2.17	up	0.00	-1.84	down	0.00
572	NM_008657	17878	Myf6	2.17	up	0.04	-1.33	down	0.01
573	ENSMUST00000053635			2.18	up	0.00	-1.51	down	0.00
574	NM_008375	16204	Fabp6	2.19	up	0.00	-1.57	down	0.00
575	NM_001114679	667214	9930111J21Rik1	2.19	up	0.00	-1.95	down	0.00
576	AK084558			2.19	up	0.00	-1.85	down	0.01
577	NM_007919	13706	Cela2a	2.19	up	0.00	-1.61	down	0.00
578	NM_019474	29846	Olfr156	2.19	up	0.01	-1.97	down	0.00
579	BC048482	27281	Hrasls	2.19	up	0.00	-1.93	down	0.00
580	NM_008729	18163	Ctnnd2	2.19	up	0.00	-1.80	down	0.02
581	BC042796	1E+08	Gm32478	2.20	up	0.00	-1.84	down	0.01
582	NM_010299	14667	Gm2a	2.20	up	0.03	-1.91	down	0.00
583	BC103784	633640	Gm7120	2.20	up	0.00	-1.64	down	0.04

584	NM_207235	227789	Olfr358	2.21	up	0.00	-1.92	down	0.00
585	NM_008936	19127	Prop1	2.21	up	0.00	-1.88	down	0.00
586	NM_207230	216783	Olfr320	2.21	up	0.00	-1.52	down	0.01
587	NM_001013411	432450	Nkain2	2.21	up	0.00	-1.63	down	0.00
588	ENSMUST00000143224	546134	Gramd2	2.21	up	0.00	-1.60	down	0.02
589	ENSMUST00000088673			2.21	up	0.00	-1.94	down	0.00
590	NM_008353	16161	Il12rb1	2.22	up	0.00	-1.43	down	0.01
591	NM_001029933	232966	Zfp114	2.22	up	0.01	-1.47	down	0.01
592	NM_198106	208169	Slc9c1	2.23	up	0.01	-1.87	down	0.01
593	ENSMUST00000100417	14055	Ezh1	2.23	up	0.01	-1.83	down	0.00
594	NM_027908	71761	Amdhd1	2.23	up	0.00	-1.83	down	0.00
595	NM_001085528	435791	Gm13271	2.23	up	0.00	-1.72	down	0.00
596	AA267875			2.23	up	0.00	-1.69	down	0.00
597	AK019365	68191	Taco1os	2.24	up	0.00	-1.82	down	0.00
598	NM_144810	213417	Klhdc8a	2.24	up	0.02	-1.58	down	0.00
599	ENSMUST00000150402	15247	Hiat1	2.25	up	0.00	-1.88	down	0.00
600	ENSMUST00000176687			2.25	up	0.02	-1.29	down	0.02
601	AK020279	77123	9130214F15Rik	2.25	up	0.01	-1.79	down	0.00
602	BC058197	76051	Ganc	2.25	up	0.01	-1.56	down	0.00
603	NM_001167746	69926	Dnah17	2.25	up	0.00	-1.87	down	0.00
604	NM_008205	14997	H2-M9	2.26	up	0.01	-1.72	down	0.00
605	NM_146666	258660	Olfr736	2.26	up	0.00	-1.49	down	0.05
606	CD548909			2.26	up	0.00	-1.81	down	0.00
607	BX519376	98401	AI594674	2.26	up	0.01	-1.59	down	0.02
608	NM_007550	12144	Blm	2.26	up	0.00	-1.84	down	0.00
609	AK042948	1E+08	LOC101056015	2.27	up	0.00	-1.85	down	0.00
610	AK028492			2.27	up	0.00	-1.47	down	0.01
611	AK015936	75231	4930529I22Rik	2.27	up	0.00	-1.65	down	0.00
612	XM_006515852	238331	Zdhhc22	2.27	up	0.00	-1.75	down	0.00
613	NM_022886	64929	Scel	2.27	up	0.00	-1.79	down	0.00
614	NM_026421	67870	Enoph1	2.28	up	0.00	-1.73	down	0.01

615	AK040329	84544	Cd96	2.28	up	0.00	-1.88	down	0.01
616	NM_029064	74711	Ttll9	2.29	up	0.00	-1.97	down	0.02
617	NM_028749	74091	Npl	2.29	up	0.01	-1.70	down	0.04
618	NM_153106	242726	Padi6	2.29	up	0.01	-1.60	down	0.00
619	ENSMUST00000173109	53357	Pla2g6	2.30	up	0.00	-1.28	down	0.02
620	NM_023370	22295	Cdh23	2.31	up	0.00	-1.88	down	0.00
621	NM_146739	258734	Olfr502	2.32	up	0.00	-1.49	down	0.02
622	NM_029310	75497	Fabp12	2.32	up	0.00	-1.90	down	0.00
623	NM_146967	258969	Olfr1226	2.33	up	0.00	-1.59	down	0.01
624	AK164443	21975	Top3a	2.33	up	0.00	-1.80	down	0.00
625	BC022662			2.33	up	0.02	-1.66	down	0.00
626	NM_009491	22307	Vmn2r10	2.33	up	0.00	-1.87	down	0.01
627	AK080358	668311	Gm9099	2.34	up	0.00	-1.73	down	0.01
628	XM_011246586	68327	Tsr3	2.34	up	0.00	-1.98	down	0.00
629	NM_001011518	258219	Olfr94	2.34	up	0.00	-1.83	down	0.00
630	NM_138673	192188	Stab2	2.34	up	0.00	-1.38	down	0.01
631	XM_006531872	225852	Gm550	2.34	up	0.00	-1.54	down	0.01
632	NM_177791	279499	Kctd19	2.35	up	0.01	-1.62	down	0.02
633	DV065341	72767	2810427A07Rik	2.35	up	0.00	-1.69	down	0.00
634	NM_178412	229688	Chil6	2.35	up	0.02	-1.67	down	0.00
635	ENSMUST00000195258	19206	Ptch1	2.36	up	0.00	-1.70	down	0.01
636	AK090034	1E+08	Gm3161	2.36	up	0.00	-1.85	down	0.00
637	AK136711	244757	Glb1l2	2.36	up	0.00	-1.56	down	0.00
638	NM_030100	78428	Wibg	2.37	up	0.00	-1.95	down	0.00
639	NM_007709	12705	Cited1	2.37	up	0.00	-1.73	down	0.00
640	NM_134220	171254	Vmn1r198	2.38	up	0.00	-1.73	down	0.00
641	NM_133720	70086	Cysltr2	2.38	up	0.00	-1.97	down	0.00
642	XM_006504899	1E+08	Gm15411	2.39	up	0.00	-1.76	down	0.00
643	NM_027790	71412	Dhrs2	2.39	up	0.00	-1.85	down	0.00
644	NM_134212	171246	Vmn1r200	2.39	up	0.01	-1.60	down	0.01
645	ENSMUST00000130873	12111	Bgn	2.40	up	0.04	-1.83	down	0.01

646	NM_172900	243958	Siglecg	2.40	up	0.02	-1.61	down	0.02
647	XM_006520654	20273	Scn8a	2.40	up	0.00	-1.99	down	0.00
648	NM_146353	258350	Olfr706	2.40	up	0.00	-1.56	down	0.02
649	ENSMUST00000140066	54139	Irf6	2.40	up	0.00	-2.00	down	0.00
650	ENSMUST00000110031	11992	Auh	2.41	up	0.00	-1.92	down	0.00
651	ENSMUST00000146477	99237	Tm9sf4	2.42	up	0.00	-1.91	down	0.00
652	CK334688	105271	AU017674	2.43	up	0.00	-1.55	down	0.01
653	ENSMUST00000190711	14859	Gsta3	2.43	up	0.01	-1.63	down	0.00
654	XM_011242052	24099	Tnfsf13b	2.44	up	0.00	-1.50	down	0.01
655	AK031735			2.44	up	0.00	-1.92	down	0.00
656	NM_021340	57811	Rgr	2.44	up	0.03	-1.68	down	0.01
657	ENSMUST00000146433			2.44	up	0.01	-1.56	down	0.00
658	XM_006516464	116852	Akr1c20	2.44	up	0.02	-1.53	down	0.00
659	NM_011741	22635	Zan	2.45	up	0.00	-1.84	down	0.00
660	NM_198628	279029	Stkld1	2.45	up	0.01	-1.54	down	0.01
661	NM_001301682	330470	Bsph1	2.46	up	0.00	-1.88	down	0.00
662	AK046275	75868	4930588A03Rik	2.47	up	0.00	-1.51	down	0.00
663	NM_029440	381693	Wdr95	2.48	up	0.00	-1.79	down	0.00
664	ENSMUST00000119421			2.48	up	0.00	-1.87	down	0.00
665	ENSMUST00000117983			2.49	up	0.00	-1.82	down	0.00
666	ENSMUST00000120183			2.49	up	0.03	-1.98	down	0.01
667	NM_027104	71886	2310002L09Rik	2.49	up	0.00	-1.33	down	0.05
668	ENSMUST00000121277			2.50	up	0.00	-1.82	down	0.00
669	AK135767	629734	Gm6999	2.50	up	0.00	-1.56	down	0.01
670	NM_016718	29862	Ninj2	2.51	up	0.00	-1.80	down	0.01
671	NM_007720	12776	Ccr8	2.51	up	0.00	-1.73	down	0.00
672	NM_029031	74637	Shpk	2.52	up	0.00	-1.90	down	0.00
673	NM_009362	21784	Tff1	2.52	up	0.00	-1.77	down	0.00
674	NM_007860	13370	Dio1	2.53	up	0.00	-1.65	down	0.00
675	AK160242	665225	Gm7544	2.54	up	0.00	-1.77	down	0.01
676	NM_001291001	70426	Tekt5	2.55	up	0.00	-1.96	down	0.00

677	AK037200	1E+08	Gm26795	2.56	up	0.00	-1.45	down	0.00
678	NM_153598	100727	Ugt2b34	2.56	up	0.00	-2.00	down	0.01
679	AK033044	360222	Raxos1	2.57	up	0.00	-1.74	down	0.01
680	NM_183288	544817	Arhgap27	2.58	up	0.00	-1.86	down	0.02
681	NM_011978	26458	Slc27a2	2.58	up	0.02	-1.36	down	0.00
682	AK082204			2.58	up	0.02	-1.35	down	0.03
683	NM_007407	11517	Adcyap1r1	2.58	up	0.00	-1.63	down	0.00
684	NM_001164682	227157	Mpp4	2.59	up	0.00	-1.61	down	0.00
685	ENSMUST00000121186			2.59	up	0.01	-1.44	down	0.00
686	XM_006524267	240119	St6gal2	2.60	up	0.00	-1.59	down	0.00
687	NM_001127686	436003	Hbb-bh2	2.60	up	0.00	-1.52	down	0.00
688	NM_027600	70909	4921504E06Rik	2.60	up	0.00	-1.87	down	0.01
689	NM_025621	66533	2310050C09Rik	2.61	up	0.00	-1.76	down	0.03
690	AK155734	1E+08	AK155734	2.64	up	0.00	-1.50	down	0.00
691	ENSMUST00000124702	229277	Stoml3	2.67	up	0.00	-1.76	down	0.00
692	ENSMUST00000142944	21833	Thra	2.69	up	0.00	-1.35	down	0.01
693	NM_145592	234130	Dkk4	2.70	up	0.00	-1.90	down	0.00
694	NM_027105	69533	Krtap26-1	2.71	up	0.00	-1.84	down	0.00
695	NM_001080809	227231	Cps1	2.72	up	0.00	-1.82	down	0.01
696	NM_026516	68027	Tmem178	2.72	up	0.00	-1.98	down	0.00
697	NM_029464	75860	Tex26	2.73	up	0.01	-1.57	down	0.00
698	TC1666663			2.73	up	0.00	-1.73	down	0.00
699	NM_174865	317652	Klk15	2.73	up	0.00	-1.69	down	0.00
700	ENSMUST00000043170			2.74	up	0.00	-1.98	down	0.00
701	NM_019471	17384	Mmp10	2.75	up	0.00	-1.67	down	0.02
702	ENSMUST00000147330	12704	Cit	2.76	up	0.00	-1.54	down	0.01
703	AK141100	320684	9630028H03Rik	2.77	up	0.00	-1.70	down	0.01
704	NM_177591	209268	Igsf1	2.78	up	0.00	-1.56	down	0.00
705	ENSMUST00000143959	71382	Pex1	2.78	up	0.00	-1.82	down	0.00
706	ENSMUST00000155469	13839	Epha5	2.81	up	0.00	-1.54	down	0.03
707	XM_006544087		LOC102641508	2.81	up	0.00	-1.99	down	0.00

708	NM_010127	19009	Pou6f1	2.82	up	0.01	-1.79	down	0.01
709	NM_177900	330790	Hapln4	2.87	up	0.00	-1.33	down	0.04
710	AK218396			2.87	up	0.00	-1.93	down	0.00
711	NM_178673	213262	Fstl5	2.90	up	0.00	-1.48	down	0.01
712	NM_182957	66773	Gm17019	2.92	up	0.00	-1.76	down	0.00
713	ENSMUST00000069035			2.95	up	0.00	-1.72	down	0.01
714	XM_006524202	225049	Ttc7	2.97	up	0.00	-1.75	down	0.00
715	NM_146951	258953	Olfr340	2.98	up	0.01	-1.44	down	0.01
716	NM_001033238	208650	Cblb	2.99	up	0.00	-1.85	down	0.00
717	NM_001013753	219228	Pcdh17	3.00	up	0.00	-1.72	down	0.00
718	NM_028033	71979	2410012M07Rik	3.00	up	0.00	-1.92	down	0.00
719	NM_001011803	258023	Olfr1306	3.01	up	0.00	-1.71	down	0.00
720	NM_178247	347708	Dppa1	3.03	up	0.01	-1.59	down	0.00
721	AK029692	74923	4930471D02Rik	3.05	up	0.00	-1.68	down	0.01
722	NM_023816	76389	Ankrd36	3.08	up	0.00	-1.85	down	0.00
723	XM_006528656		LOC101055948	3.12	up	0.00	-1.46	down	0.01
724	XM_006517153	18548	Pcsk1	3.14	up	0.00	-1.94	down	0.00
725	NM_008811	18598	Pdha2	3.15	up	0.00	-1.70	down	0.00
726	AK020961	77981	B230110C06Rik	3.18	up	0.00	-1.99	down	0.05
727	NM_010420	15209	Hesx1	3.24	up	0.00	-1.99	down	0.01
728	NM_183031	321019	Gpr183	3.25	up	0.00	-1.75	down	0.00
729	NM_009903	12740	Cldn4	3.26	up	0.01	-1.93	down	0.01
730	AK086943	629554	Gm6980	3.28	up	0.00	-1.62	down	0.00
731	NM_011440	20669	Sox14	3.28	up	0.00	-1.81	down	0.00
732	ENSMUST00000107861	72605	Car10	3.35	up	0.00	-1.82	down	0.00
733	AK088235	70966	4931415C17Rik	3.35	up	0.00	-1.95	down	0.01
734	NM_178245	244813	Bsx	3.39	up	0.00	-1.86	down	0.01
735	BI499719			3.45	up	0.00	-1.64	down	0.01
736	NM_001159389	320995	Rfx6	3.55	up	0.00	-1.75	down	0.00
737	ENSMUST00000154712	75062	Sf3a3	3.55	up	0.00	-1.80	down	0.00
738	NM_023894	104384	Rhox9	3.69	up	0.00	-1.97	down	0.00

739	ENSMUST00000116442	15444	Hpca	3.71	up	0.00	-1.87	down	0.03
740	NM_173409	233918	4933402N03Rik	3.74	up	0.00	-1.74	down	0.01
741	NM_194333	22626	Slc23a3	3.80	up	0.00	-1.87	down	0.01
742	NM_176951	319581	Xkr5	4.30	up	0.00	-1.68	down	0.02
743	NM_001112744	230972	Arhgef16	10.18	up	0.00	-1.66	down	0.01

No. number; IH, chronic intermittent hypoxia; N, Normoxia; RNpIH, Re-Normoxia-post-chronic intermittent hypoxia; Reg.regulation

Table S2. KEGG pathway analysis of the differentially expressed proteins in aortas from CIH-treated mice.

**Term_ID**	**Term_description**	**Gene Ratio**	**Bg Ratio**	**P value**	**FDR bh**	**Fold**	**GeneSymbols**
path:mmu03013	RNA transport	0.058	0.021	0.001	0.133	2.815	Eif1;Gm5415;Gm9839;Ncbp2;Nup37;Thoc2;Rbm8a;Nup153;Eif1ax;Eif3j2; Pym1;Magohb;Kpnb1
path:mmu03040	Spliceosome	0.049	0.016	0.001	0.133	2.991	Ncbp2;Sf3a3;Thoc2;Ppih;Magohb;Rbm22;Hspa8;Sf3b6;Crnkl1;Rbm8a;Lsm5
path:mmu00053	Ascorbate and aldarate metabolism	0.018	0.003	0.006	0.369	5.357	Ugdh;Ugt2b34;Ugt2a3;Ugt2b38
path:mmu04146	Peroxisome	0.031	0.010	0.008	0.369	3.013	Ddo;Slc27a2;Idh1;Gstk1;Mpv17l;Pex1;Acsl3
path:mmu00980	Metabolism of xenobiotics by cytochrome P450	0.027	0.008	0.009	0.369	3.338	Gsta3;Ugt2b34;Gstk1;Ugt2b38;Ugt2a3;Cyp2f2
path:mmu00982	Drug metabolism	0.027	0.008	0.010	0.369	3.238	Gsta3;Ugt2b34;Gstk1;Ugt2b38;Ugt2a3;Fmo5
path:mmu00520	Amino sugar and nucleotide sugar metabolism	0.022	0.006	0.011	0.369	3.690	Gnpda2;Npl;Ugdh;Fpgt;Cmas
path:mmu04612	Antigen processing and presentation	0.031	0.011	0.013	0.369	2.782	Creb1;Nfyb;H2-M9;Hsp90aa1;Rfxap;Hspa8;Canx
path:mmu00040	Pentose and glucuronate interconversions	0.018	0.004	0.014	0.369	4.254	Ugdh;Ugt2b34;Ugt2a3;Ugt2b38
path:mmu00860	Porphyrin and chlorophyll metabolism	0.018	0.005	0.026	0.625	3.528	Cox10;Ugt2b34;Ugt2a3;Ugt2b38
path:mmu00592	alpha-Linolenic acid metabolism	0.013	0.003	0.031	0.672	4.339	Pla2g5;Pla2g6;Plb1
path:mmu00565	Ether lipid metabolism	0.018	0.006	0.035	0.704	3.214	Pla2g5;Enpp6;Pla2g6;Plb1
path:mmu04211	Longevity regulating pathway	0.027	0.011	0.038	0.706	2.411	Creb1;Atf4;Rheb;Rb1cc1;Creb5;Sesn3
path:mmu04330	Notch signaling pathway	0.018	0.006	0.046	0.786	2.952	Aph1b;Aph1c;Rbpjl;Notch4
path:mmu04310	Wnt signaling pathway	0.036	0.018	0.049	0.786	1.981	Gm5415;Gm9839;Fosl1;Cacybp;Ccnd2;Dkk4;Sfrp1;Myc
path:mmu00983	Drug metabolism	0.018	0.006	0.055	0.786	2.782	Umps;Ugt2b34;Ugt2a3;Ugt2b38

path:mmu05203	Viral carcinogenesis	0.049	0.028	0.055	0.786	1.722	Creb1;Gtf2a1;H2-M9;Rbpjl;Ube3a;Atf4;Creb5;Ccnd2;Gtf2h1;Sp100;Ccr8
path:mmu05143	African trypanosomiasis	0.013	0.004	0.076	0.925	3.013	Il12a;Fasl;Vcam1
path:mmu03018	RNA degradation	0.022	0.010	0.079	0.925	2.178	Btg3;Exosc3;Dcps;Lsm5;Hspd1
path:mmu00620	Pyruvate metabolism	0.013	0.005	0.087	0.925	2.855	Acyp1;Pklr;Pdha2
path:mmu00480	Glutathione metabolism	0.018	0.007	0.088	0.925	2.371	Ggct;Gsta3;Idh1;Gstk1
path:mmu04120	Ubiquitin mediated proteolysis	0.031	0.017	0.093	0.925	1.808	Anapc10;Ube3a;Ube2w;Ube2c;Cblb;Uba3;Cul5
path:mmu04919	Thyroid hormone signaling pathway	0.027	0.014	0.099	0.925	1.887	Thra;Rheb;Slc16a10;Dio1;Notch4;Myc
path:mmu00590	Arachidonic acid metabolism	0.022	0.011	0.100	0.925	2.031	Pla2g6;Alox12b;Pla2g5;Tbxas1;Plb1
path:mmu05204	Chemical carcinogenesis	0.022	0.011	0.115	0.925	1.944	Gsta3;Ugt2b34;Gstk1;Ugt2b38;Ugt2a3
path:mmu01200	Carbon metabolism	0.027	0.015	0.115	0.925	1.808	Pklr;Idh1;Mcee;Cps1;Mut;Pdha2
path:mmu04962	Vasopressin-regulated water reabsorption	0.013	0.005	0.115	0.925	2.523	Creb1;Dync2h1;Creb5
path:mmu05152	Tuberculosis	0.036	0.022	0.120	0.925	1.625	Creb1;Nfyb;Atp6v1h;Malt1;Rfxap;Hspd1;Il12a;Eea1
path:mmu04340	Hedgehog signaling pathway	0.013	0.005	0.121	0.925	2.465	Ptch1;Dhh;Ccnd2
path:mmu03022	Basal transcription factors	0.013	0.005	0.121	0.925	2.465	Gtf2a1;Gtf2h1;Taf9b
path:mmu03015	mRNA surveillance pathway	0.022	0.012	0.126	0.925	1.883	Ncbp2;Gspt1;Magohb;Pym1;Rbm8a
path:mmu04915	Estrogen signaling pathway	0.022	0.012	0.126	0.925	1.883	Creb1;Hsp90aa1;Atf4;Creb5;Hspa8
path:mmu04940	Type I diabetes mellitus	0.018	0.009	0.128	0.925	2.066	Il12a;H2-M9;Fasl;Hspd1
path:mmu05215	Prostate cancer	0.022	0.012	0.131	0.925	1.864	Creb1;Hsp90aa1;Atf4;Creb5;Braf
path:mmu04977	Vitamin digestion and absorption	0.009	0.003	0.141	0.954	3.013	Slc19a2;Plb1
path:mmu04918	Thyroid hormone synthesis	0.018	0.009	0.143	0.954	1.981	Creb1;Creb5;Atf4;Canx
path:mmu05030	Cocaine addiction	0.013	0.006	0.147	0.954	2.260	Creb1;Creb5;Atf4
path:mmu00591	Linoleic acid metabolism	0.013	0.006	0.160	0.980	2.170	Pla2g5;Pla2g6;Plb1
path:mmu01230	Biosynthesis of amino acids	0.018	0.009	0.164	0.980	1.878	Cth;Cps1;Pklr;Idh1
path:mmu03460	Fanconi anemia pathway	0.013	0.006	0.167	0.980	2.127	Rev1;Top3a;Blm
path:mmu04950	Maturity onset diabetes of the young	0.009	0.003	0.171	0.980	2.679	Rfx6;Pklr
path:mmu00630	Glyoxylate and dicarboxylate metabolism	0.009	0.004	0.191	0.980	2.494	Mcee;Mut
path:mmu05418	Fluid shear stress and atherosclerosis	0.027	0.017	0.200	0.980	1.528	Hsp90aa1;Gsta3;Klf2;Dusp1;Vcam1;Nfe2l2
path:mmu03020	RNA polymerase	0.009	0.004	0.201	0.980	2.411	Polr3k;Polr1d
path:mmu00280	Valine, leucine and isoleucine degradation	0.013	0.007	0.201	0.980	1.937	Mcee;Auh;Mut
path:mmu03320	PPAR signaling pathway	0.018	0.010	0.208	0.980	1.702	Apoc3;Slc27a2;Acsl3;Fabp6

path:mmu04911	Insulin secretion	0.018	0.010	0.208	0.980	1.702	Creb1;Creb5;Atf4;Adcyap1r1
path:mmu00640	Propanoate metabolism	0.009	0.004	0.211	0.980	2.333	Mcee;Mut
path:mmu05134	Legionellosis	0.013	0.007	0.216	0.980	1.870	Il12a;Hspd1;Hspa8
path:mmu04658	Th1 and Th2 cell differentiation	0.018	0.011	0.220	0.980	1.663	Il12a;Rbpjl;Nfkbie;Il12rb1
path:mmu00020	Citrate cycle (TCA cycle)	0.009	0.004	0.221	0.980	2.260	Idh1;Pdha2
path:mmu00310	Lysine degradation	0.013	0.007	0.223	0.980	1.839	Ezh1;Nsd1;Kmt5b
path:mmu04974	Protein digestion and absorption	0.018	0.011	0.238	0.980	1.607	Slc7a9;Slc16a10;Col11a1;Cela2a
path:mmu04914	Progesterone-mediated oocyte maturation	0.018	0.011	0.238	0.980	1.607	Braf;Hsp90aa1;Anapc10;Pgr
path:mmu00830	Retinol metabolism	0.018	0.011	0.244	0.980	1.589	Ugt2b34;Ugt2a3;Ugt2b38;Rdh10
path:mmu05166	HTLV-I infection	0.044	0.035	0.254	0.980	1.282	Creb1;Anapc10;H2-M9;Fosl1;Nfyb;Atf4;Ccnd2;Vcam1;Myc;Canx
path:mmu05330	Allograft rejection	0.013	0.008	0.260	0.980	1.695	Il12a;H2-M9;Fasl
path:mmu00920	Sulfur metabolism	0.004	0.001	0.266	0.980	3.287	Bpnt1
path:mmu00250	Alanine, aspartate and glutamate metabolism	0.009	0.005	0.273	0.980	1.955	Ddo;Cps1
path:mmu05216	Thyroid cancer	0.009	0.005	0.273	0.980	1.955	Braf;Myc
path:mmu00564	Glycerophospholipid metabolism	0.018	0.012	0.281	0.980	1.491	Dgki;Pla2g5;Pla2g6;Plb1
path:mmu04115	p53 signaling pathway	0.013	0.008	0.283	0.980	1.619	Ccng1;Sesn3;Ccnd2
path:mmu05031	Amphetamine addiction	0.013	0.008	0.290	0.980	1.595	Creb1;Creb5;Atf4
path:mmu05221	Acute myeloid leukemia	0.013	0.008	0.298	0.980	1.572	Braf;Myc;Mpo
path:mmu04726	Serotonergic synapse	0.022	0.016	0.302	0.980	1.370	Htr6;Alox12b;Dusp1;Trpc1;Braf
path:mmu00240	Pyrimidine metabolism	0.018	0.012	0.306	0.980	1.432	Umps;Pola1;Polr3k;Polr1d
path:mmu04630	Jak-STAT signaling pathway	0.027	0.020	0.306	0.980	1.315	Lepr;Ccnd2;Csf2rb;Il12a;Myc;Il12rb1
path:mmu04141	Protein processing in endoplasmic reticulum	0.027	0.020	0.311	0.980	1.307	Hsp90aa1;Atf4;Ubqln1;Hspa8;Nfe2l2;Canx
path:mmu04922	Glucagon signaling pathway	0.018	0.013	0.312	0.980	1.418	Creb1;Creb5;Atf4;Pdha2
path:mmu04920	Adipocytokine signaling pathway	0.013	0.009	0.313	0.980	1.528	Acsl3;Lepr;Nfkbie
path:mmu05219	Bladder cancer	0.009	0.005	0.314	0.980	1.764	Braf;Myc
path:mmu03440	Homologous recombination	0.009	0.005	0.314	0.980	1.764	Top3a;Blm
path:mmu05162	Measles	0.022	0.017	0.323	0.980	1.329	Ccnd2;Cblb;Hspa8;Il12a;Fasl
path:mmu00061	Fatty acid biosynthesis	0.004	0.002	0.325	0.980	2.583	Acsl3

path:mmu04672	Intestinal immune network for IgA production	0.009	0.005	0.335	0.980	1.682	Pigr;Tnfsf13b
path:mmu05210	Colorectal cancer	0.013	0.009	0.336	0.980	1.466	Braf;Ralgds;Myc
path:mmu04668	TNF signaling pathway	0.018	0.013	0.350	0.980	1.339	Creb1;Creb5;Atf4;Vcam1
path:mmu05161	Hepatitis B	0.022	0.018	0.362	0.980	1.264	Creb1;Atf4;Creb5;Fasl;Myc
path:mmu03010	Ribosome	0.027	0.022	0.365	0.980	1.226	Rpl39;Mrpl32;Rpl34-ps1;Rps12;Mrps6;Rpl36a
path:mmu05220	Chronic myeloid leukemia	0.013	0.010	0.367	0.980	1.391	Braf;Cblb;Myc
path:mmu00450	Selenocompound metabolism	0.004	0.002	0.380	0.980	2.127	Cth
path:mmu00910	Nitrogen metabolism	0.004	0.002	0.380	0.980	2.127	Cps1
path:mmu04725	Cholinergic synapse	0.018	0.014	0.381	0.980	1.280	Creb1;Chrnb2;Creb5;Atf4
path:mmu05144	Malaria	0.009	0.006	0.395	0.980	1.476	Il12a;Vcam1
path:mmu00270	Cysteine and methionine metabolism	0.009	0.006	0.395	0.980	1.476	Cth;Enoph1
path:mmu05202	Transcriptional misregulation in cancer	0.027	0.022	0.395	0.980	1.186	Myc;Ccnd2;Lmo2;Mpo;Mllt3;Bmi1
path:mmu04145	Phagosome	0.027	0.022	0.395	0.980	1.186	H2-M9;Atp6v1h;Mpo;Dync2h1;Eea1;Canx
path:mmu03008	Ribosome biogenesis in eukaryotes	0.018	0.014	0.400	0.980	1.247	Lsg1;Heatr1;Gar1;Riok1
path:mmu05169	Epstein-Barr virus infection	0.031	0.027	0.407	0.980	1.151	H2-M9;Rbpjl;Polr3k;Nfkbie;Polr1d;Hspa8;Myc
path:mmu00220	Arginine biosynthesis	0.004	0.002	0.413	0.980	1.903	Cps1
path:mmu01210	2-Oxocarboxylic acid metabolism	0.004	0.002	0.413	0.980	1.903	Idh1
path:mmu00100	Steroid biosynthesis	0.004	0.002	0.413	0.980	1.903	Msmo1
path:mmu04012	ErbB signaling pathway	0.013	0.010	0.419	0.980	1.276	Braf;Cblb;Myc
path:mmu01100	Metabolic pathways	0.169	0.162	0.420	0.980	1.041	St6gal2;Polr3k;Pklr;Idh1;Gnpda2;Alox12b;Ugt2b38;Fpgt;Bpnt1;Ganc;Amdhd1;Mcee;Aprt;Msmo1;B3galt1;Rdh10;Tbxas1;Enoph1;Pla2g6;Pola1;Atp6v1h;Pla2g5;Cps1;Ugt2a3;Ugdh;Ckb;Cox10;Mut;Cmas;Ugt2b34;Cth;Umps;Acsl3;Auh;Dgki;Polr1d;Plb1;Pdha2
path:mmu04722	Neurotrophin signaling pathway	0.018	0.015	0.431	0.980	1.195	Braf;Fasl;Nfkbie;Atf4
path:mmu00140	Steroid hormone biosynthesis	0.013	0.011	0.434	0.980	1.247	Ugt2b34;Ugt2a3;Ugt2b38
path:mmu04142	Lysosome	0.018	0.015	0.450	0.980	1.166	Lamp3;Gm2a;Ap3m2;Atp6v1h
path:mmu00514	Other types of O-glycan biosynthesis	0.004	0.003	0.461	0.980	1.644	St6gal2
path:mmu04152	AMPK signaling pathway	0.018	0.015	0.462	0.980	1.148	Creb1;Lepr;Creb5;Rheb
path:mmu04024	cAMP signaling pathway	0.027	0.024	0.464	0.980	1.101	Creb1;Htr6;Ptch1;Creb5;Braf;Adcyap1r1

path:mmu04144	Endocytosis	0.036	0.033	0.473	0.980	1.071	H2-M9;Cblb;Capza2;Snx1;Rbsn;Hspa8;Vps37a;Eea1
path:mmu00360	Phenylalanine metabolism	0.004	0.003	0.476	0.980	1.572	Mif
path:mmu04060	Cytokine-cytokine receptor interaction	0.036	0.033	0.477	0.980	1.067	Lepr;Cx3cr1;Il12rb1;Csf2rb;Il12a;Fasl;Tnfsf13b;Ccr8
path:mmu05213	Endometrial cancer	0.009	0.007	0.480	0.980	1.247	Braf;Myc
path:mmu04270	Vascular smooth muscle contraction	0.018	0.016	0.480	0.980	1.121	Braf;Pla2g6;Prkg1;Pla2g5
path:mmu05321	Inflammatory bowel disease (IBD)	0.009	0.007	0.489	0.980	1.226	Il12a;Il12rb1
path:mmu00340	Histidine metabolism	0.004	0.003	0.490	0.980	1.507	Amdhd1
path:mmu04068	FoxO signaling pathway	0.018	0.016	0.498	0.980	1.096	Braf;Fasl;Klf2;Ccnd2
path:mmu04730	Long-term depression	0.009	0.007	0.506	0.980	1.186	Braf;Prkg1
path:mmu04713	Circadian entrainment	0.013	0.012	0.512	0.980	1.107	Creb1;Prkg1;Adcyap1r1
path:mmu04721	Synaptic vesicle cycle	0.009	0.008	0.515	0.980	1.166	Cplx2;Atp6v1h
path:mmu05231	Choline metabolism in cancer	0.013	0.012	0.519	0.980	1.096	Dgki;Ralgds;Rheb
path:mmu05200	Pathways in cancer	0.067	0.066	0.520	0.980	1.012	Gsta3;Hsp90aa1;Ptch1;Nfe2l2;Ralgds;Ccnd2;Alk;Cblb;Notch4;Csf2rb;Braf; Il12a;Fasl;Myc;Il12rb1
path:mmu04210	Apoptosis	0.018	0.017	0.522	0.980	1.064	Csf2rb;Sept4;Fasl;Atf4
path:mmu04925	Aldosterone synthesis and secretion	0.013	0.012	0.526	0.980	1.085	Creb1;Creb5;Atf4
path:mmu00601	Glycosphingolipid biosynthesis	0.004	0.003	0.532	0.980	1.339	B3galt1
path:mmu05230	Central carbon metabolism in cancer	0.009	0.008	0.532	0.980	1.130	Myc;Pdha2
path:mmu04623	Cytosolic DNA-sensing pathway	0.009	0.008	0.532	0.980	1.130	Polr3k;Polr1d
path:mmu04360	Axon guidance	0.022	0.022	0.535	0.980	1.033	Sema3a;Ptch1;Sema4g;Epha5;Trpc1
path:mmu04659	Th17 cell differentiation	0.013	0.013	0.539	0.980	1.064	Hsp90aa1;Nfkbie;Il12rb1
path:mmu05332	Graft-versus-host disease	0.009	0.008	0.541	0.980	1.113	H2-M9;Fasl
path:mmu04660	T cell receptor signaling pathway	0.013	0.013	0.546	0.980	1.053	Malt1;Cblb;Nfkbie
path:mmu04972	Pancreatic secretion	0.013	0.013	0.546	0.980	1.053	Trpc1;Pla2g5;Cela2a
path:mmu05223	Non-small cell lung cancer	0.009	0.008	0.549	0.980	1.096	Braf;Alk
path:mmu00230	Purine metabolism	0.022	0.022	0.550	0.980	1.016	Pola1;Polr3k;Pklr;Aprt;Polr1d
path:mmu04910	Insulin signaling pathway	0.018	0.017	0.551	0.980	1.026	Braf;Cblb;Pklr;Rheb
path:mmu04064	NF-kappa B signaling pathway	0.013	0.013	0.553	0.980	1.043	Malt1;Tnfsf13b;Vcam1
path:mmu00010	Glycolysis / Gluconeogenesis	0.009	0.008	0.557	0.980	1.079	Pklr;Pdha2
path:mmu04720	Long-term potentiation	0.009	0.008	0.557	0.980	1.079	Braf;Atf4
path:mmu05146	Amoebiasis	0.013	0.013	0.566	0.980	1.023	Il12a;Serpinb6c;Serpinb9

path:mmu04010	MAPK signaling pathway	0.036	0.036	0.566	0.980	0.987	Atf4;Dusp5;Dusp1;Hspa8;Braf;Ppm1a;Fasl;Myc
path:mmu04710	Circadian rhythm	0.004	0.004	0.581	0.980	1.166	Creb1
path:mmu04931	Insulin resistance	0.013	0.013	0.585	0.980	0.995	Creb1;Slc27a2;Creb5
path:mmu04215	Apoptosis	0.004	0.004	0.593	0.980	1.130	43712
path:mmu00052	Galactose metabolism	0.004	0.004	0.593	0.980	1.130	Ganc
path:mmu05412	Arrhythmogenic right ventricular cardiomyopathy (ARVC)	0.009	0.009	0.597	0.980	1.004	Actn2;Gja1
path:mmu04662	B cell receptor signaling pathway	0.009	0.009	0.597	0.980	1.004	Malt1;Nfkbie
path:mmu04924	Renin secretion	0.009	0.009	0.597	0.980	1.004	Creb1;Adcyap1r1
path:mmu00500	Starch and sucrose metabolism	0.004	0.004	0.604	0.980	1.096	Ganc
path:mmu05224	Breast cancer	0.018	0.019	0.605	0.980	0.958	Braf;Notch4;Pgr;Myc
path:mmu04932	Non-alcoholic fatty liver disease (NAFLD)	0.018	0.019	0.605	0.980	0.958	Fasl;Lepr;Pklr;Atf4
path:mmu05020	Prion diseases	0.004	0.004	0.615	0.980	1.064	Stip1
path:mmu05212	Pancreatic cancer	0.009	0.009	0.619	0.980	0.964	Braf;Ralgds
path:mmu04670	Leukocyte transendothelial migration	0.013	0.014	0.621	0.980	0.943	Arhgap5;Cldn4;Vcam1
path:mmu03410	Base excision repair	0.004	0.004	0.626	0.980	1.033	Neil2
path:mmu00051	Fructose and mannose metabolism	0.004	0.004	0.626	0.980	1.033	Fpgt
path:mmu03030	DNA replication	0.004	0.004	0.626	0.980	1.033	Pola1
path:mmu01524	Platinum drug resistance	0.009	0.009	0.626	0.980	0.952	Fasl;Gsta3
path:mmu05100	Bacterial invasion of epithelial cells	0.009	0.009	0.626	0.980	0.952	Cblb;Sept1
path:mmu05340	Primary immunodeficiency	0.004	0.004	0.636	0.980	1.004	Rfxap
path:mmu05165	Human papillomavirus infection	0.040	0.043	0.637	0.980	0.930	Creb1;H2-M9;Rbpjl;Ube3a;Rheb;Creb5;Ccnd2;Notch4;Fasl
path:mmu04650	Natural killer cell mediated cytotoxicity	0.013	0.015	0.639	0.980	0.919	Braf;Fasl;Raet1b
path:mmu05320	Autoimmune thyroid disease	0.009	0.010	0.647	0.980	0.915	H2-M9;Fasl
path:mmu04151	PI3K-Akt signaling pathway	0.040	0.044	0.654	0.980	0.917	Creb1;Mtcp1;Hsp90aa1;Atf4;Rheb;Creb5;Ccnd2;Fasl;Myc
path:mmu00350	Tyrosine metabolism	0.004	0.005	0.666	0.980	0.927	Mif
path:mmu04110	Cell cycle	0.013	0.015	0.672	0.980	0.875	Anapc10;Myc;Ccnd2
path:mmu05323	Rheumatoid arthritis	0.009	0.010	0.674	0.980	0.871	Tnfsf13b;Atp6v1h
path:mmu04975	Fat digestion and absorption	0.004	0.005	0.675	0.980	0.904	Pla2g5
path:mmu00260	Glycine, serine and threonine metabolism	0.004	0.005	0.675	0.980	0.904	Cth
path:mmu05033	Nicotine addiction	0.004	0.005	0.675	0.980	0.904	Chrnb2

path:mmu05205	Proteoglycans in cancer	0.022	0.025	0.679	0.980	0.878	Ptch1;Cblb;Braf;Fasl;Myc
path:mmu04080	Neuroactive ligand-receptor interaction	0.031	0.035	0.679	0.980	0.888	Htr6;Thra;Lepr;Cysltr2;Gpr83;Chrnb2;Adcyap1r1
path:mmu04216	Ferroptosis	0.004	0.005	0.684	0.980	0.882	Acsl3
path:mmu04022	cGMP-PKG signaling pathway	0.018	0.021	0.688	0.980	0.861	Creb1;Creb5;Prkg1;Atf4
path:mmu04540	Gap junction	0.009	0.011	0.693	0.980	0.841	Prkg1;Gja1
path:mmu05225	Hepatocellular carcinoma	0.018	0.021	0.697	0.980	0.851	Braf;Nfe2l2;Gsta3;Myc
path:mmu04140	Autophagy	0.013	0.016	0.703	0.980	0.834	Rb1cc1;Uvrag;Rheb
path:mmu05416	Viral myocarditis	0.009	0.011	0.705	0.980	0.822	H2-M9;Cd55
path:mmu04926	Relaxin signaling pathway	0.013	0.016	0.708	0.980	0.828	Creb1;Creb5;Atf4
path:mmu03420	Nucleotide excision repair	0.004	0.005	0.710	0.980	0.822	Gtf2h1
path:mmu05168	Herpes simplex infection	0.022	0.026	0.715	0.980	0.841	H2-M9;Il12a;Fasl;Sp100;Taf9b
path:mmu04728	Dopaminergic synapse	0.013	0.016	0.722	0.980	0.810	Creb1;Creb5;Atf4
path:mmu04657	IL-17 signaling pathway	0.009	0.011	0.722	0.980	0.795	Fosl1;Hsp90aa1
path:mmu01522	Endocrine resistance	0.009	0.011	0.733	0.980	0.778	Braf;Notch4
path:mmu04550	Signaling pathways regulating pluripotency of stem cells	0.013	0.017	0.736	0.980	0.792	Hesx1;Myc;Bmi1
path:mmu04930	Type II diabetes mellitus	0.004	0.006	0.741	0.980	0.753	Pklr
path:mmu04640	Hematopoietic cell lineage	0.009	0.012	0.744	0.980	0.761	Cd55;Cd24a
path:mmu00510	N-Glycan biosynthesis	0.004	0.006	0.748	0.980	0.738	St6gal2
path:mmu04979	Cholesterol metabolism	0.004	0.006	0.748	0.980	0.738	Apoc3
path:mmu00071	Fatty acid degradation	0.004	0.006	0.755	0.980	0.723	Acsl3
path:mmu04218	Cellular senescence	0.018	0.023	0.762	0.980	0.778	H2-M9;Myc;Ccnd2;Rheb
path:mmu00330	Arginine and proline metabolism	0.004	0.006	0.762	0.980	0.709	Ckb
path:mmu01212	Fatty acid metabolism	0.004	0.006	0.768	0.980	0.695	Acsl3
path:mmu05142	Chagas disease (American trypanosomiasis)	0.009	0.012	0.774	0.980	0.716	Il12a;Fasl
path:mmu04072	Phospholipase D signaling pathway	0.013	0.018	0.778	0.980	0.738	Dgki;Ralgds;Rheb
path:mmu04261	Adrenergic signaling in cardiomyocytes	0.013	0.018	0.778	0.980	0.738	Creb1;Creb5;Atf4
path:mmu04923	Regulation of lipolysis in adipocytes	0.004	0.007	0.787	0.980	0.657	Prkg1
path:mmu05016	Huntington's disease	0.018	0.024	0.790	0.980	0.746	Creb1;Dnah17;Creb5;Dnah2
path:mmu04150	mTOR signaling pathway	0.013	0.019	0.801	0.980	0.709	Braf;Atp6v1h;Rheb
path:mmu04390	Hippo signaling pathway	0.013	0.019	0.804	0.980	0.704	Amot;Myc;Ccnd2

path:mmu05145	Toxoplasmosis	0.009	0.013	0.805	0.980	0.670	Il12a;Hspa8
path:mmu05034	Alcoholism	0.018	0.024	0.806	0.980	0.727	Braf;Creb1;Creb5;Atf4
path:mmu04510	Focal adhesion	0.018	0.024	0.806	0.980	0.727	Braf;Arhgap5;Parvb;Ccnd2
path:mmu00561	Glycerolipid metabolism	0.004	0.007	0.820	0.980	0.593	Dgki
path:mmu04213	Longevity regulating pathway	0.004	0.008	0.825	0.980	0.583	Hspa8
path:mmu04137	Mitophagy	0.004	0.008	0.830	0.980	0.574	Atf4
path:mmu05217	Basal cell carcinoma	0.004	0.008	0.830	0.980	0.574	Ptch1
path:mmu04114	Oocyte meiosis	0.009	0.014	0.836	0.980	0.623	Anapc10;Pgr
path:mmu04530	Tight junction	0.013	0.021	0.846	0.980	0.650	Amot;Mpp4;Cldn4
path:mmu05140	Leishmaniasis	0.004	0.008	0.848	0.980	0.540	Il12a
path:mmu05164	Influenza A	0.013	0.021	0.849	0.980	0.646	Il12a;Fasl;Hspa8
path:mmu04514	Cell adhesion molecules (CAMs)	0.013	0.021	0.852	0.980	0.642	H2-M9;Cldn4;Vcam1
path:mmu04622	RIG-I-like receptor signaling pathway	0.004	0.008	0.853	0.980	0.532	Il12a
path:mmu05167	Kaposi's sarcoma-associated herpesvirus infection	0.018	0.027	0.856	0.980	0.667	Creb1;H2-M9;Myc;Ccr8
path:mmu05211	Renal cell carcinoma	0.004	0.008	0.857	0.980	0.524	Braf
path:mmu04611	Platelet activation	0.009	0.015	0.862	0.980	0.583	Prkg1;Tbxas1
path:mmu05214	Glioma	0.004	0.009	0.865	0.980	0.509	Braf
path:mmu04520	Adherens junction	0.004	0.009	0.868	0.980	0.502	Lmo7
path:mmu04917	Prolactin signaling pathway	0.004	0.009	0.868	0.980	0.502	Ccnd2
path:mmu04380	Osteoclast differentiation	0.009	0.016	0.874	0.980	0.565	Creb1;Fosl1
path:mmu05133	Pertussis	0.004	0.009	0.883	0.980	0.476	Il12a
path:mmu05218	Melanoma	0.004	0.009	0.883	0.980	0.476	Braf
path:mmu04970	Salivary secretion	0.004	0.010	0.889	0.980	0.464	Prkg1
path:mmu05132	Salmonella infection	0.004	0.010	0.889	0.980	0.464	Dync2h1
path:mmu00190	Oxidative phosphorylation	0.009	0.016	0.890	0.980	0.540	Cox10;Atp6v1h
path:mmu05160	Hepatitis C	0.009	0.016	0.890	0.980	0.540	Braf;Cldn4
path:mmu05206	MicroRNAs in cancer	0.022	0.035	0.894	0.980	0.643	Ccng1;Ccnd2;Notch4;Myc;Bmi1
path:mmu01521	EGFR tyrosine kinase inhibitor resistance	0.004	0.010	0.895	0.980	0.452	Braf
path:mmu04014	Ras signaling pathway	0.018	0.029	0.899	0.980	0.610	Ralgds;Fasl;Pla2g5;Pla2g6
path:mmu04350	TGF-beta signaling pathway	0.004	0.010	0.909	0.980	0.425	Myc

path:mmu04062	Chemokine signaling pathway	0.013	0.024	0.911	0.980	0.556	Braf;Cx3cr1;Ccr8
path:mmu04666	Fc gamma R-mediated phagocytosis	0.004	0.011	0.914	0.980	0.416	Pla2g6
path:mmu04610	Complement and coagulation cascades	0.004	0.011	0.916	0.980	0.411	Cd55
path:mmu04912	GnRH signaling pathway	0.004	0.011	0.919	0.980	0.406	Atf4
path:mmu05222	Small cell lung cancer	0.004	0.011	0.925	0.980	0.393	Myc
path:mmu05226	Gastric cancer	0.009	0.019	0.930	0.980	0.470	Braf;Myc
path:mmu04070	Phosphatidylinositol signaling system	0.004	0.012	0.937	0.980	0.369	Dgki
path:mmu04015	Rap1 signaling pathway	0.013	0.026	0.938	0.980	0.509	Braf;Ralgds;Magi2
path:mmu04620	Toll-like receptor signaling pathway	0.004	0.012	0.939	0.980	0.365	Il12a
path:mmu04916	Melanogenesis	0.004	0.012	0.941	0.980	0.362	Creb1
path:mmu04933	AGE-RAGE signaling pathway in diabetic complications	0.004	0.012	0.941	0.980	0.362	Vcam1
path:mmu04621	NOD-like receptor signaling pathway	0.009	0.021	0.950	0.980	0.430	Dhx33;Hsp90aa1
path:mmu04066	HIF-1 signaling pathway	0.004	0.013	0.950	0.980	0.341	Pdha2
path:mmu05010	Alzheimer's disease	0.009	0.022	0.957	0.980	0.413	Aph1b;Aph1c
path:mmu04217	Necroptosis	0.009	0.022	0.959	0.980	0.409	Fasl;Hsp90aa1
path:mmu04724	Glutamatergic synapse	0.004	0.014	0.960	0.980	0.317	Trpc1
path:mmu04020	Calcium signaling pathway	0.009	0.022	0.965	0.981	0.395	Cysltr2;Htr6
path:mmu04750	Inflammatory mediator regulation of TRP channels	0.004	0.015	0.971	0.983	0.289	Pla2g6
path:mmu04371	Apelin signaling pathway	0.004	0.017	0.980	0.989	0.260	Klf2
path:mmu04740	Olfactory transduction	0.084	0.138	0.995	0.998	0.612	Olfr320; Olfr706; Olfr700;Olfr693;Olfr524;Olfr560;Olfr358;Olfr391ps; Olfr870; Olfr139;Olfr736;Olfr1306;Olfr156;Olfr1170;Olfr502;Prkg1;Olfr103; Olfr340;Olfr1226
path:mmu04810	Regulation of actin cytoskeleton	0.004	0.026	0.998	0.998	0.169	Braf

Table S3. Go enrichment of the differentially expressed proteins in aortas from CIH-treated mice.

Term_ID	Term description	Bg Ratio	P_value	Category	Fold	GeneSymbols
GO:0006355	regulation of transcription, DNA-templated	8.81E-02	1.06E-07	Process	1.72E+00	Gtf2a1;Rbpjl;Dlx6;Rbak;Zfx;Rnf2;Klf16;Npat;Zbtb46;Mllt3;Rrn3;Trim33;Rfx6;Thra;Pou6f1;Rb1cc1;Myf6;Ctnnd2;Zfp37;Nfyb;Nsd1;Tal1;Creb5;Klf2;EU599041;Kdm4c;Adnp2;Myc;Ccnc;Zfp329;Ctr9;Onecut2;Ctcf;Gm14409;Zfp229;Cep290;Zfp72;Zfp131;Med19;Safb2;Ikzf5;Zfp62;Irf6;Esf1;Zfp874a;Zfp521;Gtf2h1;Zfp119b;Sox14;Zfp518a;Ybx1;Churc1;Fasl;Kmt5b;Creb1;Pgr;Taf1b;Zfp53;Mphosph8;Bsx;Nfe2l2;Elp4;Vgll2;Pspc1;Hspa8;Hesx1;Notch4;Eaf1;Prop1;Atf4;Gpbp1;Zfp60;Itgb3bp;Khdrbs2;Ezh1;Zfp654;Btf3;Myt1;Taf9b;Tle3;Fosl1;Cited1;Zfp281;2610044O15Rik8;Brd8;Bmi1;Igsf1;Nol11;Prmt3;Cdyl;Cbx7;2410141K09Rik;Ikzf3;Zfp780b;Zfp322a;Sp100
GO:0006351	transcription, DNA-templated	7.89E-02	3.35E-06	Process	1.66E+00	Gtf2a1;Rbpjl;Ccnc;Polr3k;Btf3;Sox14;Zfp518a;Ybx1;Churc1;Rbak;Myt1;Zfp329;Zfx;Rnf2;Ctr9;Fasl;Klf16;Onecut2;Kmt5b;Taf9b;Creb1;Tle3;Sp100;Pgr;Ctcf;Adnp2;Cited1;Zfp281;Npat;Swt1;Taf1b;Mphosph8;Brd8;Zbtb46;Bsx;Cep290;Zfp131;Nfe2l2;Med19;Mllt3;Rrn3;Trim33;Bmi1;Rfx6;Thra;Ikzf5;Safb2;Nol11;Zfp62;Pou6f1;Rb1cc1;Elp4;Vgll2;Fubp3;Irf6;Esf1;Pspc1;Ctnnd2;Hesx1;Cdyl;Hspa8;Zfp37;Eaf1;Notch4;Cbx7;Nfyb;Nsd1;Tal1;Atf4;Ikzf3;Creb5;Gpbp1;Klf2;Kdm4c;Itgb3bp;Zfp521;Gtf2h1;Khdrbs2;Zfp322a;Ezh1;Zfp654;Polr1d;Myc

GO:0005634	nucleus	2.48E-01	7.35E-06	Component	1.30E+00	Gtf2a1;Rbpjl;Dlx6;Lamp3;Fam206a;Rbak;Azin1;Zfx;Rnf2;Rbm22;Crnkl1;Ppm1a;Klf16;Tax1bp3;Ube2w;Zscan5b;Tekt5;Npat;Rfxap;Top3a;Zbtb46;Mllt3;Rrn3;Bex4;Trim33;Rfx6;Thra;Ube3a;Luc7l2;Pou6f1;Sf3a3;Sesn3;Rb1cc1;Dhx33;Myf6;Lmo2;Ctnnd2;Ugdh;Dcps;Zfp37;Pygo1;Nfyb;Nsd1;Tal1;Ncbp2;Creb5;Exosc3;Klf2;Herc6;Kdm4c;Nckipsd;4933402N03Rik;Bag3;Blm;Fubp3;Vps37a;Cul5;Myc;Ppwd1;Lsm5;Adnp2;Ccnc;Acd;Zfp329;Txndc9;Ralgapa2;Ctr9;Onecut2;Crbn;Pdcl3;Ctcf;Dyrk1a;N4bp2l2;Ube2c;Stip1;Plpp7;Cep290;Zfp72;Zfp131;Med19;Tex10;Senp7;Safb2;Ikzf5;Pola1;Zfp62;Ralgds;Cblb;Cps1;Irf6;Esf1;Padi6;Ing1;Igf2bp3;Lemd3;Ubqln1;Dusp1;Anp32e;Zfp521;Gtf2h1;Miat;Braf;Ybey;Mettl3;Strbp;Polr3k;Zfp119b;Toe1;Ptch1;Sox14;Zfp518a;Ybx1;Churc1;Zfp438;Fasl;Kmt5b;Creb1;Pgr;Rev1;Hmgn2;Taf1b;Mphosph8;Bsx;Sf3b6;Nfe2l2;Kpnb1;Mif;Cacybp;Malt1;Thoc2;Elp4;Vgll2;Brix1;Pym1;Mpo;Pspc1;Hspa8;Ipo7;Tc2n;Ckb;Hesx1;Eaf1;Notch4;Prop1;Zfp106;Wtap;Neil2;Atf4;Gpbp1;G2e3;Ccng1;Umps;Zfp60;Esco1;Dgki;Itgb3bp;Magohb;Khdrbs2;Ezh1;Zfp654;Ssfa2;Naa50;Btf3;Vldlr;Nfkbie;Mcm8;Gtf3c3;Pus7;Gnpda2;Cep350;Magi2;Ccnd2;Myt1;Nol10;Morn2;Uba3;Taf9b;Sde2;Tle3;Fosl1;Cited1;Lsg1;Zfp281;Swt1;Brd8;Bmi1;Appbp2;Gm5415;Enoph1;Ifrd2;Nol11;Arhgap27;Krr1;Gar1;Cdyl;Cmas;Cbx7;Hsp90aa1;Aurkb;Ikzf3;Nup37;Serpinb9;Prkx;Zfp322a;Cecr2;Polr1d;Wrn;Sp100;Rbm8a;Kpna4
GO:0051782	negative regulation of cell division	2.97E-04	1.69E-05	Process	2.13E+01	Ptch1;Intu;Myc;Blm
GO:0003676	nucleic acid binding	3.64E-02	8.26E-05	Function	1.85E+00	Polr3k;Toe1;Zfp518a;Ybx1;Rbak;Zfp329;Zfx;Rbm22;Klf16;Eea1;Ctcf;Zfp281;Top3a;Zbtb46;Sf3b6;Zfp131;Safb2;Ikzf5;Pola1;Zfp62;Sf3a3;Zcchc2;Dhx33;Zcchc10;Krr1;Rbsn;Pspc1;Zfp37;Igf2bp3;Lemd3;Zfp106;Neil2;Ncbp2;Ikzf3;Zfp60;Klf2;Zfp521;Zfp322a;Khdrbs2;Blm;Wrn;Zfp654;Adnp2
GO:0061077	chaperone-mediated protein folding	1.86E-03	1.58E-04	Process	5.92E+00	Cct6a;Cct7;Cct4;Hspd1;Hspa8;Chordc1;Canx

GO:0005515	protein binding	2.09E-01	2.06E-04	Function	1.28E+00	Gtf2a1;Cct7;Tff1;Rnf2;Sfrp1;Tax1bp3;Lin7c;Ube2w;Pkd2;Epha5;Zbtb46;Mllt3;Trim33;Rfx6;Sema3a;Thra;Ube3a;Rb1cc1;Sesn3;Tada2b;Lmo2;Capza2;Sptbn4;Pygo1;Ccr8;Anapc10;Nsd1;Slc7a9;Tal1;Btg3;Klf2;Bag3;Stap2;Blm;Prkg1;Slc5a2;Cul5;Myc;Lsm5;Chrnb2;Htr6;Alk;Acd;Cct4;Ctr9;Ctcf;Dyrk1a;N4bp2l2;Hpca;Stip1;Nup153;Cep290;Bbs9;Med19;Senp7;Kctd12;Pola1;Gstk1;Ralgds;Cblb;Cps1;Padi6;Ccm2;Igf2bp3;Tspan13;Cd96;Raet1b;Miat;Braf;Khdc1b;Cct6a;Mettl3;Ptch1;Sema4g;Ybx1;Ppfia4;Reep5;Il12a;Canx;Creb1;Rpl39;Pgr;Rev1;Taf1b;Zfp53;Ift81;Csf2rb;Bsx;Nfe2l2;Exoc3l;Kpnb1;Parvb;Gja1;Cacybp;Malt1;Thoc2;Vgll2;Usp33;Stoml3;Pspc1;Hspa8;Ipo7;Hesx1;Ckb;Myo16;Notch4;Prop1;Il12rb1;Wtap;Dhh;Cd24a;Atf4;Scn8a;Gpbp1;Tmem178;Trpc1;Khdrbs2;Cit;Ezh1;Cldn4;Cdh23;Naa50;Lepr;Nfkbie;Siglecg;Vldlr;Cep350;Magi2;Ccnd2;Myt1;Dnah17;Uba3;Adam5;Eea1;Tle3;Cep55;Tom1l1;Cited1;Zfp281;Tmem108;Actn2;Trip13;Adcyap1r1;Amot;Appbp2;Bmi1;Trim38;Uvrag;Spatc1;Map2;Prmt3;Snx1;Gar1;Dusp19;Fndc5;Slc9c1;Cbx7;Bfsp2;Hsp90aa1;Aurkb;Ikzf3;Cth;Mpv17l;Plxdc1;Serpinb9;Wrn;Polr1d;Kpna4;Sp100;Chordc1
GO:0005540	hyaluronic acid binding	1.00E-03	3.59E-04	Function	7.84E+00	Stab1;Impg2;Tnfaip6;Hapln4;Stab2
GO:0006458	'de novo' protein folding	5.93E-04	4.17E-04	Process	1.06E+01	Cct6a;Hspd1;Cct4;Cct7
GO:0060509	Type I pneumocyte differentiation	2.97E-04	6.23E-04	Process	1.60E+01	Creb1;Thra;Klf2
GO:0044183	protein binding involved in protein folding	1.13E-03	6.54E-04	Function	6.94E+00	Cct6a;Pdcl3;Cct7;Cct4;Hspd1
GO:0072341	modified amino acid binding	3.05E-04	6.83E-04	Function	1.55E+01	Cps1;Mut;Prmt3
GO:0045893	positive regulation of transcription, DNA-templated	2.53E-02	8.21E-04	Process	1.87E+00	Rbpjl;Ptch1;Churc1;Sfrp1;Ppm1a;Creb1;Pgr;Ctcf;Cited1;Zfp281;Npat;Rfxap;Cep290;Nfe2l2;Mllt3;Rrn3;Rfx6;Myf6;Irf6;Ing1;Nfyb;Nsd1;Tal1;Atf4;Creb5;Gpbp1;Klf2;Blm;Fubp3;Myc
GO:1904871	positive regulation of protein localization to Cajal body	3.39E-04	9.77E-04	Process	1.40E+01	Cct6a;Cct4;Cct7

GO:0005929	cilium	1.13E-02	1.26E-03	Component	2.31E+00	Htr6;Ptch1;Pkd2;Lca5;Ttll9;Dync2h1;Stoml3;Dnah17;Tulp4;Dnah2;Cep290;Cct4;Bbs9;Slc9c1;Rp1;Ift81;Umod
GO:0050667	homocysteine metabolic process	3.81E-04	1.44E-03	Process	1.24E+01	Cth;Cps1;Mut
GO:1904851	positive regulation of establishment of protein localization to telomere	3.81E-04	1.44E-03	Process	1.24E+01	Cct6a;Cct4;Cct7
GO:0045292	mRNA cis splicing, via spliceosome	3.81E-04	1.44E-03	Process	1.24E+01	Dcps;Rbm22;Ncbp2
GO:0042026	protein refolding	3.81E-04	1.44E-03	Process	1.24E+01	Hsp90aa1;Hspd1;Hspa8
GO:0046850	regulation of bone remodeling	3.81E-04	1.44E-03	Process	1.24E+01	Lepr;Suco;Gja1
GO:0030644	cellular chloride ion homeostasis	3.81E-04	1.44E-03	Process	1.24E+01	Ckb;Fasl;Tbxas1
GO:0032212	positive regulation of telomere maintenance via telomerase	1.36E-03	1.52E-03	Process	5.82E+00	Cct6a;Aurkb;Cct7;Acd;Cct4
GO:0000184	nuclear-transcribed mRNA catabolic process, nonsense-mediated decay	1.36E-03	1.52E-03	Process	5.82E+00	Ncbp2;Gspt1;Magohb;Pym1;Rbm8a
GO:0051082	unfolded protein binding	3.49E-03	1.68E-03	Function	3.61E+00	Cct6a;Hsp90aa1;Cct7;Hspe1;Cct4;Hspa8;Hspd1;Canx
GO:0044297	cell body	4.22E-03	1.69E-03	Component	3.29E+00	Cct6a;Cct7;Cacybp;Map2;Alk;Usp33;Cct4;Rdh10;Braf
GO:0051973	positive regulation of telomerase activity	1.40E-03	1.75E-03	Process	5.64E+00	Hsp90aa1;Aurkb;Acd;Cct4;Myc

GO:0005737	cytoplasm	2.67E-01	2.11E-03	Component	1.19E+00	Gtf2a1;Cct7;Ttll9;Dennd6a;Azin1;Tff1;Rbm22;Ppm1a;Tax1bp3;Stab1;Lin7c;Serpinb6c;Intu;Pkd2;Slc23a3;Ube2w;Npat;Tnfsf13b;Bex4;Thra;Vash1;Ube3a;Rb1cc1;Dhx33;Sesn3;Snx16;Sbk1;Sptbn4;Dcps;Dync2h1;Fbxl14;Dennd2d;Anapc10;Ncbp2;Btg3;Exosc3;Herc6;Nyx;Bag3;Stap2;Blm;Clmn;Prkg1;Fubp3;Cul5;Myc;Lsm5;Fabp6;Amdhd1;Arhgap5;Idh1;Hspe1;Alox12b;Txndc9;Ralgapa2;Cct4;Ppp6r2;Rp1;Crbn;Cep112;Ttc7;Pdcl3;Aprt;Hpca;Ube2c;Cfap61;Stip1;Cep57l1;Rdh10;Cep290;Bbs9;Tex10;Cst13;Eml6;Safb2;Pola1;Pla2g6;Ralgds;Cblb;Cps1;Irf6;Padi6;Stab2;Ccm2;Igf2bp3;Cd96;Ubqln1;Dusp1;Anp32e;Braf;Txndc2;Khdc1b;Cct6a;Mettl3;Strbp;Pdlim2;Pklr;Toe1;Ppih;Ybx1;Riok1;Scel;Il12a;Arhgef16;Canx;Mpp4;Pgr;2310050C09Rik;Mphosph8;Bsx;Ythdf1;Nfe2l2;Kpnb1;Mif;Parvb;Gja1;Cacybp;Malt1;Elp4;Vgll2;Usp33;Pym1;Pspc1;Hspa8;Ipo7;Shpk;Ckb;Fam126a;Myo16;Tulp4;Zfp106;Angel2;Neil2;Atf4;Copg1;G2e3;Umps;Zfp185;Sprr4;Dgki;Trpc1;Fgd4;Cit;Dnah2;Ssfa2;Naa50;Btf3;Nfkbie;Gnpda2;Cep350;Magi2;Ccnd2;Pex1;Dnah17;Uba3;Fpgt;Eea1;Agbl3;Cep55;Tom1l1;Cited1;Lsg1;Npl;Actn2;Sept1;Hspd1;Adcyap1r1;Amot;Appbp2;Sept4;Bmi1;Gsta3;Enoph1;Trim13;Spatc1;Zcchc2;Map2;Prmt3;Chil6;Arhgap27;Snx1;Krr1;Sgsm2;Dusp19;Bgn;Bfsp2;Sept10;Hsp90aa1;Aurkb;Lca5;Hrasls;Rheb;Cth;Ikzf3;Plxdc1;Mpv17l;Serpinb9;Ppp1r2;Cplx2;Prkx;Zfp322a;Wrn;Kpna4;Sp100;Zfand5;Trappc2l
GO:0061436	establishment of skin barrier	8.90E-04	2.15E-03	Process	7.09E+00	Flg;Alox12b;Cldn4;Cela2a
GO:0046872	metal ion binding	1.31E-01	2.33E-03	Function	1.30E+00	Rbak;Zfx;Rnf2;Rbm22;Ppm1a;Klf16;Rnf139;Pkd2;Adam26a;Top3a;Zbtb46;Fstl5;Trim33;Thra;Ube3a;Sf3a3;Lmo2;Zfp37;Pygo1;Nsd1;Adamts20;Klf2;Kdm4c;Blm;Adnp2;Amdhd1;Idh1;Hspe1;Alox12b;Zfp329;Crbn;Bpnt1;Ctcf;Hpca;Zfp131;Tbxas1;Mob3c;Ikzf5;Pola1;Zfp62;Rptn;Zc2hc1a;Rspry1;Cblb;Cps1;Ing1;Timm9;Zfp521;Braf;Ybey;Pdlim2;Pklr;Polr3k;Mmp10;Toe1;Zfp518a;Churc1;Riok1;Scel;Canx;Pon1;Pgr;Rev1;Taf1b;Usp33;Rbsn;Mpo;Zbtb8os;Zfp106;Dhh;Neil2;G2e3;Zfp60;Esco1;Zfp185;Rnf103;Atp2c1;Fgd4;Cit;Zfp654;Cyp2f2;Cdh23;Myt1;Eea1;Agbl3;Mcee;Zfp281;Zc3h12c;Actn2;Col11a1;Slc25a25;Bmi1;Enoph1;Atp11c;Trim13;Zcchc2;Pla2g5;Zcchc10;Prmt3;Mut;Atp5s;Aurkb;Rheb;Ikzf3;Zfp322a;Wrn;Zfand5;Chordc1
GO:0034399	nuclear periphery	8.95E-04	2.38E-03	Component	6.89E+00	Nup153;Kpnb1;Map2;Atf4
GO:0005791	rough endoplasmic reticulum	2.85E-03	2.47E-03	Component	3.78E+00	Suco;Map2;Epha5;Canx;Myc;Hspd1;Adcyap1r1
GO:0045333	cellular respiration	9.32E-04	2.57E-03	Process	6.77E+00	Coq10b;Slc25a25;Cox10;Sdhaf4

GO:0007601	visual perception	5.51E-03	2.72E-03	Process	2.86E+00	Bfsp2;Impg2;Rgr;Sox14;Opn4;Nyx;Col11a1;Rdh10;Rp1;Chrnb2
GO:0005832	chaperonin-containing T-complex	4.69E-04	2.94E-03	Component	9.86E+00	Cct6a;Cct4;Cct7
GO:0030879	mammary gland development	1.61E-03	3.32E-03	Process	4.90E+00	Creb1;Arhgap5;Ptch1;Pgr;Notch4
GO:0071498	cellular response to fluid shear stress	5.08E-04	3.54E-03	Process	9.31E+00	Nfe2l2;Klf2;Pkd2
GO:0008152	metabolic process	1.84E-02	3.56E-03	Process	1.89E+00	Pklr;Ctbs;Enpp6;Glb1l2;Ganc;Npl;Enoph1;Pla2g6;Gsta3;Cps1;Ugt2a3;Ugdh;Cdyl;Mut;Slc27a2;Neil2;Umps;Acsl3;Auh;Wrn;Prkg1;Pdha2
GO:0006457	protein folding	4.03E-03	4.07E-03	Process	3.13E+00	Cct6a;Hsp90aa1;Cct7;Hspe1;Cct4;Hspa8;Hspd1;Canx
GO:0042254	ribosome biogenesis	4.07E-03	4.34E-03	Process	3.10E+00	Nol11;Lsg1;Riok1;Brix1;Krr1;Gar1;Tsr3;Rrn3
GO:0005777	peroxisome	5.75E-03	4.36E-03	Component	2.68E+00	Ddo;Slc27a2;Idh1;Gstk1;Hrasls;Fndc5;Mpv17l;Pex1;Acsl3;Acnat2
GO:0048699	generation of neurons	5.51E-04	4.51E-03	Process	8.59E+00	Cit;Thoc2;Tal1
GO:0045943	positive regulation of transcription from RNA polymerase I promoter	5.51E-04	4.51E-03	Process	8.59E+00	Dhx33;Heatr1;Atf4
GO:0060674	placenta blood vessel development	5.51E-04	4.51E-03	Process	8.59E+00	Fosl1;Vash1;Pkd2
GO:0002199	zona pellucida receptor complex	5.54E-04	4.89E-03	Component	8.35E+00	Cct6a;Cct4;Cct7
GO:0016485	protein processing	2.54E-03	5.38E-03	Process	3.72E+00	Aph1b;Pcsk1;Ptch1;Aph1c;Dync2h1;Myc
GO:0001702	gastrulation with mouth forming second	1.14E-03	5.54E-03	Process	5.51E+00	Amot;Ugdh;Nsd1;Rnf2

GO:0000974	Prp19 complex	5.96E-04	6.10E-03	Component	7.75E+00	Crnkl1;Rbm22;Hspa8
GO:0030133	transport vesicle	1.83E-03	6.43E-03	Component	4.21E+00	Aph1b;Pigr;Atp2c1;Tmem168;Bgn
GO:0007028	cytoplasm organization	2.12E-04	6.83E-03	Process	1.49E+01	Padi6;Rrn3
GO:0036003	positive regulation of transcription from RNA polymerase II promoter in response to stress	2.12E-04	6.83E-03	Process	1.49E+01	Nfe2l2;Klf2
GO:0002904	positive regulation of B cell apoptotic process	2.12E-04	6.83E-03	Process	1.49E+01	Cd24a;Myc
GO:0090238	positive regulation of arachidonic acid secretion	2.12E-04	6.83E-03	Process	1.49E+01	Mif;Pla2g6
GO:0008616	queuosine biosynthetic process	2.12E-04	6.83E-03	Process	1.49E+01	Txndc9;Pdcl3
GO:0006360	transcription from RNA polymerase I promoter	2.12E-04	6.83E-03	Process	1.49E+01	Taf1b;Gtf2h1
GO:1904874	positive regulation of telomerase RNA localization to Cajal body	6.36E-04	6.90E-03	Process	7.44E+00	Cct6a;Cct4;Cct7
GO:0030890	positive regulation of B cell proliferation	1.91E-03	6.93E-03	Process	4.14E+00	Mif;Gpr183;Tnfsf13b;Chrnb2;Bmi1
GO:0042113	B cell activation	1.23E-03	7.19E-03	Process	5.13E+00	Malt1;Hspd1;Chrnb2;Ikzf3
GO:0009378	four-way junction helicase activity	2.18E-04	7.26E-03	Function	1.44E+01	Wrn;Blm

GO:0000405	bubble DNA binding	2.18E-04	7.26E-03	Function	1.44E+01	Wrn;Blm
GO:0034185	apolipoprotein binding	6.54E-04	7.53E-03	Function	7.21E+00	Vldlr;Hspd1;Canx
GO:0003700	DNA binding transcription factor activity	3.71E-02	8.29E-03	Function	1.53E+00	Rbpjl;Myt1;Zfp438;Klf16;Onecut2;Creb1;Fosl1;Pgr;Ctcf;Cited1;Zfp281;2610044O15Rik8;Gm14409;Zfp229;Zfp53;Bsx;Zfp72;Nfe2l2;Rfx6;Thra;Pou6f1;Tada2b;Irf6;Nfyb;Zfp874a;2410141K09Rik;Tal1;Atf4;Creb5;Gpbp1;Zfp60;Klf2;EU599041;Zfp780b;Zfp322a;Myc
GO:0017124	SH3 domain binding	5.49E-03	8.55E-03	Function	2.58E+00	Zfp106;Uvrag;Tom1l1;Gja1;Nckipsd;Cblb;Arhgap27;Khdrbs2;Cit
GO:0051371	muscle alpha-actinin binding	6.98E-04	9.08E-03	Function	6.76E+00	Mybpc2;Pdlim2;Pkd2
GO:0006606	protein import into nucleus	2.88E-03	9.83E-03	Process	3.28E+00	Nup153;Hspa8;Ipo7;Kpnb1;Kpna4;Ing1
GO:0030490	maturation of SSU-rRNA	7.20E-04	9.91E-03	Process	6.57E+00	Tsr3;Nol11;Riok1
GO:0031396	regulation of protein ubiquitination	7.20E-04	9.91E-03	Process	6.57E+00	Ubqln1;Hsp90aa1;Rnf139
GO:0006352	DNA-templated transcription, initiation	7.20E-04	9.91E-03	Process	6.57E+00	Myc;Rrn3;Taf9b
GO:0071499	cellular response to laminar fluid shear stress	2.54E-04	1.01E-02	Process	1.24E+01	Nfe2l2;Klf2
GO:0051168	nuclear export	2.54E-04	1.01E-02	Process	1.24E+01	Malt1;Lsg1
GO:0044539	long-chain fatty acid import	2.54E-04	1.01E-02	Process	1.24E+01	Slc27a2;Acsl3
GO:0044806	G-quadruplex DNA unwinding	2.54E-04	1.01E-02	Process	1.24E+01	Wrn;Blm
GO:0006207	'de novo' pyrimidine nucleobase biosynthetic process	2.54E-04	1.01E-02	Process	1.24E+01	Cps1;Umps

GO:0033135	regulation of peptidyl-serine phosphorylation	2.54E-04	1.01E-02	Process	1.24E+01	Nsd1;Sptbn4
GO:0031514	motile cilium	3.71E-03	1.04E-02	Component	2.91E+00	Sept4;Intu;Pkd2;Dnah17;Dnah2;Slc9c1;Dync2h1
GO:0000403	Y-form DNA binding	2.62E-04	1.07E-02	Function	1.20E+01	Wrn;Blm
GO:0017171	serine hydrolase activity	2.62E-04	1.07E-02	Function	1.20E+01	Pla2g6;Cela2a
GO:0045505	dynein intermediate chain binding	1.40E-03	1.14E-02	Function	4.51E+00	Dnah17;BC048507;Dync2h1;Dnah2
GO:0003682	chromatin binding	2.04E-02	1.21E-02	Function	1.69E+00	Rbpjl;Sox14;Ybx1;Rnf2;Ctcf;Cited1;Nup153;Mllt3;Bmi1;Pola1;Tada2b;Lmo2;Hesx1;Cdyl;Prop1;Cbx7;Nsd1;Tal1;Kdm4c;Gtf2h1;Wrn;Ezh1
GO:0008270	zinc ion binding	2.91E-02	1.21E-02	Function	1.57E+00	Polr3k;Mmp10;Ptch1;Myt1;Churc1;Rnf2;Agbl3;Pgr;Rnf139;Nup153;Top3a;Trim33;Thra;Ikzf5;Trim13;Sf3a3;Zcchc2;Usp33;Zcchc10;Zfp37;Nsd1;Adamts20;Dhh;Neil2;Esco1;Kdm4c;Blm;Zfand5;Chordc1
GO:0035690	cellular response to drug	2.20E-03	1.27E-02	Process	3.58E+00	Rbm22;Blm;Braf;Nfe2l2;Myc
GO:0051959	dynein light intermediate chain binding	1.44E-03	1.27E-02	Function	4.37E+00	Dnah17;BC048507;Dync2h1;Dnah2
GO:0001502	cartilage condensation	8.05E-04	1.36E-02	Process	5.88E+00	Otor;Thra;Col11a1
GO:0004842	ubiquitin-protein transferase activity	1.47E-02	1.38E-02	Function	1.81E+00	Klhdc8a;Rnf139;Ube3a;Ube2w;Malt1;G2e3;Herc6;Trim13;Klhl29;Lmo7;Rnf103;Ube2c;Cblb;Klhl30;Mphosph8;Rnf2;Fbxl14
GO:0043584	nose development	2.97E-04	1.38E-02	Process	1.06E+01	Hesx1;Rdh10
GO:0045656	negative regulation of monocyte differentiation	2.97E-04	1.38E-02	Process	1.06E+01	Zbtb46;Myc
GO:0009313	oligosaccharide catabolic process	2.97E-04	1.38E-02	Process	1.06E+01	Gm2a;Ctbs

GO:0051480	regulation of cytosolic calcium ion concentration	1.48E-03	1.40E-02	Process	4.25E+00	Trpc1;Tmem178;Cul5;Cdh23
GO:0034366	spherical high-density lipoprotein particle	2.98E-04	1.46E-02	Component	1.03E+01	Apoc3;Pon1
GO:0070765	gamma-secretase complex	2.98E-04	1.46E-02	Component	1.03E+01	Aph1b;Aph1c
GO:0000151	ubiquitin ligase complex	3.96E-03	1.46E-02	Component	2.72E+00	Ccnc;Ube2c;4933402N03Rik;Mphosph8;Rnf2;Hspa8;Bmi1
GO:0051880	G-quadruplex DNA binding	3.05E-04	1.47E-02	Function	1.03E+01	Wrn;Blm
GO:0008479	queuine tRNA-ribosyltransferase activity	3.05E-04	1.47E-02	Function	1.03E+01	Txndc9;Pdcl3
GO:0005113	patched binding	3.05E-04	1.47E-02	Function	1.03E+01	Dhh;Ptch1
GO:0015030	Cajal body	1.49E-03	1.54E-02	Component	4.13E+00	Lsg1;Angel2;Toe1;Npat
GO:0060997	dendritic spine morphogenesis	8.47E-04	1.56E-02	Process	5.58E+00	Ctnnd2;Epha5;Prmt3
GO:0071013	catalytic step 2 spliceosome	4.13E-03	1.81E-02	Component	2.61E+00	Sf3a3;Magohb;Rbm22;Sf3b6;Crnkl1;Rbm8a;Ppwd1
GO:0061820	telomeric D-loop disassembly	3.39E-04	1.81E-02	Process	9.31E+00	Wrn;Blm
GO:0002070	epithelial cell maturation	3.39E-04	1.81E-02	Process	9.31E+00	Pgr;Gja1
GO:0034379	very-low-density lipoprotein particle assembly	3.39E-04	1.81E-02	Process	9.31E+00	Apoc3;Acsl3
GO:0010518	positive regulation of phospholipase activity	3.39E-04	1.81E-02	Process	9.31E+00	Hpca;Pla2g5

GO:0050847	progesterone receptor signaling pathway	3.39E-04	1.81E-02	Process	9.31E+00	Ube3a;Pgr
GO:0048680	positive regulation of axon regeneration	3.39E-04	1.81E-02	Process	9.31E+00	Braf;Mif
GO:0034498	early endosome to Golgi transport	3.39E-04	1.81E-02	Process	9.31E+00	Snx1;Rbsn
GO:0071481	cellular response to X-ray	3.39E-04	1.81E-02	Process	9.31E+00	Ccnd2;Sfrp1
GO:0048845	venous blood vessel morphogenesis	3.39E-04	1.81E-02	Process	9.31E+00	Notch4;Ccm2
GO:0030496	midbody	5.11E-03	1.84E-02	Component	2.41E+00	Uvrag;Cep55;Ptch1;Aurkb;Lyrm1;Txndc9;Cit;Sept1
GO:0007339	binding of sperm to zona pellucida	1.61E-03	1.85E-02	Process	3.92E+00	Cct6a;Zan;Cct4;Cct7
GO:0008380	RNA splicing	1.06E-02	1.87E-02	Process	1.93E+00	Dcps;Wtap;Ncbp2;Sf3a3;Thoc2;Ybx1;Magohb;Rbm22;Hspa8;Sf3b6;Crnkl1;Lsm5;Ppwd1
GO:0072669	tRNA-splicing ligase complex	3.41E-04	1.91E-02	Component	9.04E+00	Zbtb8os;AI597479
GO:0001164	RNA polymerase I CORE element sequence-specific DNA binding	3.49E-04	1.92E-02	Function	9.02E+00	Taf1b;Rrn3
GO:0043140	ATP-dependent 3'-5' DNA helicase activity	3.49E-04	1.92E-02	Function	9.02E+00	Wrn;Blm
GO:0102391	decanoate--CoA ligase activity	3.49E-04	1.92E-02	Function	9.02E+00	Slc27a2;Acsl3
GO:0004033	aldo-keto reductase (NADP) activity	3.49E-04	1.92E-02	Function	9.02E+00	Akr1c20;Akr1c13

GO:0008035	high-density lipoprotein particle binding	3.49E-04	1.92E-02	Function	9.02E+00	Pon1;Hspd1
GO:0008020	G-protein coupled photoreceptor activity	3.49E-04	1.92E-02	Function	9.02E+00	Rgr;Opn4
GO:0035145	exon-exon junction complex	8.95E-04	1.93E-02	Component	5.17E+00	Pym1;Rbm8a;Magohb
GO:0008569	ATP-dependent microtubule motor activity, minus-end-directed	9.16E-04	1.95E-02	Function	5.15E+00	Dnah17;Dync2h1;Dnah2
GO:0043085	positive regulation of catalytic activity	1.65E-03	2.02E-02	Process	3.82E+00	Aph1b;Aph1c;Myc;Hspa8
GO:0061512	protein localization to cilium	9.32E-04	2.03E-02	Process	5.08E+00	Bbs9;Dync2h1;Tulp4
GO:0050821	protein stabilization	6.44E-03	2.19E-02	Process	2.20E+00	Creb1;Cct6a;Hsp90aa1;Cct7;Usp33;Cct4;Bag3;Hspd1;Taf9b
GO:0003824	catalytic activity	2.04E-02	2.22E-02	Function	1.61E+00	St6gal2;Pklr;Enpp6;Azin1;Ppm1a;Ganc;Npl;Cps1;Ugdh;Cdyl;Ckb;Mut;Slc27a2;Neil2;Cth;Umps;Acsl3;Auh;Wrn;Blm;Prkg1
GO:0009048	dosage compensation by inactivation of X chromosome	3.81E-04	2.29E-02	Process	8.27E+00	Eif1;Ctcf
GO:0014050	negative regulation of glutamate secretion	3.81E-04	2.29E-02	Process	8.27E+00	Prkg1;Htr6
GO:0034374	low-density lipoprotein particle remodeling	3.81E-04	2.29E-02	Process	8.27E+00	Pla2g5;Mpo
GO:0080009	mRNA methylation	3.81E-04	2.29E-02	Process	8.27E+00	Mettl3;Wtap

GO:0042994	cytoplasmic sequestering of transcription factor	3.81E-04	2.29E-02	Process	8.27E+00	Thra;Pkd2
GO:0043030	regulation of macrophage activation	3.81E-04	2.29E-02	Process	8.27E+00	Shpk;Pla2g5
GO:0030814	regulation of cAMP metabolic process	3.81E-04	2.29E-02	Process	8.27E+00	Cep290;Pkd2
GO:0006032	chitin catabolic process	3.81E-04	2.29E-02	Process	8.27E+00	Chil6;Ctbs
GO:0032926	negative regulation of activin receptor signaling pathway	3.81E-04	2.29E-02	Process	8.27E+00	Lemd3;Magi2
GO:2000234	positive regulation of rRNA processing	3.81E-04	2.29E-02	Process	8.27E+00	Heatr1;Riok1
GO:0002329	pre-B cell differentiation	3.81E-04	2.29E-02	Process	8.27E+00	Cd24a;Atp11c
GO:0045494	photoreceptor cell maintenance	1.74E-03	2.38E-02	Process	3.63E+00	Cep290;Rp1;Lca5;Cdh23
GO:0004568	chitinase activity	3.92E-04	2.43E-02	Function	8.01E+00	Chil6;Ctbs
GO:0042623	ATPase activity, coupled	3.92E-04	2.43E-02	Function	8.01E+00	Pex1;Hspa8
GO:0000340	RNA 7-methylguanosine cap binding	3.92E-04	2.43E-02	Function	8.01E+00	Dcps;Ncbp2
GO:0000398	mRNA splicing, via spliceosome	4.49E-03	2.43E-02	Process	2.46E+00	Mettl3;Ncbp2;Sf3a3;Pspc1;Sf3b6;Crnkl1;Lsm5
GO:0004402	histone acetyltransferase activity	1.74E-03	2.43E-02	Function	3.61E+00	Cdyl;Elp4;Tada2b;Taf9b

GO:0005509	calcium ion binding	2.57E-02	2.45E-02	Function	1.53E+00	Vldlr;Canx;Stab1;Pon1;Pkd2;Hpca;Actn2;Fstl5;Slc25a25;Umod;Rptn;Pla2g5;Cblb;Cps1;Padi6;Stab2;Notch4;Myl1;Anxa10;Dhh;Atp2c1;Syt14;Braf;Chordc1;Cdh23
GO:0008023	transcription elongation factor complex	9.80E-04	2.47E-02	Component	4.72E+00	Mllt3;Elp4;Eaf1
GO:0008139	nuclear localization sequence binding	1.00E-03	2.49E-02	Function	4.70E+00	Nup153;Kpnb1;Kpna4
GO:0007159	leukocyte cell-cell adhesion	1.02E-03	2.57E-02	Process	4.65E+00	Cd24a;Umod;Vcam1
GO:0050856	regulation of T cell receptor signaling pathway	4.24E-04	2.81E-02	Process	7.44E+00	Malt1;Cblb
GO:0061337	cardiac conduction	4.24E-04	2.81E-02	Process	7.44E+00	Sptbn4;Gja1
GO:0046632	alpha-beta T cell differentiation	4.24E-04	2.81E-02	Process	7.44E+00	Braf;Blm
GO:0007220	Notch receptor processing	4.24E-04	2.81E-02	Process	7.44E+00	Aph1b;Aph1c
GO:0031145	anaphase-promoting complex-dependent catabolic process	4.24E-04	2.81E-02	Process	7.44E+00	Anapc10;Ube2c
GO:0051591	response to cAMP	1.06E-03	2.86E-02	Process	4.47E+00	Braf;Pklr;Cited1
GO:0031018	endocrine pancreas development	1.06E-03	2.86E-02	Process	4.47E+00	Rfx6;Onecut2;Myt1
GO:0005975	carbohydrate metabolic process	7.88E-03	2.92E-02	Process	2.00E+00	St6gal2;Ganc;Pklr;Ctbs;Gnpda2;Ppp1r2;Npl;Shpk;Glb1l2;Pdha2
GO:0005041	low-density lipoprotein receptor activity	4.36E-04	2.98E-02	Function	7.21E+00	Stab1;Stab2
GO:0009881	photoreceptor activity	4.36E-04	2.98E-02	Function	7.21E+00	Rgr;Opn4

GO:0031519	PcG protein complex	1.07E-03	3.08E-02	Component	4.34E+00	Bmi1;Rnf2;Cbx7
GO:0015031	protein transport	2.51E-02	3.09E-02	Process	1.51E+00	Btf3;Pex1;Tmed5;Lin7c;Tom1l1;Lsg1;Vps13a;Cep290;Bbs9;Kpnb1;Slc15a4;Appbp2;Snx24;Ap3m2;Snx16;Snx1;Rbsn;Ipo7;Timm9;Lca5;Copg1;Nup37;Vps37a;Kpna4
GO:0004623	phospholipase A2 activity	1.09E-03	3.10E-02	Function	4.33E+00	Pla2g5;Pla2g6;Plb1
GO:0007286	spermatid development	3.73E-03	3.12E-02	Process	2.54E+00	Sept4;Strbp;Dhh;Trip13;Cdyl;Pygo1
GO:0003723	RNA binding	6.28E-02	3.14E-02	Function	1.30E+00	Cct6a;Mettl3;Strbp;Btf3;Pus7;Hspe1;Gspt1;Ybx1;Nol10;Cct4;Rbm22;Crnkl1;Canx;Gm5093;Rpl39;Lsm5;Stip1;Ythdf1;Sf3b6;Heatr1;Kpnb1;Hspd1;Safb2;Kctd12;Nol11;Luc7l2;Sf3a3;Trmo;Thoc2;Dhx33;Brix1;Mrpl32;Eif1ax;Esf1;Krr1;Rps12;Gar1;Pym1;Pspc1;Hspa8;Cbx7;Igf2bp3;Zfp106;Hsp90aa1;Ncbp2;Exosc3;Auh;Magohb;Khdrbs2;Fubp3;Rbm8a;Khdc1b
GO:0043231	intracellular membrane-bounded organelle	3.35E-02	3.15E-02	Component	1.42E+00	Ptch1;Lamp3;Dennd6a;Ybx1;Ugt2b38;Fmo5;Ppp6r2;Tax1bp3;Fosl1;Pon1;Rdh10;Hspd1;Safb2;Btaf1;Snx16;Snx1;Rbsn;Ugt2a3;Mpo;Eaf1;Slc27a2;Ugt2b34;Gpbp1;Mpv17l;Acsl3;Atp2c1;Braf;Vps37a;Cyp2f2;Trappc2l;Pdha2
GO:0051087	chaperone binding	3.75E-03	3.23E-02	Function	2.52E+00	Hspe1;Stip1;Ahsa2;Bag3;Hspd1;Timm9
GO:0006397	mRNA processing	1.39E-02	3.34E-02	Process	1.70E+00	Dcps;Mettl3;Wtap;Ncbp2;Sf3a3;Thoc2;Ybx1;Magohb;Rbm22;Khdrbs2;Hspa8;Sf3b6;Crnkl1;Lsm5;Ppwd1
GO:0001763	morphogenesis of a branching structure	4.66E-04	3.38E-02	Process	6.77E+00	Ctnnd2;Notch4
GO:0046641	positive regulation of alpha-beta T cell proliferation	4.66E-04	3.38E-02	Process	6.77E+00	Cd24a;Blm
GO:0033603	positive regulation of dopamine secretion	4.66E-04	3.38E-02	Process	6.77E+00	Htr6;Chrnb2
GO:0032816	positive regulation of natural killer cell activation	4.66E-04	3.38E-02	Process	6.77E+00	Il12a;Raet1b

GO:2000009	negative regulation of protein localization to cell surface	4.66E-04	3.38E-02	Process	6.77E+00	Actn2;Tax1bp3
GO:0070613	regulation of protein processing	4.66E-04	3.38E-02	Process	6.77E+00	Notch4;Rnf139
GO:0018298	protein-chromophore linkage	4.66E-04	3.38E-02	Process	6.77E+00	Rgr;Opn4
GO:0004553	hydrolase activity, hydrolyzing O-glycosyl compounds	1.13E-03	3.44E-02	Function	4.16E+00	Ganc;Glb1l2;Ctbs
GO:0070979	protein K11-linked ubiquitination	1.14E-03	3.50E-02	Process	4.14E+00	Anapc10;Ube2w;Ube2c
GO:0035102	PRC1 complex	4.69E-04	3.56E-02	Component	6.58E+00	Rnf2;Cbx7
GO:0017069	snRNA binding	4.80E-04	3.58E-02	Function	6.56E+00	Toe1;Ncbp2
GO:0016772	transferase activity, transferring phosphorus-containing groups	4.80E-04	3.58E-02	Function	6.56E+00	Ckb;Fpgt
GO:0008026	ATP-dependent helicase activity	4.80E-04	3.58E-02	Function	6.56E+00	Wrn;Blm
GO:0008061	chitin binding	4.80E-04	3.58E-02	Function	6.56E+00	Chil6;Ctbs
GO:0070410	co-SMAD binding	4.80E-04	3.58E-02	Function	6.56E+00	Cited1;Trim33
GO:0001228	transcriptional activator activity, RNA polymerase II transcription regulatory region	5.93E-03	3.60E-02	Function	2.12E+00	Rfx6;Rbpjl;Ctcf;Ikzf3;Myt1;Myf6;Lmo2;Zfp131

	sequence-specific DNA binding					
GO:0030154	cell differentiation	3.61E-02	3.69E-02	Process	1.40E+00	Mettl3;Strbp;Sema4g;Myt1;Morn2;Tff1;Sfrp1;Creb1;Tle3;Cited1;Adam26a;Zfp281;Ift81;Trip13;Adcyap1r1;Rfx6;Sema3a;Sept4;Usp42;Myf6;Irf6;Notch4;Cdyl;Slc9c1;Zfp37;Tal1;Cplx2;Prkx;Zfp521;Cit;Braf;Txndc2
GO:0045429	positive regulation of nitric oxide biosynthetic process	1.99E-03	3.71E-02	Process	3.17E+00	Raet1b;Klf2;Hsp90aa1;Pkd2
GO:0036126	sperm flagellum	1.96E-03	3.78E-02	Component	3.15E+00	Sept4;Tekt5;Hsp90aa1;Txndc2
GO:0050840	extracellular matrix binding	1.18E-03	3.79E-02	Function	4.01E+00	Olfml2a;Bgn;Zan
GO:0007283	spermatogenesis	1.67E-02	3.86E-02	Process	1.61E+00	Mif;Tle3;Mettl3;Strbp;Sept4;Adam26a;Usp42;Morn2;Zfx;Ift81;Slc9c1;Cit;Trip13;Cdyl;Txndc2;Zfp37;Adcyap1r1
GO:0000978	RNA polymerase II proximal promoter sequence-specific DNA binding	1.92E-02	3.90E-02	Function	1.56E+00	Creb1;Rfx6;Nsd1;Fosl1;Rbpjl;Pgr;Tal1;Atf4;Ctcf;Zfp281;Fubp3;Ybx1;Myf6;Bsx;Hesx1;Klf16;Onecut2;Myc;Bmi1
GO:0002237	response to molecule of bacterial origin	5.08E-04	3.98E-02	Process	6.20E+00	Malt1;Cd24a
GO:0045577	regulation of B cell differentiation	5.08E-04	3.98E-02	Process	6.20E+00	Cd24a;Ikzf3
GO:0046548	retinal rod cell development	5.08E-04	3.98E-02	Process	6.20E+00	Rp1;Miat
GO:0060644	mammary gland epithelial cell differentiation	5.08E-04	3.98E-02	Process	6.20E+00	Irf6;Ptch1

GO:0035970	peptidyl-threonine dephosphorylation	5.08E-04	3.98E-02	Process	6.20E+00	Dusp5;Ppm1a
GO:0044237	cellular metabolic process	5.08E-04	3.98E-02	Process	6.20E+00	Wrn;Blm
GO:0021952	central nervous system projection neuron axonogenesis	5.08E-04	3.98E-02	Process	6.20E+00	Sptbn4;Chrnb2
GO:0043235	receptor complex	5.96E-03	4.10E-02	Component	2.07E+00	Pigr;Lepr;Kctd12;Vldlr;Impg2;Plxdc1;Trpc1;Adcyap1r1
GO:0005719	nuclear euchromatin	1.19E-03	4.13E-02	Component	3.88E+00	Creb1;Myc;Cecr2
GO:0001824	blastocyst development	1.23E-03	4.20E-02	Process	3.85E+00	N4bp2l2;Thoc2;G2e3
GO:0017017	MAP kinase tyrosine/serine/threonine phosphatase activity	5.23E-04	4.21E-02	Function	6.01E+00	Dusp5;Dusp1
GO:0042752	regulation of circadian rhythm	2.08E-03	4.22E-02	Process	3.04E+00	Creb1;Ube3a;Opn4;Pspc1
GO:0005681	spliceosomal complex	6.05E-03	4.39E-02	Component	2.04E+00	Rheb;Sf3a3;Rbm22;Hspa8;Sf3b6;Crnkl1;Lsm5;Ppwd1
GO:0046982	protein heterodimerization activity	2.47E-02	4.48E-02	Function	1.47E+00	Mettl3;Gtf2a1;Myc;Sox14;Ralgapa2;Uba3;Il12a;Taf9b;Nfe2l2;Hspd1;Thra;Pola1;Snx1;Nfyb;Tal1;Atf4;Ikzf3;Syt14;Khdrbs2;Braf;Cul5;Lsm5;Chrnb2
GO:0003677	DNA binding	7.56E-02	4.49E-02	Function	1.25E+00	Gtf2a1;Strbp;Rbpjl;Dlx6;Mcm8;Sox14;Zfp518a;Ybx1;Churc1;Myt1;Acd;Zfp329;Zfx;Klf16;Onecut2;Sp100;Creb1;Fosl1;Pgr;Ctcf;Adnp2;Rev1;Zfp281;Taf1b;Rfxap;Top3a;Bsx;Zfp131;Nfe2l2;Trim33;Rfx6;Thra;Ikzf5;Safb2;Pola1;Pou6f1;Myf6;Lmo2;Irf6;Hesx1;Zfp37;Prop1;Lemd3;Nfyb;Neil2;Tal1;Atf4;Ikzf3;Creb5;Zfp60;Klf2;Gpbp1;Zfp521;Zfp322a;Polr1d;Blm;Wrn;Zfp654;Zfand5;Myc
GO:0016604	nuclear body	1.18E-02	4.53E-02	Component	1.70E+00	Safb2;Toe1;Pou6f1;Lsg1;Eaf1;Acd;Rnf2;Miat;Cdyl;Med19;Myc;Ppwd1;Bmi1

GO:0045503	dynein light chain binding	1.26E-03	4.55E-02	Function	3.73E+00	Dnah17;Dync2h1;Dnah2
GO:0015020	glucuronosyltransferase activity	1.26E-03	4.55E-02	Function	3.73E+00	Ugt2b34;Ugt2a3;Ugt2b38
GO:0006828	manganese ion transport	5.51E-04	4.62E-02	Process	5.73E+00	Atp2c1;Trpc1
GO:0043268	positive regulation of potassium ion transport	5.51E-04	4.62E-02	Process	5.73E+00	Mif;Actn2
GO:0043616	keratinocyte proliferation	5.51E-04	4.62E-02	Process	5.73E+00	Irf6;Ptch1
GO:0042438	melanin biosynthetic process	5.51E-04	4.62E-02	Process	5.73E+00	Cited1;Trpc1
GO:0016574	histone ubiquitination	5.51E-04	4.62E-02	Process	5.73E+00	Rnf2;Bmi1
GO:0021795	cerebral cortex cell migration	5.51E-04	4.62E-02	Process	5.73E+00	Htr6;Cx3cr1
GO:0043508	negative regulation of JUN kinase activity	5.51E-04	4.62E-02	Process	5.73E+00	Dusp19;Sfrp1
GO:0045580	regulation of T cell differentiation	5.51E-04	4.62E-02	Process	5.73E+00	Braf;Mettl3
GO:0030514	negative regulation of BMP signaling pathway	2.16E-03	4.78E-02	Process	2.92E+00	Lemd3;Ppm1a;Sfrp1;Trim33
GO:0007155	cell adhesion	2.23E-02	4.82E-02	Process	1.48E+00	Siglecg;Tm9sf4;Tnfaip6;Pcdh17;Vcam1;Ninj2;Emb;Stab1;Sdk1;Cx3cr1;Pcdhb12;Parvb;Mybpc2;Hapln4;Stab2;Ctnnd2;Zan;Cd24a;Cd96;Prkx;Cdh23
GO:1990124	messenger ribonucleoprotein complex	5.54E-04	4.87E-02	Component	5.56E+00	Ybx1;Hspa8

GO:0071782	endoplasmic reticulum tubular network	5.54E-04	4.87E-02	Component	5.56E+00	Kpnb1;Reep5
GO:0005641	nuclear envelope lumen	5.54E-04	4.87E-02	Component	5.56E+00	Cacybp;Hrasls
GO:0042805	actinin binding	5.67E-04	4.89E-02	Function	5.55E+00	Pkd2;Lmo7
GO:0004467	long-chain fatty acid-CoA ligase activity	5.67E-04	4.89E-02	Function	5.55E+00	Slc27a2;Acsl3
GO:0048156	tau protein binding	5.67E-04	4.89E-02	Function	5.55E+00	Dyrk1a;Hsp90aa1
GO:0005654	nucleoplasm	7.74E-02	4.94E-02	Component	1.23E+00	Gtf2a1;Toe1;Ccnd2;Myt1;Rbak;Zfx;Cct4;Rnf2;Ctr9;Zfp438;Onecut2;Creb1;Tle3;Fosl1;Ctcf;Aprt;Zfp281;Npat;Brd8;Zfp131;Nfe2l2;Kpnb1;Mif;Sept4;Safb2;Btaf1;Pola1;Cacybp;Sf3a3;Lyrm1;Elp4;Dhx33;Usp33;Cblb;Arhgap27;Pspc1;Ipo7;Ugdh;Cdyl;Myo16;Eaf1;Dcps;Dennd2d;Cbx7;Ankrd12;Wtap;Ezh1;Aurkb;Ncbp2;Ikzf3;Exosc3;Ubqln1;Nup37;Dgki;Ppp1r2;Itgb3bp;Gtf2h1;Wrn;Prkg1;Vps37a;Myc;Kpna4
GO:0001701	in utero embryonic development	1.22E-02	4.96E-02	Process	1.67E+00	Amot;Fosl1;Btf3;Ptch1;Gja1;Klf2;N4bp2l2;Ybx1;Rdh10;Ccm2;Zfand5;Rrn3;Bmi1
GO:0006950	response to stress	1.31E-03	4.97E-02	Process	3.60E+00	Ahsa2;Hsp90aa1;Alk

Table S4. Differentially expressed protein in S-EV-treated HUVECs.

**GeneSymbol**	**GeneName**	**Description**	**RefSeq Accession**	**EnsemblID**	**Fold (IH SEV vs.N SEV)**	**Regulation (IH SEV vs.N SEV)**	**p value**	**IH SEV1**	**IH SEV2**	**IH SEV3**	**Nor SEV1**	**Nor SEV2**	**Nor SEV3**
		heterogeneous nuclear ribonucleoprotein A3 pseudogene 12		ENST00000437410	-2.078	down	0.006	6.935	7.045	6.979	8.286	7.649	8.190

TMEM33	transmembrane protein 33	Homo sapiens transmembrane protein 33	NM_018126	ENST00000325094	-2.040	down	0.046	5.624	5.691	5.914	7.129	6.072	7.113
ARHGEF35	Rho guanine nucleotide exchange factor (GEF) 35	Homo sapiens Rho guanine nucleotide exchange factor	NM_001003702	ENST00000378115	-2.087	down	0.030	7.238	7.491	7.266	8.636	7.774	8.769
UBXN8	UBX domain protein 8	Homo sapiens UBX domain protein 8	NM_001282199	ENST00000518059	2.216	up	0.000	8.555	8.526	8.677	7.426	7.442	7.445
TAOK2	TAO kinase 2	Homo sapiens TAO kinase 2	NM_004783	ENST00000279394	2.037	up	0.001	6.516	6.450	6.470	5.574	5.551	5.231
RAB13	RAB13, member RAS oncogene family	Homo sapiens RAB13, member RAS oncogene family	NM_002870	ENST00000484297	-2.082	down	0.002	9.962	10.108	10.117	11.236	10.827	11.299
		heterogeneous nuclear ribonucleoprotein A1 pseudogene 36		ENST00000521313	-2.003	down	0.006	9.088	9.318	9.079	10.233	9.841	10.417
lnc-ZNF91-2	lnc-ZNF91-2:1	LNCipedia lincRNA			-3.249	down	0.024	6.180	6.255	6.089	8.334	6.924	8.366
DCAF8	DDB1 and CUL4 associated factor 8	Homo sapiens DDB1 and CUL4 associated factor 8	NM_015726	ENST00000407642	-2.232	down	0.000	6.682	6.858	6.771	8.001	7.839	7.947
		heterogeneous nuclear ribonucleoprotein A1 pseudogene 60		ENST00000526810	-2.120	down	0.011	7.280	7.483	7.196	8.526	7.964	8.722
CCDC71	coiled-coil domain containing 71	Homo sapiens coiled-coil domain containing 71	NM_022903	ENST00000321895	2.161	up	0.001	8.941	8.613	8.624	7.644	7.708	7.492

AKAP12	A kinase (PRKA) anchor protein 12	Homo sapiens A kinase (PRKA) anchor protein 12	NM_144497	ENST00000490177	-2.277	down	0.000	12.374	12.448	12.517	13.742	13.460	13.698
lnc-NKD2-3	lnc-NKD2-3:7	LNCipedia lincRNA			-3.736	down	0.003	3.599	4.429	4.027	6.007	5.594	6.158
CDK11A	cyclin-dependent kinase 11A	Homo sapiens cyclin-dependent kinase 11A (CDK11A)	NM_033529	ENST00000401096	-2.027	down	0.001	9.155	9.337	9.341	10.422	10.076	10.392
ND6	NADH dehydrogenase, subunit 6 (complex I)	mitochondrially encoded NADH dehydrogenase 6		ENST00000361681	-3.965	down	0.015	5.667	5.351	5.382	7.635	6.555	8.172
PEG10	paternally expressed 10	Homo sapiens paternally expressed 10	NM_001040152	ENST00000493935	-3.521	down	0.000	8.683	8.889	8.619	10.691	10.234	10.714
DNAJC7	DnaJ (Hsp40) homolog, subfamily C, member 7	Homo sapiens DnaJ (Hsp40) homolog, subfamily C, member 7	NM_003315	ENST00000316603	2.197	up	0.001	11.865	11.534	11.739	10.505	10.711	10.515
HIST1H2BE	histone cluster 1, H2be	Homo sapiens histone cluster 1, H2be	NM_003523		-2.443	down	0.005	6.026	6.544	6.457	7.607	7.362	7.924
NFE2L2	nuclear factor, erythroid 2-like 2	Homo sapiens nuclear factor, erythroid 2-like 2	NM_006164	ENST00000464747	-2.373	down	0.004	2.788	2.966	2.630	3.373	3.305	4.304

Table S5. Blood metabolic indexes of participants with or without OSA.

	Non-OSA (n=6)	OSA (n=6)	P value
Age (years)	44.8±13.6	45.2±14.7	0.94
Gender (male, %)	100	100	-
Obstructive AHI (events/hour)	4.1±0.9	39.4±22.7	0.02

SaO_2 (%)	91.1±1.6	80.8±3.9	
Systolic blood pressure (mmHg)	11.4±8.38	125±11.3	0.25
Diastolic blood pressure (mmHg)	80.8±6.6	76.8±8.2	0.52
Total cholesterol (mmol/L)	4.3±0.5	5.1±0.6	0.06
HDL cholesterol (mmol/L)	1.4±0.2	1.3±0.2	0.24
LDL cholesterol (mmol/L)	2.6±0.8	3.6±0.2	0.09
Triglycerides (mmol/L)	1.0±0.2	1.2±0.4	0.51
Blood glucose (mmol/L)	5.3±0.5	5.0±0.9	0.39
Body mass index (BMI)	23±2.0	24.8±4.2	0.33
CRP	0.928±0.66	1.58±1.52	0.39

Table S6. **mRNA and pri-mRNA primer sequence.**

Nox2	Forward primer	AACCCTCCTATGACTTGGAAATG
	Reverse primer	TTGGATACCTTGGGGCACTT
$p47^{phox}$	Forward primer	TGGTGGGTGGTCAGGAAAGG
	Reverse primer	AGGAATCGGACGCTGTTGC
Nox4	Forward primer	GTGTCTGCATGGTGGTGGTATT
	Reverse primer	CAGCCAGGAGGGTGAGTGTC
Xdh	Forward primer	CACAGAACACCATGAAAACCCA
	Reverse primer	GAAGCCCACCTTGTATTTAGCC
β-actin	Forward primer	GCATTGTTACCAACTGGGACG
	Reverse primer	CAGAGGCATACAGGGACAGCA
Pri-miR-144	Forward primer	GACCCTGAGCCAATCCCC
	Reverse primer	CGGTTTCCTCGCCATTCC
Pri-miR-27a	Forward primer	GATAGGCAGGCAAGCAAGAAT
	Reverse primer	AGCACAGGGTCAGTTGGAAAT

Reference

1. Zhang HN, Liu J, Qu D, Wang L, Wong CM, Lau CW *et al*. Serum exosomes mediate delivery of arginase 1 as a novel mechanism for endothelial dysfunction in diabetes. P Natl Acad Sci USA. 2018;115:E6927-E6936.
2. Peng L, Li Y, Li X, Du Y, Li L, Hu C *et al*. Extracellular vesicles derived from intermittent hypoxia-treated red blood cells impair endothelial function through regulating enos phosphorylation and et-1 expression. Cardiovasc Drugs Ther. 2020.
